# The transcriptional profiling of human *in vivo*-generated plasma cells identifies selective imbalances in monoclonal gammopathies

**DOI:** 10.1371/journal.pone.0183264

**Published:** 2017-08-17

**Authors:** Luis M. Valor, Beatriz Rodríguez-Bayona, Ana B. Ramos-Amaya, José A. Brieva, Antonio Campos-Caro

**Affiliations:** Unidad de Investigación, Hospital Universitario Puerta del Mar and Instituto de Investigación e Innovación en Ciencias Biomédicas de Cádiz (INiBICA), Cádiz, Spain; Universidad de Palermo, UNITED STATES

## Abstract

Plasma cells (PC) represent the heterogeneous final stage of the B cells (BC) differentiation process. To characterize the transition of BC into PC, transcriptomes from human naïve BC were compared to those of three functionally-different subsets of human *in vivo*-generated PC: i) tonsil PC, mainly consisting of early PC; ii) PC released to the blood after a potent booster-immunization (mostly cycling plasmablasts); and, iii) bone marrow CD138^+^ PC that represent highly mature PC and include the long-lived PC compartment. This transcriptional transition involves subsets of genes related to key processes for PC maturation: the already known protein processing, apoptosis and homeostasis, and of new discovery including histones, macromolecule assembly, zinc-finger transcription factors and neuromodulation. This human PC signature is partially reproduced *in vitro* and is conserved in mouse. Moreover, the present study identifies genes that define PC subtypes (e.g., proliferation-associated genes for circulating PC and transcriptional-related genes for tonsil and bone marrow PC) and proposes some putative transcriptional regulators of the human PC signatures (e.g., OCT/POU, XBP1/CREB, E2F, among others). Finally, we also identified a restricted imbalance of the present PC transcriptional program in monoclonal gammopathies that correlated with PC malignancy.

## Introduction

Plasma cells (PC) represent the final effector phase of the B cells (BC) differentiation process. As such, PC are capable of producing large quantities of antibodies (Ab) (10^6^−10^7^ molecules per h as determined in *in vitro* systems) and, therefore, become the ultimate responsible of the humoral immune response [1–3]. Despite this definite and common role, the PC compartment is far from being homogeneous, and comprises several subsets of cells that differ in the antigen (Ag)-affinity of their synthesized Ab, specific migratory capacity and territory-restricted location, and longevity, indicating the complex biology of the PC maturational process [1,4–9]. Thus, animal models reveal that PC are initially generated in Ag-activated extra-follicular foci and germinal centers (GC) of the secondary lymphoid organs (SLO); the majority of early PC (EPC) are short-lived, and only those bearing Ab that have acquired high-affinity for the Ag are capable of migrating through the circulation, eventually homing into survival niches present in deposit organs such as the bone marrow (BM) [4–9]. These niches supply the factors required for the PC to acquire the long-lived status and, in consequence, this cell subset provides defensive Ab during long periods of time, and even for the whole lifetime [1].

Human PC appear to follow a similar process of maturation. *In vivo*-generated PC are detectable in several human locations. SLO such as tonsil, spleen or lymph nodes, mainly contain PC which are thought to result from the recent Ag-activation of extra-follicular foci and GC; this process gives rise to early short-lived PC as well as to a variable number of resident more mature PC; these two PC subsets can be separately purified from tonsils by using mechanical and enzymatic methods, respectively [10,11]. The human circulating PC compartment is extremely reduced in steady-state and mostly consists of mucose-derived IgA^+^ cells [12]. However, after a booster immunization, a considerable number of specific PC are transiently released to the human blood (BPC); this subset mainly consists of cycling cells (plasmablasts; PB) and is thought to contain the precursors of long-lived PC [13,14]. Human BM PC (BMPC) exhibit features of mature PC, show prolonged survival capacity, and contain *IGVH* genes with the highest number of mutations [13–16]. Recent studies demonstrate that human CD138^+^ BMPC are highly mature and contain long-lived PC generated after viral infections that occurred many years earlier [17].

Several approaches have been used to establish the mRNA signature of normal human PC [14,17–24]. The present transcriptomic study attempts to improve and complete these results by using the following criteria: i) the analysis only include human *in vivo*-generated PC, as they are reasonably closer to the PC biology; ii) several functionally-different *in vivo*-generated PC subsets are included, in order to cover the complex maturation of PC; and iii) naïve B lymphocytes (BC) are used as reference to establish the PC maturational transition; thus, the essential PC maturational program contains the mRNA changes coincidentally observed in each of the comparisons of BC with the different PC subsets. In accordance, the present study delineates the transcriptomes of three accessible human *in vivo*-generated and functionally-different PC subsets, i.e., tonsil early short-lived PC (TPC) [11], BPC obtained six days after a potent booster immunization [13,14,23], and highly-mature BMPC [17], in comparison with the transcriptome of human naïve BC. As a result, a consistent human PC signature is defined. In addition, the analysis of these three *in vivo*-generated PC subsets reveals new details of the complex maturational process of human PC. Further comparisons with publicly available datasets identify altered expression patterns for specific genes of the normal PC signatures in monoclonal gammopathies, especially in multiple myeloma (MM), suggesting that subtle dysregulation of the PC maturational program may be linked to PC malignancy.

## Materials and methods

### Cell isolation

**Fig 1:**
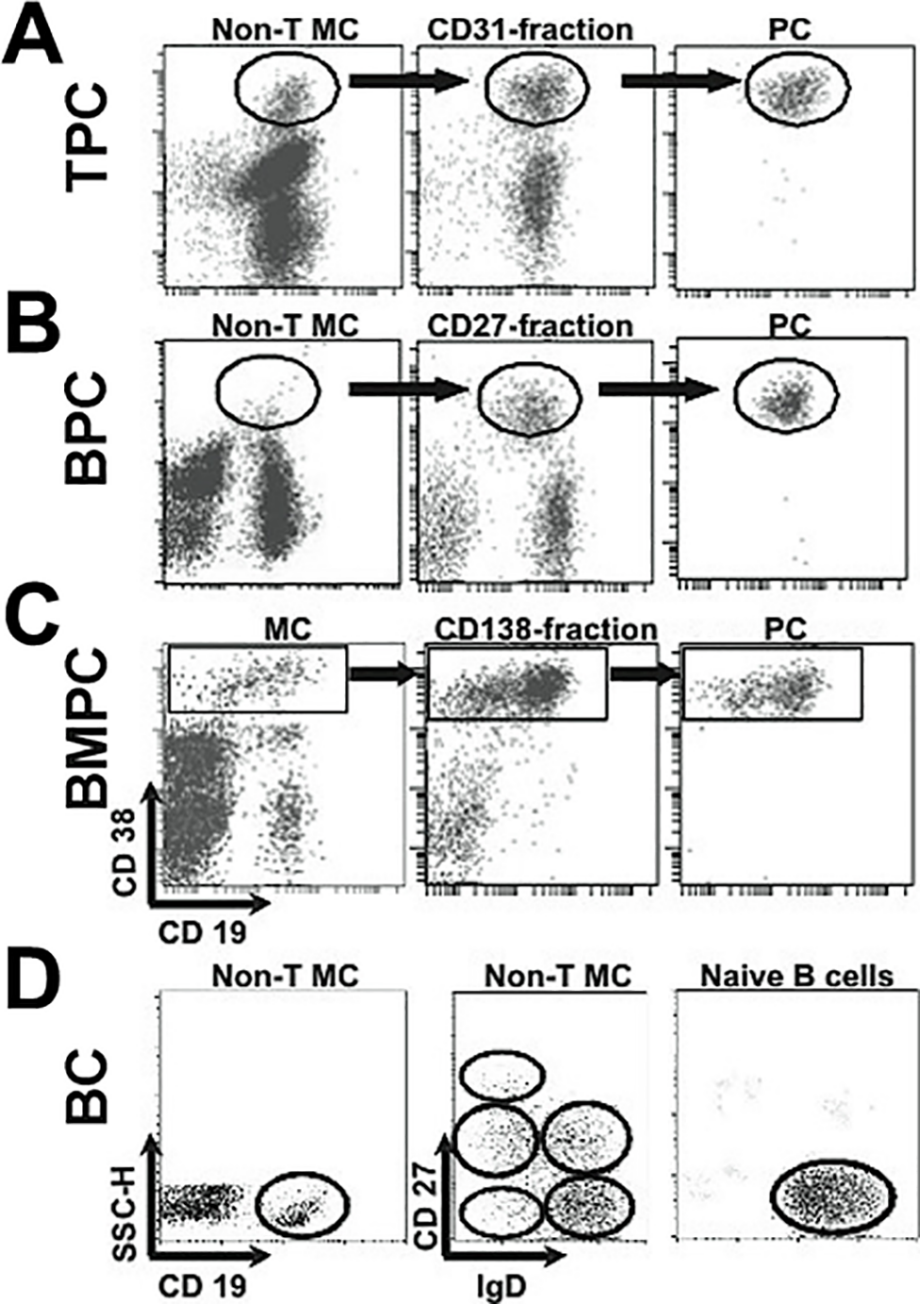
Isolation of human *in vivo*-generated PC and B-cells. **(A)** Tonsil PC (TPC), **(B)** peripheral blood PC (BPC), **(C)** bone marrow PC (BMPC) and **(D)** peripheral blood naïve B cells (BC) were isolated by magnetic selection and flow cytometry sorting according to their surface markers expression as was indicated in Material and methods section. Representative dot plots are depicted.

Human *in vivo*-generated PC were purified from tonsil, BM and blood. Tonsils (T) were obtained from patients undergoing tonsillectomy for recurrent tonsillitis. Peripheral blood samples were obtained from healthy volunteers 6 days after receiving a conventional booster immunization against tetanus-diphtheria toxoids and acellular pertussis (Boostrix; GlaxoSmithKline Biologicals SA; Rixensart, Belgium). Bone marrow (BM) specimens showing no abnormalities were collected from discarded BM aspirates obtained for diagnostic. All samples were obtained from independent individuals. TPC, BPC and BMPC (7, 6 and 7 samples, respectively) were isolated by a combination of immune-magnetic cell-selection and FAC-sorting techniques as previously described [13,16,23]. In brief, tonsil tissue was mechanically disrupted and early TPC were obtained from tonsil cell preparations after T-cell depletion by a rosette technique, pre-enriched by magnetic selection of CD31^+^ cells and FAC sorted as CD19^+^ CD38^+++^ cells (Fig 1A); BPC were obtained from blood cells after a similar T-cell depletion, pre-enriched by magnetic selection of CD27^+^ cells and FAC sorted as CD19^+^ CD38^+++^ cells (Fig 1B); highly-mature BMPC were pre-enriched by magnetic selection of CD138^+^ cells and FAC sorted as CD19^+/-^ CD38^+++^ cells (Fig 1C). PC purity was >97%. The morphological identification as PC of these cell preparations has been previously reported [11,13]. Three samples of human naïve BC were obtained from the blood of normal volunteers; this cell preparation was T-cell depleted by a rosette technique and FAC sorted as CD19^+^ CD27^-^ IgD^+^ (Fig 1D), as previously reported [23], Human BC purity was >99%. All studies were approved by the Institutional Review Board (Comité Ético Hospital Universitario Puerta del Mar) and informed consents were obtained according to the Declaration of Helsinki.

### Real-time quantitative reverse transcribed PCR (RT-qPCR) analysis

Total RNA from newly isolated human BC and PC cellular fractions were purified using the High Pure RNA Isolation Kit (Roche, Barcelona, Spain) and reverse transcribed using random hexamers with the Transcriptor First Strand cDNA Synthesis Kit (Roche). RT-qPCR were performed using the Rotor-Gene Q Detection System (Qiagen Iberia, Madrid, Spain). The Assays-on-Demand™ for *PRDM1*, *PAX5* genes and the TaqMan Universal Master Mix were used according to the manufacturer’s instructions (Applied Biosystems, Foster City, CA). Predesigned primers for gene expression analysis KiCqStart^®^ SYBR^®^ Green Primers (Sigma-Aldrich, St. Louis, MO) and the SensiFAST™ SYBR^®^ No-ROX Kit (Bioline Inc., Taunton, MA) were used for *E2F2*, *IRF4*, *EGR1* and *FOSB* genes. All samples were normalized to *GAPDH* gene and the relative quantitative values were calculated using the 2^-ΔΔCT^ method [25].

### Microarray analysis of cell subpopulations and bioinformatics

Processing of naïve BC and PC samples were conducted in parallel for microarray analysis (RNA extraction, reverse transcription, cDNA labeling, hybridization to One-color Agilent Whole Human Genome Oligo Microarrays 4 × 44K, and chip scanning), as previously described [23]. The array data are deposited in the GEO repository (http://www.ncbi.nlm.nih.gov/geo/) under the accession numbers GSE81589 (for the new data corresponding to naïve BC) and GSE59697 (for the PC samples [23]). The ‘limma’ package [26] from Bioconductor (https://www.bioconductor.org) was used for background correction (“normexp” method, offset = 10) and normalization (“quantile” method) of the data from all the BC and PC samples. Next, a statistical analysis was conducted (based on moderated t tests adjusted with Benjamini and Hochberg's method) for differential expression in pair-wise comparisons. Filtering criteria to define significant differentially expressed genes consisted of an adjusted *P*-value<0.1. No fold change filter was applied.

Other Bioconductor and R packages used were: ‘ArrayQualityMetrics’ for quality microarray assessment [27], ‘prcomp’ function of the built-in R ‘stats’ package for the Principal Component Analysis (PCA), ‘ggplot2’ and ‘rlg’ for PCA plotting [28] (http://cran.r-project.org/package=rgl) and ‘gplots’ package for hierarchical clustering and heatmap representation (http://CRAN.R-project.org/package=gplots). Of the Gene Symbols represented in the array, 3241 were updated using the ‘Multi-symbol checker’ tool, available at the HUGO Gene Nomenclature Committee (HGNC) website (http://www.genenames.org).

**Fig 2:**
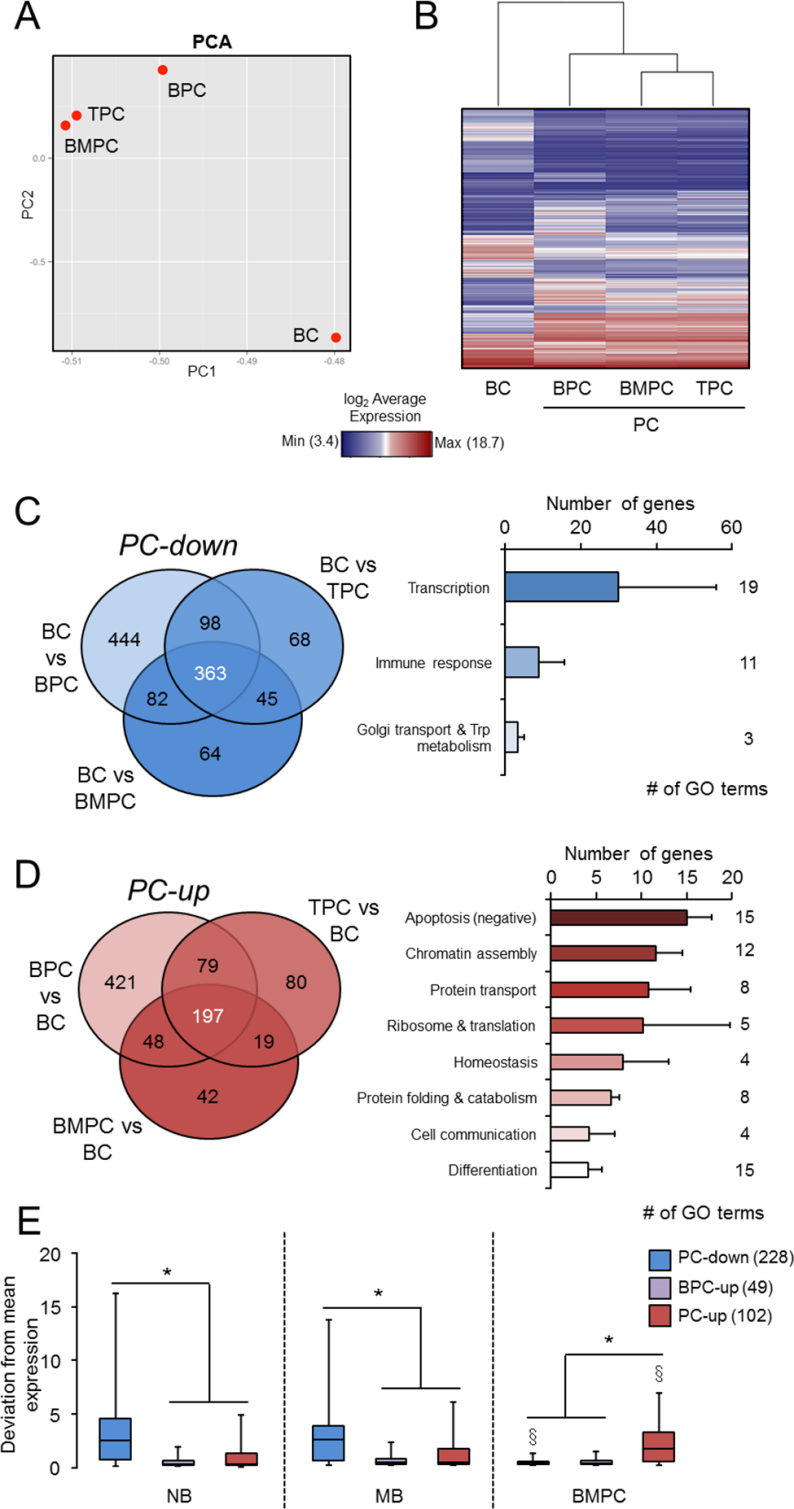
Human B-cell maturation into PC produces distinctive signatures. **(A)** Principal Component Analysis (PCA) of the whole transcriptomes of BC, TPC, BPC and BMPC. **(B)** Heatmap of the average expression of differentially expressed probesets (adj. *P*-value < 0.1) in any pair-wise comparison. **(C)** PC-down signature. Left, Venn diagram showing the down-regulated genes in each PC compared to naïve BC: the central number represents the PC-down signature. Right, GO enrichment analysis of PC-down signature (*P*< 0.05, DAVID), in which the number of genes represents the mean ± SD of the genes contained in the GO terms within the indicated functional categories. **(D)** PC-up signature. Left, Venn diagram showing the up-regulated genes in each PC compared to naïve BC: the central number of the diagram represents the PC-up signature. Right, GO analysis as in (C) for the PC-up signature. **(E)** Box-and-whisker plots of the PC-down, BPC-up and PC-up genes that overlapped with the subset defined in GenomicScape as those genes with the highest deviation from average expression among naïve BC (NB), memory BC (MB) and BMPC from the resource [20]. Numbers of overlapping genes are in brackets in the color legend. For clarity, outliers have been removed. *, *P* < 0.05 in Student’s t-tests between signatures within the same GenomicScape cell subtype; §, *P*< 0.05 Student’s t-tests between each cell subtype and NB within the same signature.

**Fig 3:**
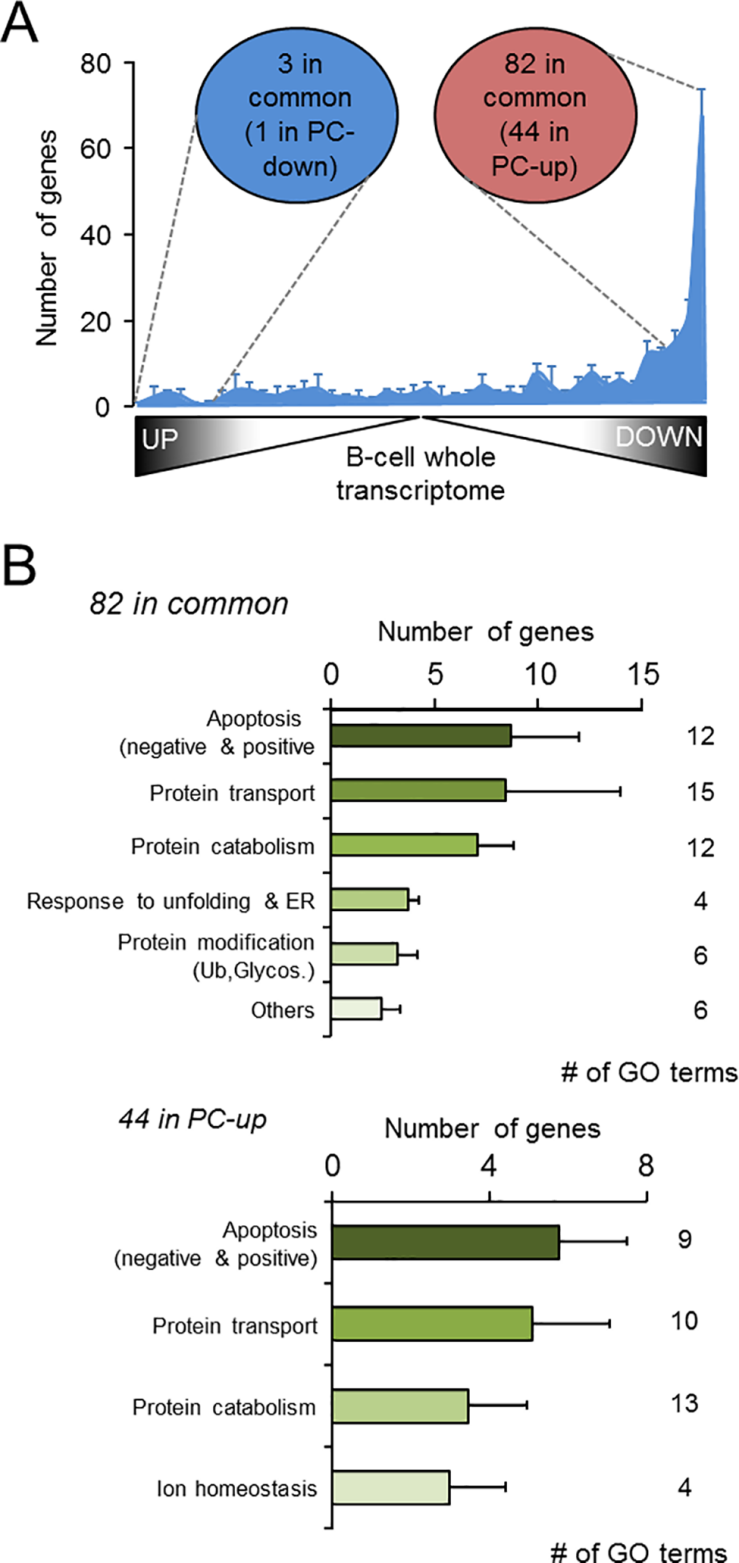
The PC signature is largely conserved in human and mouse. **(A)** Distribution of the genes from the mouse ASC signature previously defined [30] that were unequivocally comparable to human orthologs in the Agilent arrays (final number: 260 genes), across the whole transcriptome of human BC, ordered from more to less significance, without applying any filter. Data are expressed as mean ± SEM of the number of genes in each pair-wise comparison (any PC *vs* BC). The circles indicate the number of genes showing an overlap between the mouse ASC signature and the most up- and down-regulated genes (top 2000) in naïve BC compared to the three PC. In brackets it is denoted the number of overlapping genes with the human PC-down and PC-up signatures. Note that the ASC overlap specifically with the PC-up but not with the PC-down signature, as expected. **(B)** The results of the GO enrichment analysis for the genes in the mouse ASC signature that were either up-regulated genes in human PC (upper panel) or defined in the human PC-up signature (lower panel) are represented as in Fig 2. Most of the enriched functions observed in the human PC-up signature (Fig 2D) are also retrieved in this analysis. Ub, Ubiquitylation, Glycos, Glycosylation.

**Fig 4:**
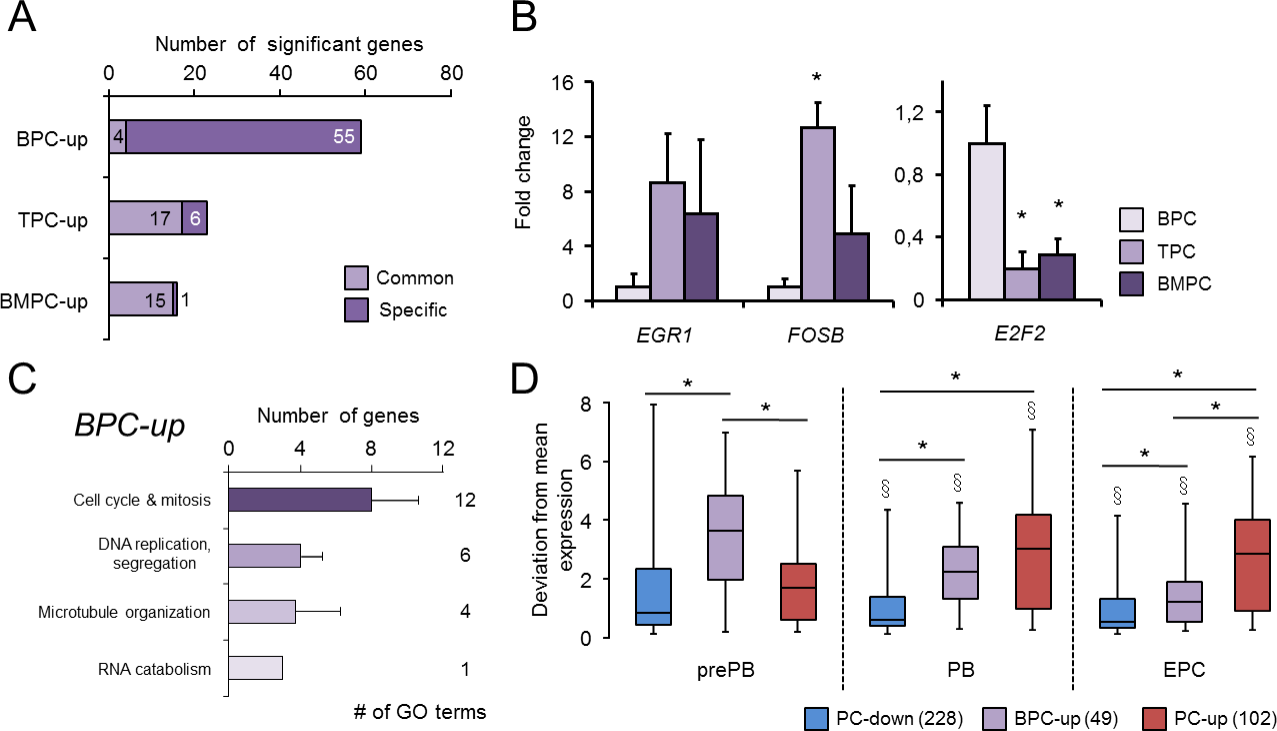
Transcriptional markers of PC subtypes. **(A)** The number of significantly up-regulated genes in one PC-subtype (Specific), or in more than one PC-subtype (Common) are indicated. **(B)** RT-qPCR assays in independent samples (n = 3 for BPC, TPC and n = 4 for BMPC) for selected genes. *, *P* ≤ 0.05, Mann-Whitney test related to BPC. (**C**) GO enrichment analysis for up-regulated genes in BPC (BPC-up) (*P*< 0.05, DAVID) as represented in Fig 2. **(D)** Box-and-whisker plots of the PC-down, BPC-up and PC-up genes that overlapped with the successive stages obtained in an *in vitro* BC differentiation system defined in GenomicScape; this included pre-plasmablasts (prePB), plasmablasts (PB) and early plasma cells (EPC) stages [20]. *, *P* < 0.05 in Student’s t-tests between signatures within the same GenomicScape cell subtype; §, *P*< 0.05 Student’s t-tests between each cell subtype and prePB within the same signature.

Intersections of the resulting lists from the pair-wise comparisons in Venn diagrams (http://bioinfogp.cnb.csic.es/tools/venny/index.html) defined the cell specific signatures: PC associated signatures were the differentially expressed genes retrieved in the three pair-wise comparisons of any PC subtype with BC profile as reference (namely PC-down and PC-up) or retrieved in at least two pair-wise comparisons between PC subtypes (BPC-up, TPC-up and BMPC-up). The web-based tool DAVID (version 6.7) was used for functional GO-based enrichment analysis [29]: only those GO terms with *P*-value < 0.05 were manually classified to get the categories depicted in Figs 2–4.

### Meta-analysis with external transcriptomic datasets of normal BC and PC

In comparative analyses, we used two sources of accessible data. First, we downloaded the values for the subset of genes differentially regulated in the course of human BC maturation to PC by combining *in vivo* and *in vitro* profiles, as compiled in the GenomicScape resource [20] (http://www.genomicscape.com/microarray/nbtopc.php). This subset consisted on 9303 genes that were either up- or down-regulated compared to the overall mean expression in different BC and PC: the *in vivo* cell-subsets analyzed were naïve and memory B cells (NB and MB, respectively) and BMPC; and the *in vitro* cell-subsets were pre-plasmablasts (prePB), plasmablasts (PB) and early plasma cells (EPC), which represent successive stages of PC differentiation according to the GenomicScape resource. Negative values were transformed to positive by using the inverse and setting the value of non-change to 1, in order to allow calculations of basic statistics for box-and-whisker plots.

Second, the set of 301 genes of the murine antibody-secreting cells (ASC) signature was retrieved from S1 Table in reference [30] (http://www.nature.com/ni/journal/v16/n6/full/ni.3154.html#supplementary-information). This set comes from a similar transcriptomic analysis in BC and *in vivo*-generated PC isolated from mouse: the ASC signature was defined as the genes that become over-expressed in mouse splenic plasmablasts (SplPB), splenic PC (SplPC) and BMPC compared to mouse BC, i.e. follicular (FoB), marginal zone (MZB) and germinal center B (GCB) cells. Of this set of genes, 260 were unequivocally comparable to human orthologs in the Agilent arrays by using the HCOP tool, available at the HGNC website. They were mapped across the whole transcriptome of human BC according to the following procedure: after removal of redundant probesets, the BC transcriptome was ordered by the *t* statistic obtained after each pair-wise comparison with any PC subtype to rank the genes from more to less significant considering each direction of change, but without applying any filter; next, the transcriptome was divided in bins of equal number of genes (n = 551) to count the number of ASC genes in each bin; the number of counts was averaged for the three pair-wise comparisons.

### Meta-analysis with external transcriptomic datasets of monoclonal gammopathies

In a first approach, we only considered datasets from monoclonal gammopathies that also included BMPC from healthy donors to better control technical biases between studies. The CEL files were extracted from the accession numbers E-GEOD-6477, E-GEOD-6691, E-GEOD-13591, E-MTAB-363 and E-GEOD-47552 [31–35] and comprised the following PC-related disorders: monoclonal gammopathy of undetermined significance (MGUS), smoldering MM (SMM) and MM. We did not differentiate between MM subtypes (i.e., newly diagnosed or relapsed) as preliminary analyses retrieved highly similar results. Data extraction and normalization was performed using ‘affy’ and ‘oligo’ packages for U133 and 1.0 ST arrays respectively. Only common genes in both Affymetrix and Agilent platforms were considered for further analysis after removal of redundant probesets. Average expression values were analyzed independently for each cell type and for laboratory source, as follows: values were ranked from higher to lower expression, and the whole transcriptomes were divided in bins of equal number of genes; next, we counted the number of PC-down and PC-up genes in each bin, and we calculated the % of the PC genes represented in the arrays. The resulting distribution profiles were merged into the same graphs.

Differential expression analysis (either gammopathy *vs* healthy or MM *vs* MGUS) was conducted using ‘limma’. Due to the use of different microarray platforms, only genes represented in all of them (Human Gene 1.0 ST, Human Genome U133A and U133 Plus 2.0) were analyzed after removal of redundant probesets. To limit the impact on power statistics derived from procedural and sampling differences, we only used the top 1000 genes that were statistically different from normal BMPC (adjusted *P*-value < 0.1) in all the studies. Next, the number of top genes that overlapped with PC signatures was counted for each experiment using ‘Venny’ and the result was then averaged.

In the second approach, only MM-related files in the Human Genome U133 Plus 2.0 format were normalized to calculate the risk factor for short progression-free survival based on the average log_2_ expression of 70 probesets (GEP70 signature), as described elsewhere [36]. Pearson correlation coefficients were calculated between the resulting score and the expression value of each PC signature gene using three independent datasets: E-GEOD-17306, E-MTAB-363, E-GEOD-68891 [34,37,38]. Genes associated with prognosis risk factor were those with correlation coefficients significantly different from zero in the three datasets.

### Transcriptional regulation analysis

Pscan website (www.beaconlab.it/pscan/, [39]) was firstly used to predict the enrichment of transcription factor binding sites (TFBS) in the promoter regions (-450/+50) of the following genes: the 363 genes of PC-down, the 197 genes of PC-up, the 55 specific genes of BPC-up, the 15 common genes to both TPC-up and BMPC-up and the 301 genes of the mouse ASC signature. For each list of genes, all 282 TFBS of TRANSFAC database were ranked according to Z-score values, from more to less significantly enriched. When comparing the predictions from two lists of genes, the rank position of significantly enriched motifs (*P* < 0.05) in one list were tracked by color in the other list. In a second round of analysis, the enriched TFBS in the normal PC subsets were also tracked into the TFBS distribution of MGUS and MM dysregulated genes related to healthy BMPC. We considered as differentially dysregulated genes those appearing in the top 1000 genes in at least two studies; the resulting lists of genes for promoter scanning consisted of 729 genes for MGUS-down, 642 for MGUS-up, 816 for MM-down and 933 for MM-up. To clearly ascribe the distribution patterns to specific predictions, common TFBS between PC-down and PC-up, and between BPC-up and TPC/BMPC-up, were filtered from the analysis.

Putative targets for PRDM1 and XBP1 were obtained from the differentially expressed genes after gene inactivation in BMPC of conditional knockout mice (Prdm1^fl/gfp^CreER^T2^ and Xbp1^fl/fl^Prdm1^+/gfp^CreER^T2^) compared to controls (Prdm1^+/gfp^CreER^T2^ and Xbp1^+/+^Prdm1^+/gfp^CreER^T2^, respectively), as reported in S1 and S3 Tables of the original work [40]. Mouse gene nomenclature was converted into human orthologs using HCOP tool (HGNC website) to enable direct comparisons with the human PC signatures.

## Results

In a previous work using Agilent microarrays, we profiled the gene expression program of human BPC (circulating Ag-induced plasmablasts), and human PC isolated from two different organs: early short-lived TPC and highly-mature CD138^+^ BMPC [23] (Fig 1A–1C). In order to obtain a better definition of the mRNA changes produced during the BC differentiation into PC, the present study includes a comparative analysis with additional transcriptome data from human undifferentiated naïve BC. To this end, these latter cells were also isolated (Fig 1D) and total RNA was purified, reverse transcribed, labeled and hybridized to the gene expression arrays in parallel with those of the previously described PC samples [23].

To facilitate comparisons between the different cell transcriptomes, two types of non-informative probesets were removed before analyses: i) those assigned to immunoglobulins (88 IDs, 0.21% of the total IDs), as these were expected to be highly up-regulated in Ab-secreting PC (see below), allowing to study less obvious changes of gene expression; and ii) those without assignment to any known gene, as they were mainly mapped to multiple locations [41] and therefore, were unreliable (7940 IDs, 19.4% of the total IDs). The remaining 33060 probesets were used in a Principal Component Analysis (PCA) that revealed many similarities between the transcriptomes of TPC and BMPC, followed by BPC; the most divergent cell type was BC (Fig 2A). The degree of similarity between the cell subtypes was confirmed in a hierarchical clustering that only considered the differentially-expressed probesets in all 6 of the possible pair-wise comparisons (n = 2397, adjusted *P*-value < 0.1): BC *vs* BPC, BC *vs* TPC, BC *vs* BMPC, BPC *vs* TPC, BPC *vs* BMPC and TPC *vs* BMPC (Fig 2B). In agreement with the clustering, both the number and magnitude (in terms of significance of the *P*-value) of differentially-expressed genes were consistently higher in the three comparisons involving BC, as we will see in brief. These observations were further corroborated by examining the clustering of individual samples (S1 Fig).

### The human *in vivo*-generated PC signature

To establish a consistent human PC signature, the BC transcriptome was used as reference in the microarray analysis of the three human PC subsets under study. As expected, the three PC subsets showed a strong up-regulation of Ig-related genes such as *IGHG*, *IGHM* and *IGHA* (S2A and S2B Fig). An initial analysis of molecules whose change is known to be relevant in the PC maturational program revealed the expected up-regulation for *PRDM1*, *XBP1*, *MCL1*, *IRF4*, *CD27*, *TNFRSF17* and *SLAMF7* and the down-regulation for *PAX5*, *BACH2*, *CIITA*, *CD22* or *MS4A1* (*CD20*) (S2C Fig) [1,14]. Some of these results (*PRDMI* and *IRF4* up-regulation and *PAX5* down-regulation) were also corroborated by independent qPCRs (S2D Fig). Only genes showing significant changes in the three comparisons were finally ascribed as either down-regulated (363 genes) or up-regulated (197 genes) PC gene signatures (PC-down and PC-up, respectively; Fig 2C and 2D and S1 Table). The GO analysis of PC-down and PC-up genes is summarized in Fig 2C and 2D, and detailed in the Table 1 and S2 Table. Whereas the PC-down genes were mainly associated with transcription and immune response, the PC-up genes showed a more diverse functional enrichment, including apoptosis control, different aspects of protein biology, chromatin assembly, among others. As we will detail in the Discussion, this functional dissociation between the PC-down and PC-up signatures was tightly related with the biology of BC and PC, respectively.

**Table 1:** Functional enrichment analysis of PC signatures.

***PC-down genes***	
Surface markers of B-cell lymphopoiesis	CD34, MS4A1/CD20, CD22, FCER2/CD23, FCRL1, FCRL2
Cytokines and receptors	IL4R, IL-6, TNFSF13B
B-cell chemotaxis and positioning	CCR6, PPBP, GRAP2, ELF4, DOCK11, SKAP1, NOTCH2, TMEM2
B-cell survival	PARP14, ZNF667^a^
BCR function	VAV2, ZNF318, INPP5D
Antigen presentation	HLA class II genes, CIITA, MARCH1, SH2B3, CD83, CD96
B-cell activation	CD69, AFF3, SP2, MAPK1, GNL1, FOXP1, MAVS, CD300LF, MED28, SP100, TRIM22, PER1, SUPT4H1, NOTCH2, HLTF, SMAD3, ZNF160^b^
Complement system	CR1, C5, C6
Transcription factors	ATF71P, NR4A3, TRAK2, CRSNP3, ATXN7, NFIC, ZNF85, ZNF675, PAX4, ZNF347, ZNF254, RNF141, ZNF729, ZNF738, ZNF727, ZNF726, ZNF680, ZNF737, ZNF732, ZNF724P, ZNF493, ZNF43, ZNF93, L3MBTL4, ZNF283, SP100, ZNF479, ZNF440, ZNF813, MBNL1, HLTF, ZNF134, ZNF714, ZNF716, ZNF431, ZNF98, ZNF99, GLIS3, ZNF430, ZNF468, ZNF331, TSC22D2, ZNF709, ZNF429, ZNF223, TFDP2, PEX14, SUPT4H1, ZNF66, ZNF320, ZNF626, ZNF585B, NOTCH2, CSRNP3, DLX6, SP2, ZNF117, ZNF571, HDAC4, HDAC10, NAB2, SMAD3
Neuromodulation	ADRBK2, GABBR1, CNR2, GPR18
Autophagy control	ATG3, DRAM
***PC-up genes***	
Protein translation	RRBP1, RPL13, PABPC4, RPL35, RPL27A, RPS9, EEF2, RPL24, RPS8, RPS28, RPL18A, RPS16, RPL13A, RPLP0EIF3E, RPS15, RPL8, UBC, RPL3, UBB, RPL12, UBA52
Protein localization, transport and secretion	AP1G1, RRBP1, ERP29, PDIA4, LMAN2, CALR, CTNNB1, PREB, HSP90B1, SEC22B, GNAS, SSR4, SEC61A1, SRGN, SSR2, SSR3. NUDT4, GOLGAS, FTH
Unfolding protein response	XBP1, HSP90B1, P4HB, HSPA5, HSPA8, DNAJC3, TOR3A, HSP90B1, PPIB, GRXCR1, ERP29, PDIA5, CALR, FKBP2
Protein catabolism and ER stress response	HM13^c^, SDF2L1^d^, UBC, UBB, UBA52, UFC1 SYVN1, KIAA0368, ARMET^e^, MZB1^f^, MANF^e^
Redox homeostasis	SELS^g^, P4HB, TXNDC11, TXNDC5, GRXCR1, PDIA5, PDIA4
Anti-apoptosis	LOC400750, HSP90B1, SYVN1, TXNDC5, DAD1, UBC, UBB, PIM2, HSPA5, UBA52, CD27, TMBIM6, CTNNB1, CLPTM1L, CDKN1B, DAD1, ACIN1, SRGN, CD27, UBA52, TP53INP1, PIM1, NME1, SLAMF7, CALR
Histones	HIST1H2AD, HIST1H2AM, HIST1H2BC, HIST1H2BD, HIST1H2BE, HIST1H2BG, HIST1H2BI, HIST1H2BJ, HIST1H2BM, HIST1H2BN, HIST2H2BC, HIST2H2BE and HIST3H2BB
Macromolecule assembly	CCDC88C, CDK9, HDCA7, ACIN1, SMARCC2, CTNNB1, RPS15, TUBA4A, PEBP1, RPL24 and CALR

Underlined genes have been curated manually from the literature.

a.- Jiang et al., Plos One (2014) 9:e111653

b.- Takahashi et al., J Immunol (2009) 183:6522.

c.- Chen et al., Embo J (2014) 33:2492

d.- Tiwari et al., J Cell Sci (2013) 136:1962

e.- Hartley et al., Hum Mol Genet (2013) 22:5262

f.- Rosenbaun et al., Genes Dev (2014) 28:1165

g.- Shrimali et al., J Biol Chem (2008) 283:20181

Of note, a significant proportion of the present signatures exhibited a profile similar to the differentially-regulated genes compiled in the GenomicScape resource for naïve and memory BC (NB and MB, respectively) and BMPC [20]. Thus, Fig 2E shows that the expression of PC-down genes was significantly lower in BMPC compared to NB and MB, whereas PC-up genes followed the inverse pattern. This result validated the present human PC signatures.

### The human PC signature is conserved in mouse

In a recent paper, a similar transcriptomic analysis was performed in BC and *in vivo*-generated PC isolated from mouse [30]. We asked whether the transcriptional program of PC maturation was conserved in the two species. In mouse Corcoran’s team identified a transcriptional signature for the so-called ASC, corresponding to the genes that become over-expressed in mouse splenic plasmablasts (SplPB), splenic PC (SplPC) and BMPC compared to mouse BC, i.e. follicular (FoB), marginal zone (MZB) and germinal center B (GCB) cells. These cell-subsets are comparable respectively to human BPC, TPC, BMPC and BC used in the present study. The distribution of the mouse ASC signature was examined across the whole transcriptome of human BC (arranged according to the results of the pair-wise comparisons, see Materials and Methods for further details) and was found to be significant from random distribution in the up-regulated genes in PC compared to BC (*P*< 0.0001, χ^2^ = 633.4 (BC *vs* BPC), 817.6 (BC *vs* TPC) and 802.6 (BC *vs* BMPC), d.f. = 41) (Fig 3A). In this distribution, the most up-regulated genes in human PC contained a large proportion of the mouse ASC signature: 82 genes that were common to the three pair-wise comparisons (top-2000 genes), of which 71 were significant in any comparison (adjusted *P*-value < 0.1) and 44 were present in the PC-up signature (S3 Table). Independently of the list of genes used for functional analysis, we retrieved the categories already outlined in Fig 2D for the entire PC-up signature: protein transport and catabolism, ER stress and unfolded protein response components, regulation of apoptosis, and redox homeostasis (Fig 3B and S2 and S3 Tables). These results support the view that those functions and genes are the most conserved in the PC from the two species, and have been previously reported to be essential in PC biology [1,42–44].

### Comparative analysis of transcriptomes from different human *in vivo*-generated PC

We next compared the three human *in vivo*-generated PC subsets under study to determine the specific transcriptional program for each PC subtype (S4 Table). In this case, the BPC profile contained a significant number of relatively abundant transcripts differentially expressed when compared to TPC and BMPC. In contrast, these two latter PC subtypes showed considerable similarity and a strikingly small number of differentially expressed genes (indicated as “Specific” in Fig 4A). For this reason, we also considered as part of the subtype signatures those up-regulated genes shared by any two PC subsets (indicated as “Common” in Fig 4A). The performance of qPCR in independent human PC samples corroborated the differential expression detected for some selected genes: *E2F2* were highly expressed in BPC compared to TPC and BMPC, whereas the expression of *FOSB* and *EGR1* in these latter cells was higher than in BPC (Fig 4B). Fig 4C shows the GO analysis of BPC-up genes that indicates the predominance of GO terms related with cell division (mitosis, DNA replication and spindle organization), in agreement with the proliferating nature of these cells (see Discussion and [23]). Finally, the expression pattern of the BPC-up signature can be reproduced to some extent in human *in vitro-*generated PC: pre-plasmablasts (prePB), plasmablasts (PB) and early plasma cells (EPC), which represent successive stages of PC differentiation according to the GenomicScape resource [20]. Thus, the BPC-up signature showed a decay in expression from the most immature and proliferating (prePB) to the most mature and resting cell subtype (EPC) included in the analysis (Fig 4D).

### Defining the putative regulatory mechanisms of the transcriptional program of human *in vivo*-generated PC

**Fig 5:**
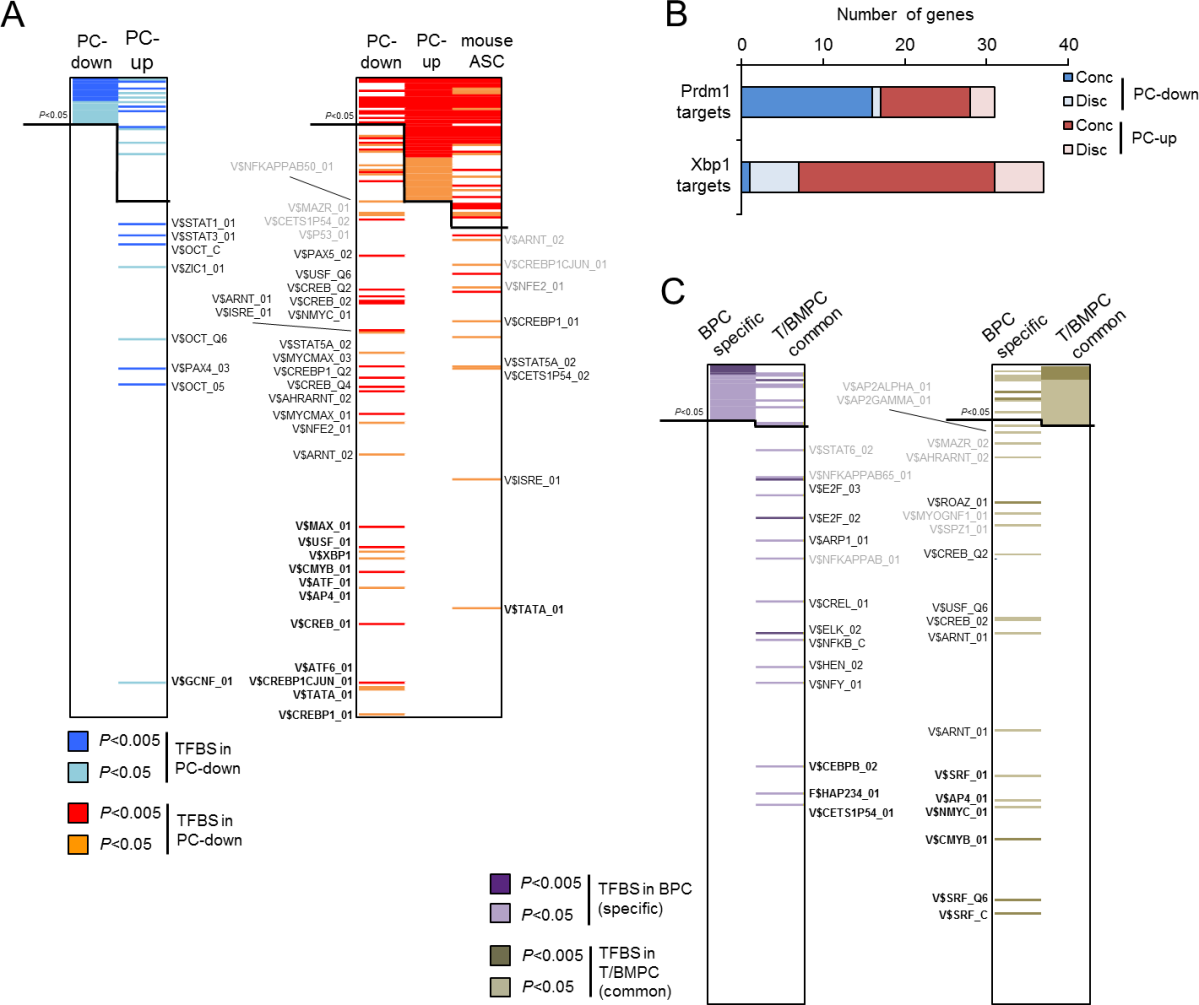
Putative regulation of the PC transcriptional program. **(A)** The 282 TFBS of the TRANSFAC database contained in the Pscan tool are ranked according to their Z-scores in the analysis of PC-down and PC-up signatures. The black lines separate the significantly and non-significantly enriched TFBS (*P* < 0.05). Coloring notes the same TFBS in adjacent ranks: blue and red for significant motifs in the PC-down and PC-up list, respectively. Divergent motifs (significant in one rank but non-significant in the other) are indicated. Letter intensity denotes the degree of divergence, according to a delta (Δ) calculation of the rank position between both lists: bold for Δ > 150, normal for 50 < Δ < 150 and grey for Δ < 50. **(B)** Number of genes in the PC-down (blue) and PC-up (red) signatures that are altered in conditional knockout BMPC for *Prdm1* and *Xbp1* [40]. The genes are divided in concordant and discordant, dependent on the expected direction of change. **(C)** The same analysis as in A was performed for specific BPC and TPC/BMPC genes. Most of the resulting TFBS can be redundant due to sequence similarities, and can be grouped by transcription factor families: AP-2: V$AP2ALPHA_01, V$AP2GAMMA_01; Ahr and Arnt-like (PAS domain): V$AHRARNT_02, V$ARNT_01, V$ARNT_02; ATF/CREB: V$ATF_01, V$ATF6_01, V$CREB_01, V$CREB_02, V$CREB_Q2, V$CREB_Q4, V$CREBP1_01, V$CREBP1_Q2, V$CREBP1CJUN_01; other ATF/CREB related: V$AP4_01, XBP1_01; E-box (bHLH-ZIP): V$MAX_01, V$MYCMAX_01, V$MYCMAX_03, V$NMYC_01, V$SPZ1_01, V$USF_01, V$USF_Q6; E2F-related (Forkhead): V$E2F_02, V$E2F_03; ETS-related: V$ELK1_02, V$CETS1P54_01; NF-κB-related: V$CREL_01, V$NFKB_C, V$NFKAPPAB_01, V$NFKAPPAB50_01, V$NFKAPPABP65_01; NF-Y (CCAAT-binding): F$HAP234_01, V$NFY_01; Oct-like (POU domain): V$OCT_C, V$OCT_05, V$OCT_Q6; PAX: V$PAX4:_03, V$PAX5_02; SRF (MADS-box): V$SRF_C, V$SRF_01, V$SRF_Q6; STAT: V$STAT1_01, V$STAT3_01, V$STAT5A_02, V$STAT6_02.

Accumulated evidence indicates the involvement of a complex transcriptional regulatory network for the generation of PC [1]. To infer whether the transcriptional program of the PC maturation was associated with the activity of specific transcription factors, we scanned the promoters of the genes contained in the present PC signatures for DNA binding motifs of the TRANSFAC database. Comparison of the predictions in PC-down and PC-up genes revealed a poor correlation (Spearman correlation = 0.36) and a limited overlapping of significantly enriched TFBS (19.4% in common to both signatures), suggesting that the potential regulatory mechanisms in these distinctive signatures were largely divergent. Focusing on the differential predictions, we observed a clear enrichment of binding sites for GCNF (Germ Cell Nuclear Factor)/NR6A1 and OCT/POU factors in PC-down but not in PC-up signature (Fig 5A), suggesting that these factors (or other structurally related) were relevant to maintain the PC-down signature but no longer necessary during PC differentiation. Alternatively, these DNA motifs might be indicative of the repressive actions over the PC-down signature. In the case of PC-up genes, their regulatory regions were enriched for XBP1-binding sites, the highly similar activating transcription factor / cAMP responsive elements (ATF/CRE), E-boxes (for the binding of bHLH factors such as USF, MYC and MAX), c-MYB motifs and the aryl hydrocarbon response (AHR) elements for the heterodimer AHR/ARNT (Fig 5A).

Interestingly, analysis of the murine promoters of the ASC up-regulated genes predicted a TFBS pattern similar to that of human PC-up signature (Spearman correlation = 0.71, Fig 5A), indicating an evolutionary conservation at the level of transcriptional regulation. Taking profit of this finding, we conducted a complementary approach using experimental data from genetically manipulated mouse BMPC. Selective inactivation of *Prdm1* and *Xbp1* in conditional knockouts led to the identification of both direct and indirect targets [40] that can be used to estimate the contribution of their orthologs in the human signatures. Fig 5B shows the number of genes that can be potentially regulated by *PRDM1* and *XBP1* in humans, denoted as concordant changes, i.e. the changes that were in the expected direction. Whereas *XBP1* behaved as a clear transcriptional activator in ~12% of the PC-up signature, *PRDM1* targets were spread across down- and up-regulated genes, showing a limited overlap.

The differentially expressed genes between PC subtypes also produced distinctive TFBS predictions: E2F, the CCAAT-binding factor NF-Y, C/EBPβ, ETS factors and NF-κB sites were specific for BPC specific genes, and SRF, CREB, ARNT and E-box-related factors for TPC and BMPC common genes (Fig 5C). This latter profile resembled the obtained with the PC-up signature, reinforcing the potential relevance of these TFBS in the process of PC maturation.

However, no clear patterns were obtained for certain transcription factors that are differentially expressed during the BC maturational program. For instance, PAX binding motifs were significant across all the PC signatures. This was not surprising considering the detection of several members of the family in the pair-wise comparisons: *PAX4* and *PAX5* in naïve BC, *PAX8* in all the PC and *PAX2* in BPC. The same occurred for the activator EGR1, whose expression was higher in mature PC compared to BPC; in this case, the concurrence of GC-rich promoters in the analysis precluded a proper discrimination of EGR/Krox responsive elements. For the AP-1 member FOSB, we cannot discard its potential binding to CREs due to sequence similarity with AP-1 binding sites or through dimerization with ATF members (see Discussion). Despite these caveats, this *in silico* analysis provided novel hypothesis for further identification of transcriptional regulators and their targets during PC differentiation.

### Potential involvement of the present PC signatures in multiple myeloma

**Fig 6:**
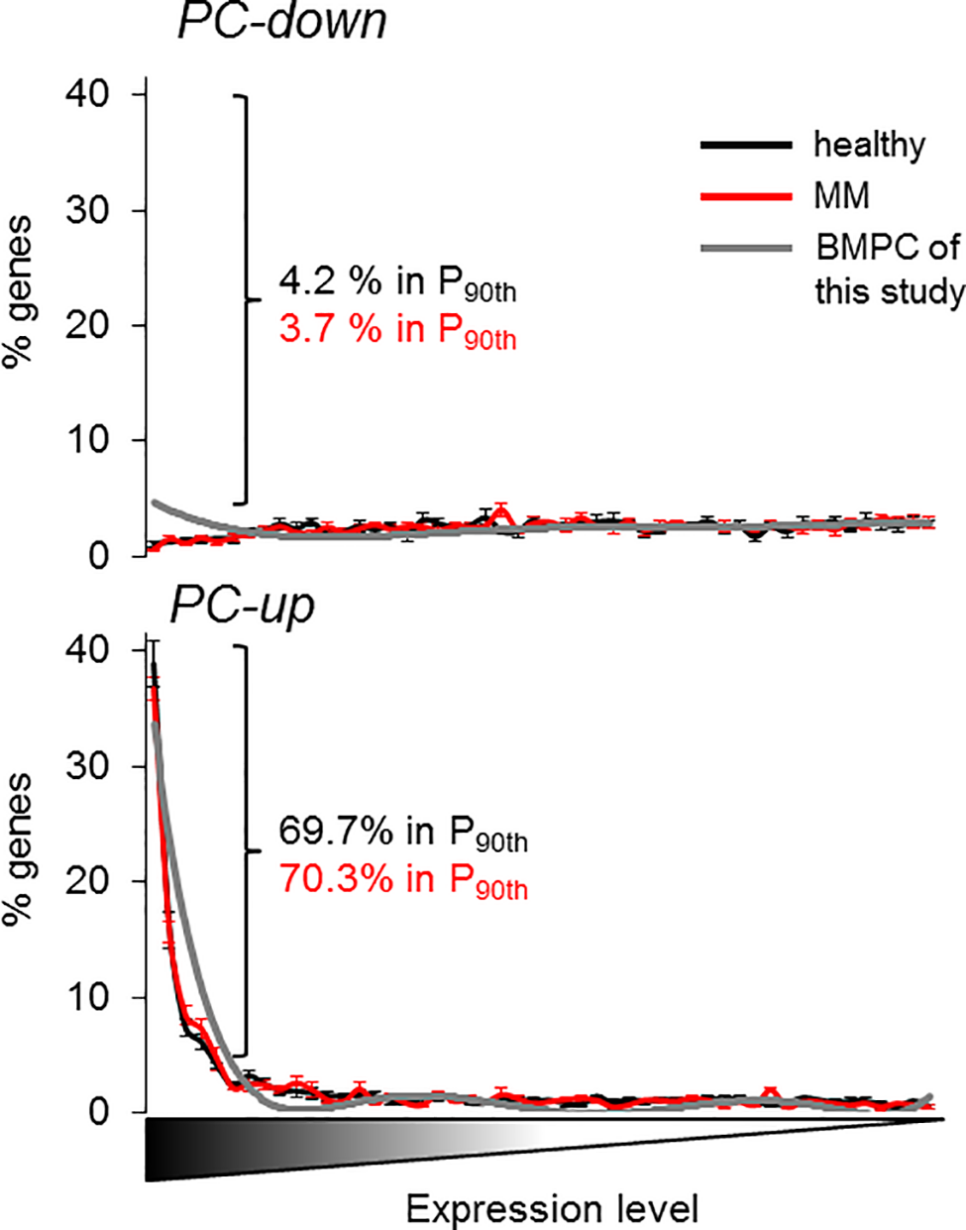
Transcriptomic distribution of PC signatures in healthy BMPC and MM. The whole transcriptomes of BMPC and MM from four datasets (see Materials and Methods) were ranked according to the level of gene expression, and the number of the genes contained in the PC-down (upper panel) and PC-up (lower panel) signatures were counted and averaged in each bin. The profile of our in-house BMPC is depicted in gray for comparative purposes. Data are expressed as mean ± SD. The % of genes in the 90^th^ percentile is also depicted.

**Fig 7:**
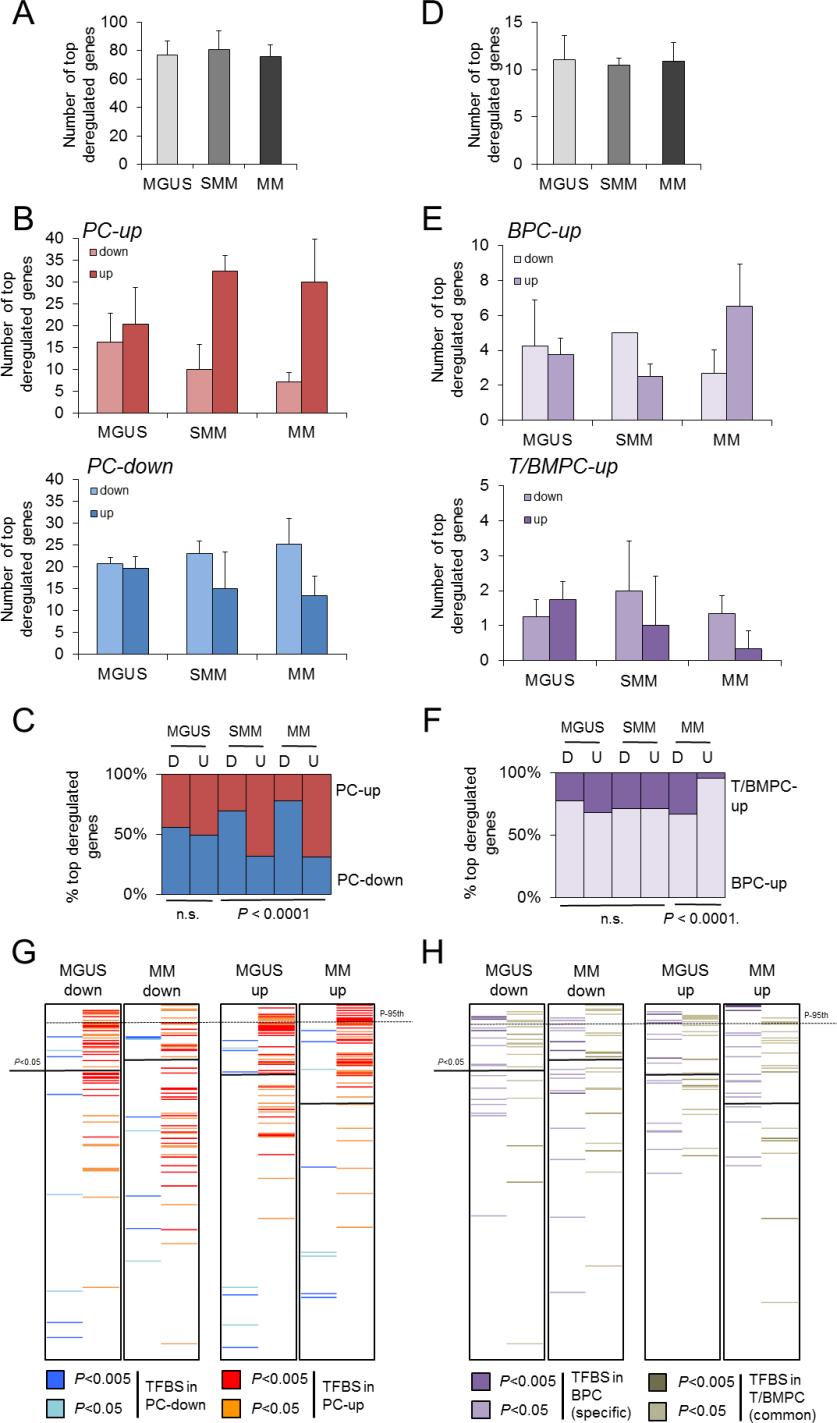
The PC transcriptional program is imbalanced in monoclonal gammopathies. Genes of the PC-down and PC-up signatures that were also dysregulated genes in MGUS, SMM and MM related to healthy BMPC are represented as total number **(A)** or as number and % in each signature and direction of change **(B, C)**. The same representation is depicted for BPC-up and TPC/BMPC-up signatures **(D-F)**. Data are expressed as mean ± SD. (A, B, D, E) or as proportion (C, F). **(G, H)** Prediction analysis of TFBS enrichment in the dysregulated genes in MM and MGUS. Similar to Fig 5, The TFBS of the TRANSFAC database in the Pscan tool are ranked according to their Z-scores in the analysis of PC-down and PC-up signatures (G) and BPC-up and TPC/BMPC-up signatures (H). The black lines separate the significantly and non-significantly enriched TFBS (*P* < 0.05). Coloring follows the same code as in Fig 5. The 95^th^ percentile (the most significantly enriched TFBS) is shown in a dash line for comparison purposes.

Once we established the transcriptional program of B-cell maturation into different PC subtypes and proposed its putative regulatory mechanisms in physiological conditions, we wanted to know the relevance of these findings in pathology. To this aim, we analyzed the relationship of the present PC signatures with the best studied disorder linked to PC malfunctioning, the monoclonal gammopathies, namely the MM, for which extensive information has been compiled in public datasets repositories in the last years. MM accounts for 0.8% of new cancer cases and 0.9% of cancer death worldwide [45] and is defined by malignant transformation of PC that usually overproduce large quantities of an aberrant monoclonal immunoglobulin fragment, the M protein. Early work demonstrated that MM transformation did not promote a general dedifferentiated state related to mature healthy BMPC [18]. To confirm this conclusion, we compared the gene expression profile of the present PC signatures with that of purified CD138^+^ BM cells from healthy and patient donors, considering in-house and several sources of external datasets as we were interested in consistent and reproducible results (see Materials and Methods for further details). Interestingly, the overall expression of the PC-up genes in any BMPC transcriptome was increased to the levels of highly-expressed genes such as the housekeeping *GAPDH* and *β-ACTIN* (Fig 6). The distribution of the PC-up signature across the transcriptomes of both healthy and malignant cells were substantially the same (*P* = 1, χ^2^ = 22.1, d.f. = 49) and approximately 70% of the PC-up genes were highly expressed in all the profiles (90^th^ percentile, Fig 6). Based on this observation, we pursued more specific changes in the PC signatures. In a first approach we examined the differential expression of distinct progressive stages of malignancy compared to healthy BMPC: the silent monoclonal gammopathy of undetermined significance (MGUS), the precancerous and asymptomatic smoldering MM (SMM) and the malignant MM. To obtain again consistent results, datasets from various studies were used. Because of in different experimental conditions between laboratories (microarray platforms, number of samples, procedures) that may impose a strong bias in the analysis for certain datasets in detrimental of others, only the most dysregulated genes were examined (see Materials and Methods for further details). Overall, the three pathologic PC variants showed a similar number of dysregulated genes that were contained in the present PC-down and PC-up signatures (Fig 7A). However, a further dissection of these genes revealed a progressive pattern of imbalance that correlated with malignancy: whereas in MGUS the transcriptional dysregulation was similar in both directions of change, in MM differences between downregulation and upregulation were exacerbated. Thus, dysregulated genes present in the PC-down and PC-up signatures were even more downregulated and upregulated, respectively (Fig 7B and 7C). In agreement with this observation, SMM represented an intermediate situation between MGUS and MM (Fig 7B and 7C). In a similar way, the BPC-up and the TPC/BMPC-up signatures showed this imbalance, although restricted to upregulation (Fig 7D–7F). The proposed genes that explained the imbalance are shown in Table 2, and mainly consisted of genes involved in leukocyte/BC related functions and positive regulation of apoptosis (PC-down) and ribosome and translation (PC-up) (*P*<0.05, DAVID). The upregulation of BPC-up genes in MM may confer some of the BPC features to malignant cells (Table 2 and Discussion). According to this view, we observed a downregulation of the immunoglobulin related genes in monoclonal gammopathy compared to healthy cells, reminiscent to their lower expression in normal BPC compared to BMPC (S2A Fig).

**Table 2:** Differentially expressed genes of the PC signatures between MM and MGUS.

*PC-down*		*PC-up*		*BPC-up*		
**Down-regulated in MM**		**Up-regulated in MM**		**Up-regulated in MM**		
*Primary*	*Secondary*	*Primary*	*Secondary*	*Primary*		*Secondary*
ADRBK2	ABCG1	CDK9	ALDOA	PSMA2		MTCH2
ARHGAP25	ATM	NME1	BST2	RAN		
CR1	HLA-DMB	RPL12	EDF1	TUBB		
HLA-DBQ1	LYST	RPL18A	EIF3E			
IL4R	MARCH1	RPL24	HIST1H2BD			
INPP5D	PRMT2	RPL27A	P4HB			
KIAA0922	ZNF254	RPL35	RPL3			
PDE4DIP		RPLP0	SLC1A5			
WNK1		RPS15	UBA5			
		RPS16				
		RPS28				
		RPS9				
		SND1				
		SRPRB				
**Up-regulated in MGUS**		**Down-regulated in MGUS**				
*Primary*	*Secondary*	*Primary*	*Secondary*			
	CAPN6		ACADVL			
	GPR132		HSP90B1			
	KLHL18		XBP1			
	PIK3CD					
	SCML1					
	SMAD3					

The table shows the genes that are candidates to explain the differences observed in Fig 7: only those genes appearing in at least two studies were considered (see Materials and Methods). Genes are classified according to their PC signature and the criterion of selection: *Primary*, top1000 genes in both pair-wise comparisons (gammopathy *vs* healthy, and MM *vs* MGUS); *Secondary*, top1000 genes in the comparison gammopathy *vs* healthy, based on the less stringent presence/absence criterion in MM or MGUS. No genes were retrieved in the TPC/BMPC-up signature.

Interestingly, the transcriptional mechanisms that were predicted to regulate the PC-signatures (Fig 5), when applied to the study of all changing genes in MM and MGUS, followed a distribution pattern that resembled the gene expression imbalance (Fig 7G and 7H). To keep in mind, the altered genes from the PC signatures represented only the 6–7% of the total number of genes used in the prediction (denoted as MM-up, MM-down, MGUS-up, MGUS-down), therefore, the results would indicate that the putative transcriptional regulation of the affected genes in gammopathies were highly similar to those found in the PC signature regulation. In other words, this resemblance reflected the involvement of common regulatory mechanisms between PC differentiation and MM pathology that remains to be further explored. More specifically, TFBS associated with PC-up and BPC-up were among the first ranked significant motifs in upregulated genes in MM compared to MGUS, whereas the same TFBS exhibited a more disperse pattern in the downregulated genes. In contrast, the PC-associated TFBS were equally distributed in upregulation and downregulation in MGUS (Fig 7G).

In the last years, several efforts have been focused on the identification of genes and the design of algorithms based on gene expression for the stratification of MM patients in terms of survival. In this context, we asked whether the genes contained in the present PC signatures may contribute to additional markers of MM prognosis risk. Here we present a proof-of-principle about the potential use of these signatures in future studies. Using three different datasets of MM, we found a consistent subset of genes that were correlated with low and high risk (Table 3), according to the GEP70-based score [36], that may deserve further research (see Discussion). As a validation of this approach, we found genes that have been used to calculate the proliferative index in MM [46,47]; interestingly, these genes were specifically associated with the BPC-up signature.

**Table 3:** Genes of the PC signatures as potential MM risk markers.

***Low risk***	N = 52	N = 126	N = 156
PC-down: DRAM1	-0.38	-0.26	-0.43
PC-down: WDFY2	-0.37	-0.26	-0.47
PC-down: ZNF331	-0.53	-0.23	-0.25
PC-up: DNAJC3	-0.28	-0.25	-0.28
PC-up: POU2AF1	-0.30	-0.20	-0.25
PC-up: SYVN1	-0.42	-0.28	-0.21
PC-up: TRIB1	-0.28	-0.23	-0.21
TPC/BMPC-up: EGR1	-0.32	-0.20	-0.28
***High risk***	N = 52	N = 126	N = 156
PC-down: CPSF6	0.36	0.49	0.16
PC-down: DESI2	0.33	0.26	0.23
PC-down: PRMT5	0.28	0.23	0.40
PC-up: NME1**	0.32	0.35	0.42
BPC-up: BIRC5*	0.64	0.51	0.53
BPC-up: DHFR	0.35	0.24	0.31
BPC-up: E2F2*	0.70	0.34	0.34
BPC-up: GMNN*	0.50	0.33	0.34
BPC-up: KIFC1	0.59	0.26	0.34
BPC-up: PCNA*	0.40	0.27	0.41
BPC-up: RAN**	0.41	0.40	0.35
BPC-up: RRM2*	0.72	0.45	0.40
BPC-up: SHCBP1	0.49	0.34	0.35
BPC-up: SPC25*	0.61	0.50	0.40
BPC-up: TSEN15	0.52	0.40	0.40
BPC-up: UQCR10	0.35	0.21	0.34
BPC-up: ZWINT*	0.49	0.42	0.46

The table indicates the Pearson correlation coefficient and the number of samples of each study.

*, present in proliferative indexes

**, differentially expressed between MM and MGUS (Table 2).

## Discussion

The main objective of the present study was to delineate the signatures of accessible human *in vivo*-generated and functionally-different PC subsets. By using the transcriptome of BC as reference, the coincidental mRNA changes observed in the comparison with the transcriptomes of three *in vivo*-generated PC subsets (early TPC, BPC and mature BMPC) reflect a consistent human PC signature (Fig 2C and 2D). These signatures were later used to explore their potential involvement in monoclonal gammopathies.

### Human PC down-regulated gene signature

The analysis of the PC-down genes (Fig 2C, Table 1 and S1 and S2 Tables) reveals that many of them are associated with several BC developmental and functional programs. These observations are consistent with the “switching off” of a variety of BC activities, which are no longer required in the PC stage. In fact, the persistent expression of some of these genes (*PAX5*, *FOXP1*) inhibits progression to the PC stage [48,49]. Nevertheless, certain B cell functions appear to remain present in some PC subsets, as will be discussed later.

A second group of PC-down genes corresponds to transcriptional regulators and transcription factors that have not been previously implicated in the functioning of BC, comprising several Zn-finger factors (Table 1). It is also notable that two histone deacetylase (*HDAC4*, *HDAC10*), the suppressor of EGR molecules *NAB2*, and *SMAD3* are also down-regulated; the suppression of this latter molecule has been previously demonstrated at both the transcriptomic and epigenomic levels in the transition of human BC to PC in an *in vitro* system [50].

Despite not being significantly enriched in the GO analysis, the present data also show that genes of an activator of the adrenergic pathway and a neurotransmitter receptor (*ADRBK2* and *GABBR1*, respectively), as well as genes of endogenous cannabinoid receptors (*CNR2*, *GPR18*) are reduced during the BC to PC transition. This is of particular relevance in the case of CNR2, a receptor clearly expressed and functionally active in human BC [51]. These findings suggest that PC are less susceptible than BC to neuromodulation.

At the regulatory level (Fig 5A), the PC-down signature appears to be potentially controlled by OCT/POU factors, an observation connected with the positive inductive role that they exert on the development of BC, namely B1 and MZB subtypes [1]. Nevertheless, this observation is intriguing as Oct-2 and its co-activator Obf-1, which are primarily expressed in B-cell lineage, are relevant for differentiation of ASC as part of the T cell-dependent responses [52–54]. This is in agreement with the presence of *POU2AF1*, the gene encoding OBF-1, in the human PC-up signature (S1 Table). Two possible alternatives may explain this apparent paradox: these factors can also mediate repression [55] or OCT/POU binding motifs are recognized by a structurally related factor in naïve BC that is eventually repressed during PC maturation.

### Human PC up-regulated gene signature

In contrast to PC-down genes, the GO analysis of the PC-up signature (Fig 2D, Table 1, S1 and S2 Tables) reveals an enrichment of functional terms associated with protein processing (Table 1). All these functions are linked to the massive and permanent Ab production occurring at the PC stage [42–44]. Interestingly, similar results were obtained with the overlapping genes between the human PC-up and the mouse ASC signatures (Fig 3B and S3 Table), and confirmed that a significant proportion of the molecular components that control PC maturation in human and mice is conserved in the two species. This assumption is further emphasized by the similarities observed in the analysis of putative transcriptional regulation in PC from both species (Fig 5A and 5B).

A significant number of PC-up genes are also associated to the control of apoptosis. These include *MCL1*, the well-established and specific pro-survival element operative in long-lived PC in mice [56], a factor apparently modulated by the intrinsic expression of the TF *Zbtb20* [57]. Besides *MCL1*, other anti-apoptotic genes are also up-regulated (Table 1). This observation suggests that the PC stage is endowed with several complementary and/or alternative survival pathways, an assumption also supported by the *Zbtb20*-deficient mouse model, in which PC continue to survive in the absence of *Mcl1* up-regulation, after induction under Toll Like Receptor-activating adjuvants [57]. Regulation of apoptosis is also conserved between human and mouse (Fig 3), despite the fact that *Mcl1* is not retrieved in the mouse ASC signature [30].

Another unexpected finding that deserves further exploration was the detection of up-regulated genes for histones H2A and H2B, and genes involved in DNA–macromolecular complex formation (Table 1) contained within the “Chromatin assembly” category (Fig 2D), that suggest a general chromatin reorganization during PC differentiation. This is intriguingly reminiscent of histone biogenesis and remodeling of the chromatin architecture during cell cycle [58].

The role of XBP1 in the definition of the PC-up signature is highlighted by its strong up-regulation concomitant to the detection of both reported and predicted targets (Fig 5). In the prediction, XBP1 and CREB binding motifs are overlapping due to their sequence similarity. Whether these sites are exclusively utilized by XBP1 or in conjunction with an ATF/CREB factor remains to be explored. Of note, CREB regulates the expression of *MCL1* in B-cells [59] and, possibly its actions can be extended to other components of the PC gene expression program. In addition, *MCL1* can be also regulated by ATF5 [60], a factor that is up-regulated in human and murine PC (S3 Table). Interestingly, the ligand-activated factor AHR, also predicted to be an important regulator, can potentially mediate PC maturation upon external stimuli [61,62], a possibility that may open the avenue to explore both immunosuppression and B cell malignancies induced by exogenous AHR ligands.

### Profiling of human PC subtypes

A previous work demonstrated that human PC released to the circulation six days after a potent booster immunization (the BPC of the present study) contained a substantial number of Ag-induced proliferating cells, i.e., plasmablasts [23]. Therefore, as expected, the GO analysis of these comparisons reveals that human BPC exhibit a specific and consistent up-regulation of 55 genes (Fig 4A; BPC-up signature), most of which can be associated with cell division (Fig 4C and S2 and S4 Tables). Interestingly, BPC-up signature is also partially shared by *in vitro*-generated PB (Fig 4D) [20]. The functional role of the proliferating activity present in BPC has been discussed elsewhere [23,50].

Previous studies have demonstrated that BPC are derived from the recent Ag-activation of memory BC, since they contain *IGVH* genes that are as highly mutated as those of BMPC [15,63]. In addition, BPC are thought to be a transitional cellular phase prone to undergo rapid apoptosis unless they reach the limited number of PC-survival niches present in BM, mucosa lamina propria, and inflammatory and reactive tissues including certain locations in SLO [14,23]. Therefore, it has been proposed that BPC include the circulating precursors of long-lived PC, which would rapidly develop the final PC maturational program upon their migration into the survival niche [14,23,64], an assumption also supported by the demonstrated inductive effect of PC-niche cytokines on purified BPC in culture [16,23]. This could explain the relative immaturity exhibited by these cells with respect to TPC and BMPC, an immaturity which is potentially transient. This immaturity is reflected in the comparison with the *in vitro* PC differentiation stages in Fig 4D.

On the other hand, TPC and BMPC share a group of 14 up-regulated genes that conversely are down-regulated in BPC (Fig 4 and S4 Table). These genes do not confer any significant functional enrichment, although several transcription factors were observed. The known role of some of these genes may explain the functional diversity occurring among these PC subsets, including improved ER stress response (*EROILB*) and increased resistance to apoptosis (*ZNF667*, *FOSB*, *EGR1*) by TPC and BMPC with respect to BPC. Of particular interest, EGR1 has been demonstrated to participate directly in the induction of the PC master regulator *PRDM1* in humans [65]. Again, these results suggest that there is a certain degree of immaturity in BPC in comparison with TPC and BMPC.

As mentioned above, TPC and BMPC appear to show a small number of gene profiling differences (Fig 4A and S4 Table). The human TPC preparations used in the present study mainly consist of early short-lived PC recently generated in the extra-follicular Ag-activated foci and GC, as revealed by their high tendency to undergo apoptosis and low number of mutations in their *IGVH* genes [10,11,15]. In contrast, it is generally assumed that BMPC are finally-differentiated PC [13–16]. However, and despite their different physiological roles, both subsets share very similar transcriptomes. A recent report has demonstrated the complex composition of the human BMPC compartment, even within the highly-mature CD138^+^ BMPC subsets used in the present study [17], revealing a heterogeneity that may diminish the statistical power in the differential expression analysis between TPC and BMPC. Alternatively, both PC subtypes might be in a maturational stage closer than anticipated. Nevertheless, TPC still retain several BC features, as indicated by the up-regulation of genes such as *CD22* (S4 Table), plus a trend towards elevated transcript levels of *MS4A1* (CD20) (S1 Table), an observation corroborated at the protein level [13]. This observation is in agreement with the notion that TPC mainly consist of early short-lived PC [10,11,13–15], a PC phase less mature than BMPC. In addition, TPC as well as EPC from other human SLOs express *IGVH* genes containing significantly fewer mutations than those present in BPC and BMPC [15]. These observations suggest that, in human SLOs, those PC producing Ab that have acquired high Ag-affinity receive the correct signals that confer migratory capacity, and eventually home into survival niches.

It is well-established that *CIITA* is critical for the expression of HLA-DR molecules and that the former gene is repressed by *PRDM1* early during the development of the PC program [1,14], which leads to the loss of MHC class II expression in mature PC. Despite the observed down-regulation of *CIITA* in the three human PC subsets under study (S2 Fig and S1 Table), TPC and BPC exhibit substantial expression of MHC class II molecules clearly greater than BMPC (S4 Table), a fact that has also been observed at the protein level for surface HLA-DR [13]. The mechanism of the persistent expression of HLA-DR molecules in some human PC is unknown. In addition, BPC exhibit a high expression of CD86 at both RNA and protein levels (S4 Table and [66]). The expression of HLA-DR and CD86 are two hallmarks of Ag-presenting cells, suggesting the possibility that BPC can perform this function. This finding is consistent with the reported functional interaction of BPC with certain T lymphocytes [23], a process that might occur *in vivo* in reactive and inflammatory areas, where T lymphocytes have been shown to contribute to the PC-survival niches. This assumption is also supported by the observation that BPC express high levels of *SELL* (S4 Table) and of its product CD62L [13], a selectin that allows the PC to home to reactive and inflamed areas.

Production of APRIL in the PC-survival niches is essential for the maintenance of long-lived PC in mice and humans [1,14,16,67], and its effect has been shown to be exerted through *Tnfrsf17* in mice [67]. Although *TNFRSF17* is up-regulated in the three human PC subsets (S2 Fig and S1 Table), its expression is relatively lower in human CD138^+^ BMPC (which contain the long-lived PC compartment) with respect to TPC and BPC (S4 Table). This finding has also been confirmed at the protein level [16]. In addition, human BPC and BMPC express *TNFRSF13B* (S4 Table), the other receptor for APRIL, at higher levels than TPC, an observation also corroborated at the protein level [16]. In consequence, it may be hypothesized that *TNFRSF13B* has a role in the APRIL-dependent survival of long-lived PC in humans.

The only gene specifically up-regulated in BMPC is *ATP12A*. Its product, an H^+^/K^+^ ATPase with proton pump activity, has been demonstrated recently to act as an anti-apoptotic factor in human myelomonocytic cells [68]. Whether or not this is the function of ATP12A in human BMPC remains to be proven.

Finally, the TFBS prediction in the genes defining the PC subtypes seems to reflect the relevance of cell cycle regulation, in concordance with the higher levels of *E2F2* in BPC compared to more mature PC (Fig 4B and S4 Table). The signature of these latter cells shows a depletion of cell cycling-related TFBS such as E2F and NF-Y. E2F is also active in earlier stages of B-cell development [69] and NF-Y is important for cell cycle and apoptosis in other cellular systems [70]. Meanwhile, TPC and BMPC specific genes retrieved similar TFBS as in the PC-up signature, with the addition of SRF which plays a role in the retention of hematopoietic progenitor cells in the BM [71]; whether this factor plays a similar role in human mature PC remains to be determined.

### Potential involvement of the normal PC signatures in monoclonal gammopathies

Comparison of the defined PC signatures with the altered gene expression in monoclonal gammopathies reveals that the PC transcriptional program is not generally disrupted in tumoral PC (Fig 6 and [18]). Therefore, it is not surprising to observe that the number of altered genes included in the normal PC-signatures was low and similar between different stages of malignancy (Fig 7A and 7D). However, we found a significant transcriptional imbalance in malignant condition, i.e., the transcriptional regulation of PC differentiation was partially disrupted in gammopathies, leading to the selective perturbation of part of the PC maturational program that become increasingly exacerbated as malignancy progresses, with more downregulated genes of the PC-down signature and more upregulated genes of the PC-up and BPC-up signatures (Fig 7B, 7C, 7E and 7F and Table 2). Even taking into account that our meta-analysis prioritized consistency over quantity of genes (due to the lower number of transcripts contained in the Human Genome U133A array related to more recent array versions), this imbalance was clearly restricted to selective subsets of genes (in total, ~4% of the genes in the PC signatures that were used in the comparative analysis), and correlated with imbalances in the prediction of upstream transcription factors that overlapped with the putative regulatory mechanisms of the PC program (Fig 7G, and 7H). We propose that alterations in the PC signatures might be functionally compensated in the asymptomatic MGUS variant; it is tentative to speculate that this situation may account for a priming state that only acquires net directions of gene expression changes in malignant phases. In MM, such dysregulation did not necessarily follow the regressive model to an immature state, as the altered expression of genes in PC-down and PC-up signatures was even more pronounced in terms of respective downregulation and upregulation, and not vice versa. Instead, present results are in agreement with MM as a separate differentiated state that is characterized, among other alterations, by overexpression of protein biosynthesis genes and further suppression of BC-related genes (Table 2). A straight relationship between protein biosynthesis and proliferation is unclear in MM [31], despite the putative involvement of proliferative-related transcription factors such as E2F (Fig 7H).

Proliferation involvement was more evident in the survival risk analysis for MM (Table 3), with the retrieval of genes previously used for proliferative index calculations [46,47]. These proliferative genes were also present in the BPC signature, which might suggest a special relationship of this subset with MM. Additional genes of interest were those associated with low risk, the apoptotic inducers *DRAM1*, *EGR1* [72] and *ZNF331* [73]. More intriguing is the retrieval of *POU2AF1* and *TRIB1*, previously related with tumorigenesis [74,75]. Although more experimental work is required to validate these genes as prognostic markers, we want to highlight the importance of analyzing as a whole components of relevant cellular processes (for instance, the PC maturation transcriptional program) rather than out-of-context genes to better understand the complexities of expression changes associated with pathology, and to improve the correlation of global molecular patterns with survival outcome. In this sense, a recent work identified a signature based on inflammation as relevant for MM prognosis [76].

### Final conclusion

This report describes a comprehensive analysis of the transcriptional program associated with three human *in vivo*-generated PC subtypes, compared to human naïve B cells. The present study provides new insights into key aspects of PC biology after integrating both novel and existing information of the PC transcriptional signatures, shedding light about putative functions, reproducibility in *in vitro* preparations and evolutionary conservation. We also propose that this transcriptional program is disrupted in monoclonal gammopathies in a selective manner and may provide novel candidates of relevance for stratification of MM patients.

## Supporting information

S1 Fig Extended PCA of human naïve BC and the three human in vivo-generated PC subtypes. (A) Considering all the probesets (n = 33060). (B) Considering the differentially expressed probesets (n = 2397).
(TIF)

S2 Fig Immunoglobulin-related genes and known markers in B-cells and PC. (A) Immunoglobulin (Ig)-related genes are the most up-regulated in PC compared to BC. The genes represented in the Agilent arrays were ranked according to the t statistic for each pair-wise comparison, and Ig-related genes were plotted against the resulting distribution. BPC does not achieve full levels of Ab expression compared to TPC and BMPC. (B) Gene profiling of the Ig-related genes for BC and PC, depicted as normalized expression values for the corresponding probesets in the samples used in the present study. The ‘Ratio’ column is the ratio of average expression of BMCP/BC. Q1, Q2 and Q3 are the quartile thresholds of expression values for the entire microarray dataset. (C), The hierarchical clustering correctly separate BC and PC markers. (D) RT-qPCR assays in independent samples (n = 4 for BC and BMPC, n = 5 for BPC and n = 3 for TPC) for selected genes in PC maturation. *, P ≤ 0.05, Mann-Whitnney tests related to BC. Data are expressed as mean ± s.e.m.
(TIF)

S1 Table PC-down and PC-up signatures. Differentially expressed genes (adjusted P-value < 0.1) in the three pair-wise comparisons between each PC and naïve B-cells. Note that genes were compared (and not probesets) in the Venn diagrams.
(PDF)

S2 Table Gene Ontology analysis. The table shows the significant GO terms (P < 0.05, DAVID) with associated P-values, fold enrichment, genes and the manual categories used in the figures.
(PDF)

S3 Table Common PC genes between human and mouse. See main text and legend of Fig 3 for further details.
(PDF)

S4 Table PC subtypes signatures. Differentially expressed genes (adjusted P-value < 0.1) in the pair-wise comparisons between PC subtypes. Black, specific genes; red, common genes (see main text).
(PDF)

## Abbreviations

Ab: Antibody
Ag: antigen
ASC: antibody secreting cell
B: blood
BC: B-cells
BM: bone marrow
GC: germinal center
GO: Gene Ontology
EPC: early plasma cells
ER: endoplasmic reticulum
IGVH: immunoglobulin variable heavy genes
MB: memory B cells
MGUS: M gammopathy of undetermined significance
MM: multiple myeloma
MZB: marginal zone B cells
NB: naïve B-cells
PB: plasmablast
PC: plasma cells
SMM: smoldering MM
SLO: secondary lymphoid organs
Spl: spleen
T: tonsil
TFBS: transcription factor binding sites
RT-qPCR: real-time quantitative reverse transcribed PCR
