# Supplementary figures and images for: The transcriptional profiling of human in vivo-generated plasma cells identifies selective imbalances in monoclonal gammopathies
Source: PLoS One. 2017 Aug 17;12(8):e0183264. doi: 10.1371/journal.pone.0183264 (PMC5560601; doi:10.1371/journal.pone.0183264)

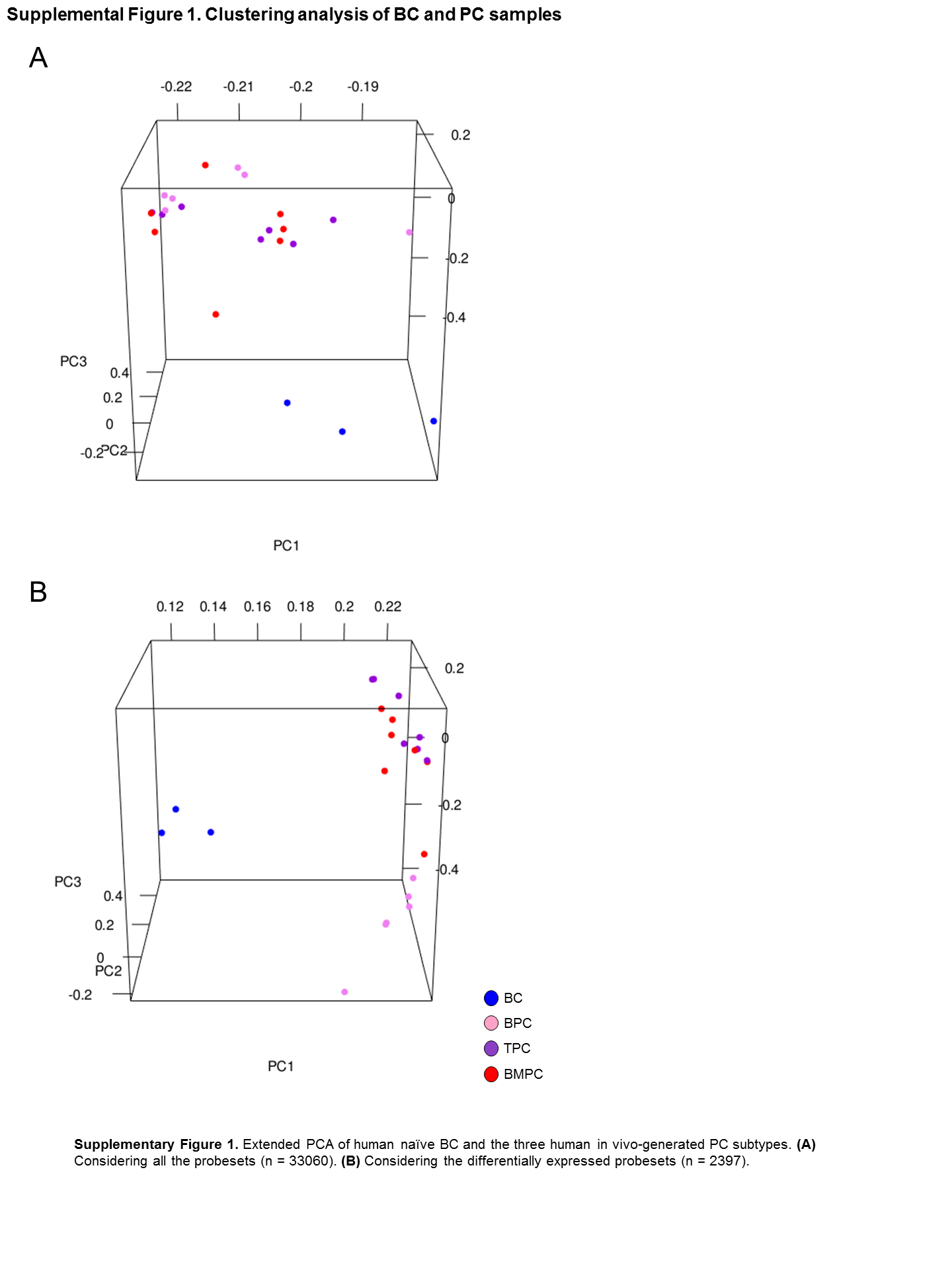

Supplement: S1 Fig — (A) Considering all the probesets (n = 33060). (B) Considering the differentially expressed probesets (n = 2397). (TIF) [file pone.0183264.s001.tif]

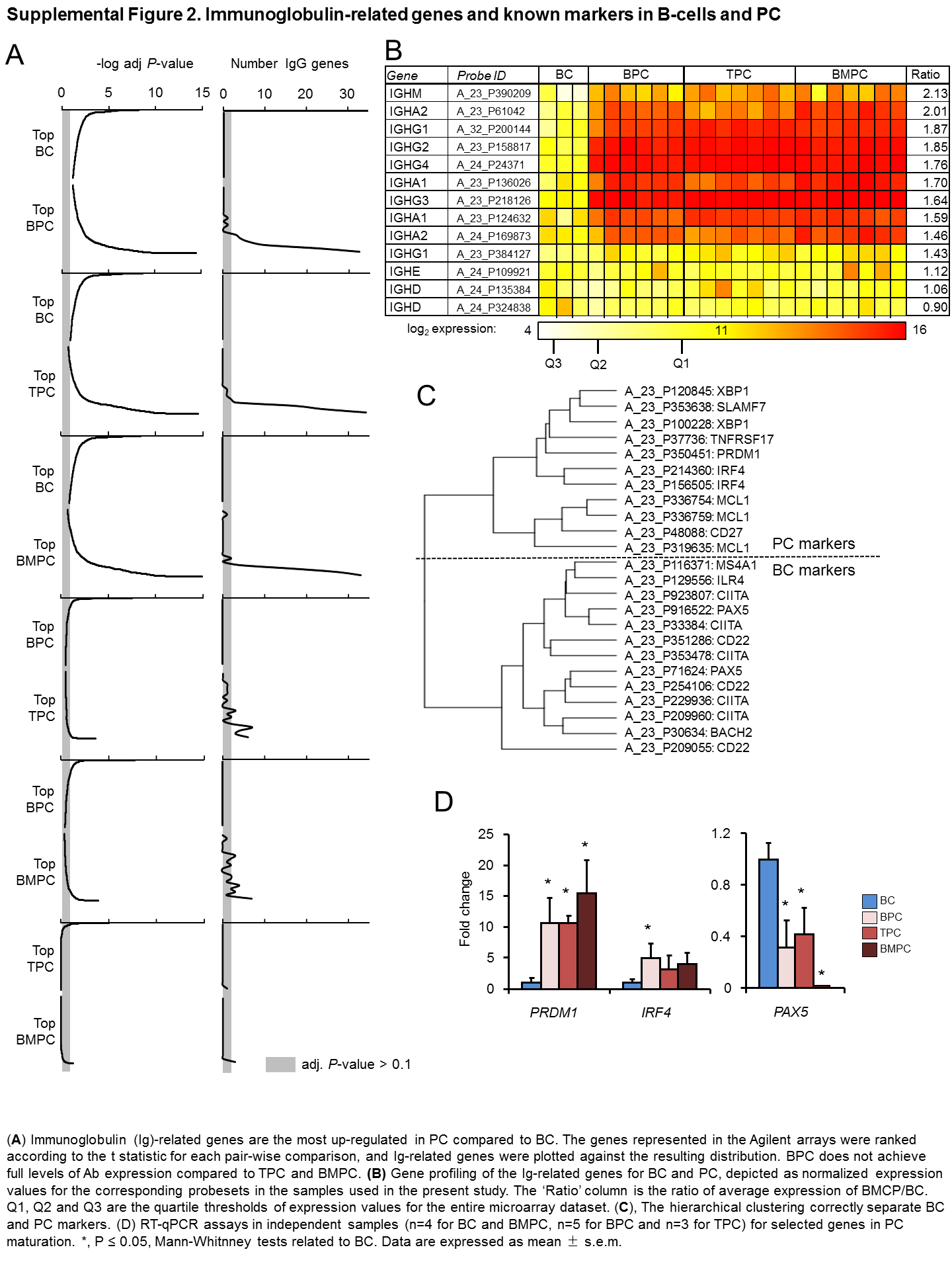

Supplement: S2 Fig — (A) Immunoglobulin (Ig)-related genes are the most up-regulated in PC compared to BC. The genes represented in the Agilent arrays were ranked according to the t statistic for each pair-wise comparison, and Ig-related genes were plotted against the resulting distribution. BPC does not achieve full levels of Ab expression compared to TPC and BMPC. (B) Gene profiling of the Ig-related genes for BC and PC, depicted as normalized expression values for the corresponding probesets in the samples used in the present study. The ‘Ratio’ column is the ratio of average expression of BMCP/BC. Q1, Q2 and Q3 are the quartile thresholds of expression values for the entire microarray dataset. (C), The hierarchical clustering correctly separate BC and PC markers. (D) RT-qPCR assays in independent samples (n = 4 for BC and BMPC, n = 5 for BPC and n = 3 for TPC) for selected genes in PC maturation. *, P ≤ 0.05, Mann-Whitnney tests related to BC. Data are expressed as mean ± s.e.m. (TIF) [file pone.0183264.s002.tif]
