# Supplementary material for: The transcriptional profiling of human in vivo-generated plasma cells identifies selective imbalances in monoclonal gammopathies
Source: PLoS One. 2017 Aug 17;12(8):e0183264. doi: 10.1371/journal.pone.0183264 (PMC5560601; doi:10.1371/journal.pone.0183264)
Supplement: S1 Table — Differentially expressed genes (adjusted P-value < 0.1) in the three pair-wise comparisons between each PC and naïve B-cells. Note that genes were compared (and not probesets) in the Venn diagrams. (PDF) [file pone.0183264.s003.pdf]

Supplementary Table 1. PC-down and PC-up signatures: Differentially expressed genes (adjusted P-value < 0.1) in the three pair-wise comparisons between each PC and naïve B-cells. Note that genes were compared (and not probesets) in the Venn diagrams.

PC-DOWN SIGNATURE

ProbeName	log2FC BC / BPC	adj.P-Val BC / BPC	log2FC BC / TPC	adj.P-Val BC / tpC	log2FC BC / BMPC	adj.P-Val BC / BMPC	Gene Symbol	Gene description	Refseq & others	UNIGENE_ID
A_23_P82523	0,94	0,0674	0,93	0,0932	1,00	0,0628	ABCB1	ATP-binding cassette, sub-family B (MDR/TAP), member 1	NM_000927	Hs.489033
A_23_P74794	2,59	0,0098	3,47	0,0005	3,29	0,0011	ABCD3	ATP-binding cassette, sub-family D (ALD), member 3"	NM_002858	Hs.700576
A_23_P166297	3,33	0,0008	3,76	0,0001	4,01	0,0001	ABCG1	ATP-binding cassette, sub-family G (WHITE), member 1	NM_207627	Hs.124649
A_23_P211207	3,19	0,0437	2,85	0,0975	3,26	0,0481	ADARB1	adenosine deaminase, RNA-specific, B1	NM_001112	Hs.474018
A_23_P361336					4,04	0,0830	ADARB1	adenosine deaminase, RNA-specific, B1 (RED1 homolog rat)	NM_015833	Hs.474018
A_24_P260346	4,29	0,0027	4,73	0,0008	4,50	0,0015	ADH5	alcohol dehydrogenase 5 (class III), chi polypeptide"	NM_000671	Hs.78989
A_23_P127948	2,35	0,0060	1,55	0,0872	1,52	0,0992	ADM	adrenomedullin	NM_001124	Hs.441047
A_24_P378928	5,47	0,0114	5,39	0,0141	6,76	0,0018	ADRBK2	adrenergic, beta, receptor kinase 2"	NM_005160	Hs.657494
A_23_P373464	6,08	0,0000	5,93	0,0000	6,12	0,0000	AFF3	AF4/FMR2 family, member 3	NM_002285	Hs.444414
A_23_P53152	3,10	0,0098	3,67	0,0021	3,14	0,0094	AKIP1	A kinase (PRKA) interacting protein 1	NM_020642	Hs.131180
A_23_P116286	1,77	0,0355	2,03	0,0180	1,60	0,0797	AMPD3	adenosine monophosphate deaminase (isoform E)	NM_001025390	Hs.501890
A_24_P304154	1,72	0,0147	1,78	0,0122	1,50	0,0409	AMPD3	adenosine monophosphate deaminase 3	NM_001025390	Hs.501890
A_23_P126706	3,92	0,0746	4,79	0,0310	4,06	0,0830	ANGPTL1	angiopoietin-like 1	NM_004673	Hs.591474
A_24_P199484	4,22	0,0042	3,58	0,0161	3,86	0,0090	ANGPTL1	angiopoietin-like 1	NM_004673	Hs.591474
A_24_P306585	2,27	0,0040	1,95	0,0140	2,37	0,0025	ANKRD18CP			
A_23_P312565	2,74	0,0723	4,33	0,0036	4,08	0,0067	ANKRD19P	ankyrin repeat domain 19, pseudogene	NR_026868	Hs.643597
A_23_P385295	2,09	0,0097	1,57	0,0675	1,83	0,0286	AP1S3	adaptor-related protein complex 1, sigma 3 subunit"	BC021898	
A_23_P15113	3,20	0,0359	3,10	0,0524			ARHGAP17	Rho GTPase activating protein 17	NM_001006634	Hs.373793
A_24_P401739			5,47	0,0777	5,33	0,0922	ARHGAP17	Rho GTPase activating protein 17	NM_001006634	Hs.373793
A_24_P179516	2,61	0,0432	2,50	0,0666	2,37	0,0923	ARHGAP25	Rho GTPase activating protein 25	BC039591	Hs.531807
A_23_P216517	3,45	0,0127	3,54	0,0114	2,95	0,0420	ARHGEF39	chromosome 9 open reading frame 100	NM_032818	Hs.534579
A_24_P30034	2,84	0,0045	2,88	0,0036	2,64	0,0083	ARHGEF39	chromosome 9 open reading frame 100	NM_032818	Hs.534579
A_24_P921186	4,71	0,0671	5,87	0,0236	5,53	0,0354	ARL17	ADP-ribosylation factor-like 17	BC071681	Hs.559259
A_23_P130965	4,84	0,0183	4,94	0,0181	5,78	0,0051	ARRDC2	arrestin domain containing 2	NM_001025604	Hs.515249
A_23_P31921	1,68	0,0468	2,21	0,0089	1,86	0,0315	ASS1	argininosuccinate synthase 1	NM_000050	Hs.160786
A_23_P48278	2,24	0,0273	2,26	0,0305	1,95	0,0754	ATF7IP	activating transcription factor 7 interacting protein	AK001001	Hs.714407
A_24_P923757	5,35	0,0311	4,93	0,0609			ATF7IP	activating transcription factor 7 interacting protein	NM_018179	Hs.714407
A_24_P341987	2,69	0,0588	3,30	0,0214	2,75	0,0680	ATF7IP2	activating transcription factor 7 interacting protein 2	NM_024997	Hs.513343
A_24_P196382	3,66	0,0054	3,00	0,0258	3,86	0,0033	ATG3	ATG3 autophagy related 3 homolog (S. cerevisiae)	BC002830	Hs.477126
A_23_P35916	4,11	0,0302	3,47	0,0928	4,22	0,0408	ATM	ATM serine/threonine kinase	NM_000051	Hs.367437
A_23_P257155	4,98	0,0254	5,18	0,0237	5,15	0,0246	ATXN7	ataxin 7	NM_000333	Hs.476595
A_32_P809435	3,01	0,0029	3,11	0,0017	3,13	0,0018	AUTS2	autism susceptibility candidate 2	BC011643	Hs.654801
A_24_P299034	4,21	0,0143	3,86	0,0294	3,28	0,0784	BACH1-IT1	BTB and CNC homology 1, basic leucine zipper transcription factor 1"	NM_001011545	Hs.154276
A_32_P103771	4,91	0,0105	5,36	0,0052	5,08	0,0088	BAGE	B melanoma antigen	AF527552	Hs.624543
A_23_P213126	1,81	0,0718	2,30	0,0230	2,37	0,0188	BMP2K	BMP2 inducible kinase	AB015331	Hs.146551
A_23_P334282	3,72	0,0048	2,52	0,0744	3,45	0,0094	BMP2K	BMP2 inducible kinase	NM_017593	Hs.146551
A_23_P99614	3,80	0,0090	3,22	0,0317	2,99	0,0512	BTBD6	BTB (POZ) domain containing 6	NM_033271	Hs.7367
A_32_P404896	3,98	0,0220	4,31	0,0143	4,65	0,0078	BTBD6P1			
A_32_P27991	3,73	0,0063	3,45	0,0123	3,74	0,0065	BUB3		CR610181	Hs.663111
A_24_P938516	4,03	0,0099	3,61	0,0245	3,13	0,0606	C17orf85	chromosome 17 open reading frame 85	BC011733	Hs.120963
A_23_P3956	1,71	0,0390					C1QTNF1	C1q and tumor necrosis factor related protein 1	NM_198594	Hs.201398
A_24_P73862	2,12	0,0785	2,29	0,0677	2,65	0,0291	C1QTNF1	C1q and tumor necrosis factor related protein 1	NM_198594	Hs.201398
A_23_P87041	3,30	0,0480	3,64	0,0321	3,28	0,0624	C2CD2L		THC2655354	
A_23_P71855	3,25	0,0376	3,65	0,0220	3,11	0,0613	C5	complement component 5	NM_001735	Hs.494997
A_23_P213877	1,78	0,0344					C5orf42	chromosome 5 open reading frame 42	NM_023073	Hs.643420
A_32_P226009	2,06	0,0463	2,15	0,0447	2,03	0,0639	C5orf42	chromosome 5 open reading frame 42	NM_023073	Hs.643420
A_23_P92928	1,41	0,0445	1,39	0,0609	1,46	0,0458	C6	complement component 6	NM_000065	Hs.481992
A_32_P145159	2,21	0,0780	2,76	0,0290	2,58	0,0449	C6orf120			
A_24_P303647	3,05	0,0123	3,09	0,0124	2,94	0,0182	C7orf60	chromosome 7 open reading frame 60	NM_152556	Hs.489734
A_24_P330518	2,07	0,0890	2,47	0,0464	2,68	0,0287	CA12	carbonic anhydrase XII	NM_001218	Hs.210995
A_23_P217570	1,93	0,0594	2,10	0,0472	2,16	0,0411	CAPN6	calpain 6	NM_014289	Hs.496593
A_23_P52676	3,50	0,0069	2,86	0,0319	3,00	0,0237	CATSPER1	cation channel, sperm associated 1	NM_053054	Hs.189105
A_23_P127117	3,61	0,0437	3,85	0,0363	3,74	0,0451	CCDC6	coiled-coil domain containing 6	S72869	Hs.591360
A_24_P234921	1,74	0,0098	1,55	0,0252	1,75	0,0105	CCR6	chemokine (C-C motif) receptor 6	NM_031409	Hs.46468
A_23_P209055	9,02	0,0000			10,61	0,0000	CD22	CD22 molecule	NM_001771	Hs.579691

A_23_P351286	7,97	0,0015			6,99	0,0048	CD22	CD22 molecule	AK026467	
A_24_P254106	6,35	0,0039	4,31	0,0629	5,22	0,0197	CD22	CD22 molecule	AK026467	
A_24_P279307	3,14	0,0132	2,54	0,0587	2,82	0,0317	CD300LF	CD300 molecule-like family member f	NM_139018	Hs.567706
A_23_P23829	2,14	0,0000	1,99	0,0000	2,07	0,0000	CD34	CD34 molecule	NM_001773	Hs.374990
A_23_P87879	3,33	0,0029	2,65	0,0184	3,52	0,0015	CD69	CD69 molecule	NM_001781	Hs.208854
A_23_P70670	8,69	0,0059	6,52	0,0479	9,55	0,0025	CD83	CD83 molecule	NM_004233	Hs.595133
A_32_P7502	4,91	0,0082	4,70	0,0124	5,23	0,0051	CD96	CD96 molecule	NM_198196	Hs.142023
A_23_P98335	1,39	0,0993	1,90	0,0223	1,62	0,0617	CDON	Cdon homolog (mouse)	NM_016952	Hs.38034
A_23_P33384	2,45	0,0711					CIITA	class II, major histocompatibility complex, transactivator"	NM_000246	Hs.701991
A_23_P353478	4,73	0,0977			5,05	0,0963	CIITA	class II, major histocompatibility complex, transactivator"	U18259	Hs.126714
A_24_P923807	5,77	0,0042	5,99	0,0026	5,51	0,0063	CIITA	hypothetical LOC730038	XM_001134271	
A_32_P209960	8,01	0,0000	6,69	0,0003	8,30	0,0000	CIITA	class II, major histocompatibility complex, transactivator	NM_000246	Hs.701991
A_23_P253734	3,42	0,0859	3,88	0,0591	3,58	0,0933	CLN8	ceroid-lipofuscinosis, neuronal 8 (epilepsy, progressive with mental retardatio	NM_018941	Hs.127675
A_23_P310931	2,90	0,0171	3,01	0,0147	2,80	0,0246	CNR2	cannabinoid receptor 2 (macrophage)	NM_001841	Hs.73037
A_23_P134614	4,35	0,0203	3,41	0,0953	4,32	0,0246	COG5	component of oligomeric golgi complex 5	NM_006348	Hs.239631
A_23_P501010	2,17	0,0075	2,18	0,0077	2,32	0,0045	COL17A1	collagen, type XVII, alpha 1	NM_000494	Hs.117938
A_23_P375922	3,06	0,0101	2,58	0,0366	2,99	0,0135	COL19A1	collagen, type XIX, alpha 1	NM_001858	Hs.444842
A_23_P204033	2,95	0,0753	3,27	0,0570	3,42	0,0446	CPSF6	cleavage and polyadenylation specific factor 6, 68kDa"	NM_007007	Hs.369606
A_23_P16283	2,64	0,0252	2,26	0,0754	2,26	0,0776	CPT1C	carnitine palmitoyltransferase 1C	NM_152359	Hs.112195
A_23_P256821	2,71	0,0593	3,41	0,0180	3,43	0,0174	CR1	complement component (3b/4b) receptor 1 (Knops blood group)	NM_000651	Hs.334019
A_24_P265523	3,02	0,0060	2,78	0,0122	3,05	0,0058	CR1	complement component (3b/4b) receptor 1 (Knops blood group)	NM_000651	Hs.334019
A_23_P397671	3,39	0,0289	3,39	0,0338	3,76	0,0176	CR1L	complement component (3b/4b) receptor 1 (Knops blood group)	NM_000651	Hs.334019
A_24_P56894	4,82	0,0085	3,77	0,0499	4,19	0,0264	CRLF3	cytokine receptor-like factor 3	NM_015986	Hs.370168
A_32_P109242	1,07	0,0442	0,97	0,0899	1,03	0,0684	CSRNP3		AK055302	Hs.609017
A_23_P209625	1,01	0,0458	1,51	0,0024	1,34	0,0080	CYP1B1	cytochrome P450, family 1, subfamily B, polypeptide 1	NM_000104	Hs.154654
A_24_P995	2,32	0,0316	2,01	0,0851	2,22	0,0509	DCAF13	WD repeats and SOF1 domain containing	AF161549	Hs.532265
A_23_P311346	2,00	0,0420			1,93	0,0645	DENND2D	DENN/MADD domain containing 2D	AL713773	Hs.557850
A_23_P85952	2,87	0,0175	2,97	0,0155	2,73	0,0287	DENND2D	DENN/MADD domain containing 2D	NM_024901	Hs.557850
A_23_P321201	1,91	0,0098	2,01	0,0069	1,61	0,0368	DENND5A	DENN/MADD domain containing 5A	NM_015213	Hs.501857
A_24_P922808	1,67	0,0265	1,59	0,0434	1,51	0,0604	DESI2	family with sequence similarity 152, member A"	BC020640	Hs.498317
A_23_P54441	5,15	0,0068	5,00	0,0091	4,56	0,0192	DEXI	dexamethasone-induced transcript	NM_014015	Hs.592051
A_24_P144377	5,76	0,0026	5,21	0,0057	6,30	0,0009	DEXI	Dexi homolog (mouse)	NM_014015	Hs.592051
A_23_P29118	2,88	0,0218	2,96	0,0210	2,96	0,0217	DGCR5	DiGeorge syndrome critical region gene 5 (non-protein coding)	NR_002733	Hs.646438
A_24_P7179	4,15	0,0152	5,26	0,0020	4,30	0,0133	DIP2A	DIP2 disco-interacting protein 2 homolog A (Drosophila)	NM_206890	Hs.189585
A_23_P65741	4,06	0,0389	3,97	0,0542	3,58	0,0967	DIS3L	DIS3 like exosome 3'-5' exoribonuclease	NM_133375	Hs.446251
A_24_P128255	5,55	0,0067	4,20	0,0506	4,57	0,0314	DKFZP586B031	DKFZP586B0319 protein		Hs.667735
A_23_P353541	1,84	0,0661	1,94	0,0640	1,84	0,0861	DLX6	distal-less homeobox 6	NM_005222	Hs.249196
A_23_P213199	4,87	0,0333	4,15	0,0953	4,93	0,0376	DNAJB14	DnaJ (Hsp40) homolog, subfamily B, member 14	NM_001031723	Hs.577426
A_24_P334402			5,84	0,0524			DNAJB14	DnaJ (Hsp40) homolog, subfamily B, member 14"	NM_024920	Hs.577426
A_24_P76158	4,09	0,0063	5,41	0,0003	4,70	0,0017	DOCK11	dedicator of cytokinesis 11	NM_144658	Hs.368203
A_32_P743184	4,04	0,0056	3,89	0,0077	3,60	0,0150	DPPA3	developmental pluripotency associated 3	NM_199286	Hs.131358
A_32_P73139	1,68	0,0802	2,14	0,0267	2,16	0,0253	DPY19L2P3		BC047708	Hs.652669
A_23_P99163	4,49	0,0022	3,94	0,0065	4,19	0,0038	DRAM1	DNA-damage regulated autophagy modulator 1	NM_018370	Hs.525634
A_24_P355816	4,24	0,0046	4,26	0,0040	4,17	0,0053	DRAM1	damage-regulated autophagy modulator	NM_018370	Hs.525634
A_23_P54291	3,77	0,0290	3,29	0,0755	3,26	0,0824	DUOX1	dual oxidase 1	NM_017434	Hs.272813
A_23_P143650	4,68	0,0024	3,87	0,0121	4,53	0,0030	DUSP18	dual specificity phosphatase 18	NM_152511	Hs.517544
A_24_P221414	2,32	0,0130	1,96	0,0449	2,12	0,0274	DYNC1I1	dynein, cytoplasmic 1, intermediate chain 1	NM_004411	Hs.440364
A_24_P315739	1,61	0,0268	1,39	0,0753	1,35	0,0913	DZANK1	chromosome 20 open reading frame 12	NM_018152	Hs.472225
A_23_P9621	3,37	0,0144	3,33	0,0178	2,99	0,0372	ELF4	E74-like factor 4 (ets domain transcription factor)	NM_001421	Hs.271940
A_23_P117580	3,41	0,0060	3,77	0,0022	3,68	0,0031	ENTPD5	ectonucleoside triphosphate diphosphohydrolase 5	NM_001249	Hs.131555
A_23_P95599	1,68	0,0338	1,65	0,0452	1,46	0,0899	ERVK13-1		NM_001012731	Hs.647398
A_32_P195401	4,47	0,0000	4,85	0,0000	4,96	0,0000	FAM117B	family with sequence similarity 117, member B	NM_173511	Hs.471130
A_23_P345799	5,61	0,0250	5,30	0,0429	6,85	0,0067	FAM129C	family with sequence similarity 129, member C	NM_173544	Hs.434133
A_24_P940348	4,78	0,0496			4,74	0,0659	FAM129C	family with sequence similarity 129, member C"	NM_173544	Hs.434133
A_24_P266048	3,06	0,0065	3,14	0,0052	3,15	0,0055	FAM134B	family with sequence similarity 134, member B	NM_001034850	Hs.481704
A_23_P334955	1,40	0,0220	1,37	0,0290	1,36	0,0315	FAM167A	family with sequence similarity 167, member A	NM_053279	Hs.124299
A_23_P432554	2,60	0,0102	2,61	0,0107	2,49	0,0160	FBXO36	F-box protein 36	NM_174899	Hs.140666
A_23_P146811	4,54	0,0014	4,27	0,0021	4,48	0,0015	FBXO38	F-box protein 38	NM_030793	Hs.483772
A_23_P164773	3,84	0,0040	4,29	0,0011	4,61	0,0006	FCER2	Fc fragment of IgE, low affinity II, receptor for (CD23)	NM_002002	Hs.465778

A_23_P149368	6,23	0,0000	5,12	0,0000	5,83	0,0000	FCRL1	Fc receptor-like 1	NM_052938	Hs.656112
A_24_P328259	5,01	0,0135	5,34	0,0090	6,29	0,0020	FCRL1	Fc receptor-like 1	NM_052938	Hs.656112
A_32_P71876	6,09	0,0000	5,56	0,0000	6,12	0,0000	FCRL1		THC2767659	
A_23_P160751	2,84	0,0669	3,22	0,0426	3,55	0,0231	FCRL2	Fc receptor-like 2	NM_030764	Hs.437393
A_24_P319647	5,67	0,0235	4,91	0,0649	4,60	0,0943	FCRL2	Fc receptor-like 2	NM_030764	Hs.437393
A_23_P316447	3,10	0,0076	3,02	0,0097	3,14	0,0073	FGFR1	fibroblast growth factor receptor 1	ENST0000049629	Hs.264887
A_23_P122210	2,28	0,0407	2,23	0,0565	2,03	0,0949	FLJ11292	hypothetical protein FLJ11292	AK023417	Hs.714581
A_23_P3922	1,31	0,0078	1,00	0,0543	0,94	0,0782	FLJ11710	hypothetical protein FLJ11710	AK021772	Hs.657294
A_24_P187874	4,26	0,0036	4,31	0,0028	4,15	0,0044	FLJ40712	FLJ40712 protein	AK098031	Hs.683800
A_23_P155257	2,70	0,0968	2,95	0,0826	3,48	0,0325	FOXP1	forkhead box P1	NM_032682	Hs.431498
A_23_P93302	5,05	0,0019	5,35	0,0008	5,29	0,0010	GABBR1	gamma-aminobutyric acid (GABA) B receptor, 1	NM_001470	Hs.167017
A_24_P910667	4,09	0,0273	4,03	0,0359	4,91	0,0088	GAFA3	FGF-2 activity-associated protein 3	ENST0000062392	Hs.543647
A_23_P209459	1,77	0,0004	1,33	0,0067	1,19	0,0171	GALNT3		NM_004482	
A_23_P88194	3,08	0,0368	2,88	0,0647	3,04	0,0494	GEMIN2	survival of motor neuron protein interacting protein 1	NM_003616	Hs.708127
A_23_P66774	0,80	0,0678	0,94	0,0333	0,82	0,0761	GGT6	gamma-glutamyltransferase 6	NM_153338	Hs.130749
A_32_P39394	2,50	0,0115	2,23	0,0290	2,37	0,0194	GLIS3	GLIS family zinc finger 3	NM_001042413	Hs.162125
A_23_P104012	2,74	0,0083	2,81	0,0071	2,22	0,0417	GNAT2		ENST00000351050	
A_24_P393679	4,63	0,0007	4,53	0,0006	4,93	0,0002	GNL1	guanine nucleotide binding protein-like 1	AK095071	Hs.654655
A_32_P19608	2,23	0,0433	2,08	0,0769	2,58	0,0218	GNPDA2	glucosamine-6-phosphate deaminase 2	AY173948	Hs.21398
A_32_P217330	2,23	0,0543	2,16	0,0800	2,31	0,0575	GOSR1		THC2537951	
A_23_P128808	2,79	0,0233	2,89	0,0216	2,32	0,0824	GPR132	G protein-coupled receptor 132	NM_013345	Hs.532504
A_23_P14165	5,24	0,0000	4,81	0,0000	5,29	0,0000	GPR18	G protein-coupled receptor 18	NM_005292	Hs.631765
A_23_P143720	3,42	0,0210	3,20	0,0374	3,17	0,0408	GRAP2	GRB2-related adaptor protein 2	NM_004810	Hs.517499
A_24_P10884	2,20	0,0207	1,92	0,0563	1,77	0,0899	GRAP2	GRB2-related adaptor protein 2	NM_004810	Hs.517499
A_24_P408449	3,52	0,0004	3,45	0,0003	3,53	0,0003	GRAP2		THC2663668	
A_24_P567952	3,55	0,0220	3,00	0,0689			HCG18	HLA complex group 18	CR606587	Hs.485041
A_24_P934162	3,71	0,0056	3,51	0,0091	3,53	0,0089	HCG18		AK128413	Hs.663638
A_23_P211673	1,52	0,0028	1,13	0,0285	1,11	0,0327	HDAC10	SLIT and NTRK-like family, member 2"	AY358828	Hs.320368
A_23_P210048	1,72	0,0036	1,16	0,0625	1,09	0,0868	HDAC4	histone deacetylase 4	NM_006037	Hs.20516
A_23_P309292	4,86	0,0098	4,43	0,0214	4,12	0,0353	HECTD4	HECT domain containing E3 ubiquitin protein ligase 4	NM_001109662	Hs.695995
A_23_P137503	2,68	0,0059	1,78	0,0941	1,98	0,0536	HHLA3	HERV-H LTR-associating 3	NM_001036645	Hs.142245
A_23_P154345	2,12	0,0078	2,09	0,0093	1,67	0,0460	HIBCH	3-hydroxyisobutyryl-CoA hydrolase	NM_014362	Hs.656685
A_24_P481844	7,32	0,0006	9,39	0,0000	9,42	0,0000	HLA-DMB	major histocompatibility complex, class II, DM beta"	BC035650	Hs.654428
A_32_P351968	4,08	0,0919			5,29	0,0292	HLA-DMB	major histocompatibility complex, class II, DM beta	NM_002118	Hs.654428
A_23_P8108					3,55	0,0743	HLA-DQB1	major histocompatibility complex, class II, DQ beta 1	NM_001243962	Hs.409934
A_24_P937649	5,65	0,0104	4,39	0,0609	5,80	0,0094	HLA-DQB1	major histocompatibility complex, class II, DQ beta 1"	AK097297	Hs.409934
A_24_P248251	1,80	0,0254	1,80	0,0299	1,80	0,0306	HLTF	helicase-like transcription factor	NM_139048	Hs.3068
A_23_P434900	3,89	0,0022	3,68	0,0032	3,65	0,0037	HN1L	hematological and neurological expressed 1-like	NM_144570	Hs.513261
A_23_P206760	3,42	0,0098	2,52	0,0769	2,44	0,0938	HP	haptoglobin	NM_005143	Hs.513711
A_24_P362969	2,09	0,0163	2,04	0,0215	1,60	0,0933	HSD17B13	hydroxysteroid (17-beta) dehydrogenase 13	NM_178135	Hs.284414
A_23_P17224	4,09	0,0172	4,03	0,0220	4,29	0,0141	ICA1L	islet cell autoantigen 1,69kDa-like	NM_138468	Hs.516629
A_23_P430168	3,23	0,0063	3,27	0,0056	3,24	0,0064	ICA1L	islet cell autoantigen 1,69kDa-like	ENST0000048456	Hs.516629
A_24_P211797	3,27	0,0206	2,96	0,0452	3,15	0,0311	ICA1L	islet cell autoantigen 1,69kDa-like	NM_001288624	Hs.516629
A_23_P129556	6,35	0,0000	4,09	0,0026	6,04	0,0000	IL4R	interleukin 4 receptor	NM_000418	Hs.513457
A_23_P71037	2,70	0,0551	2,95	0,0500	3,12	0,0368	IL6	interleukin 6	NM_000600	Hs.654458
A_23_P125686	1,33	0,0625	1,47	0,0462	1,51	0,0404	INGX	inhibitor of growth family, X-linked, pseudogene	NR_002226	Hs.567456
A_23_P61149	4,84	0,0002	3,56	0,0038	4,25	0,0006	INPP5D	inositol polyphosphate-5-phosphatase, 145kDa	NM_001017915	Hs.601911
A_24_P912074	5,66	0,0251					INPP5D	inositol polyphosphate-5-phosphatase, 145kDa"	AK023492	
A_23_P8848	4,54	0,0063	5,42	0,0011	4,43	0,0081	INTS9	integrator complex subunit 9	NM_018250	Hs.162397
A_23_P202696	3,29	0,0333	3,53	0,0256	3,46	0,0296	KBTBD4	kelch repeat and BTB (POZ) domain containing 4	NM_016506	Hs.656205
A_24_P135406	3,21	0,0206	3,36	0,0172	3,94	0,0047	KCTD9	potassium channel tetramerization domain containing 9	NM_017634	Hs.72071
A_24_P102434	2,55	0,0212	2,94	0,0084	3,16	0,0045	KIAA0922	KIAA0922	NM_015196	Hs.205572
A_32_P61857	1,94	0,0730	2,05	0,0690	2,05	0,0730	KIAA1468	KIAA1468	NM_020854	Hs.465323
A_23_P412764	1,89	0,0420	2,14	0,0239	2,14	0,0244	KIAA1958	KIAA1958	ENST0000033753	Hs.656619
A_23_P122027	2,61	0,0904	2,96	0,0622	2,72	0,0991	KIF3A	kinesin family member 3A	NM_007054	Hs.43670
A_23_P120325	2,03	0,0637	2,23	0,0479	2,34	0,0372	KIF3C	kinesin family member 3C	NM_002254	Hs.21611
A_32_P154473	4,13	0,0069	3,83	0,0134	4,07	0,0081	KIF5C	kinesin family member 5C	NM_004522	Hs.660699
A_32_P33114	3,61	0,0065	3,89	0,0032	3,54	0,0080	KLB	klotho beta	NM_175737	Hs.90756
A_24_P208721	4,62	0,0058	3,73	0,0304	3,51	0,0451	KLHL18	kelch-like 18 (Drosophila)	NM_025010	Hs.517946

A_23_P121527	2,23	0,0574	3,11	0,0073	2,92	0,0128	KLHL5	kelch-like family member 5	NM_015990	Hs.272251
A_23_P218058	2,11	0,0362	1,99	0,0609	1,87	0,0876	KLRC4	killer cell lectin-like receptor subfamily C, member 4	NM_013431	Hs.387787
A_23_P56898	2,99	0,0425	3,29	0,0291	3,59	0,0162	KYNU	kynureninase (L-kynurenine hydrolase)	NM_003937	Hs.470126
A_23_P210445	3,43	0,0034	2,15	0,0855	2,45	0,0425	L3MBTL1	l(3)mbt-like 1 (Drosophila)	NM_032107	Hs.709356
A_23_P311885	2,79	0,0104	2,93	0,0075	2,77	0,0122	L3MBTL3	l(3)mbt-like 3 (Drosophila)	NM_032438	Hs.658051
A_24_P391918	3,52	0,0127	3,72	0,0088	3,49	0,0155	L3MBTL4	l(3)mbt-like 4 (Drosophila)	NM_173464	Hs.128279
A_23_P86283					3,62	0,0509	LAPTM5	lysosomal protein transmembrane 5	NM_006762	Hs.371021
A_24_P114124	2,20	0,0543	2,18	0,0711	2,66	0,0217	LAPTM5	lysosomal multispanning membrane protein 5	NM_006762	Hs.371021
A_23_P54357	1,86	0,0229	1,76	0,0367	1,56	0,0776	LDHAL6B	lactate dehydrogenase A-like 6B	NM_033195	Hs.307052
A_23_P8452	4,13	0,0026	4,61	0,0006	4,40	0,0012	LFNG	LFNG O-fucosylpeptide 3-beta-N-acetylglucosaminyltransferase	NM_001040167	Hs.159142
A_23_P149441	4,10	0,0237	4,37	0,0183	3,37	0,0899	LINC00328	melanoma antigen	AF172850	Hs.633593
A_32_P196036	1,47	0,0092	1,29	0,0265	1,41	0,0139	LINC01128	hypothetical LOC643837	CR594200	Hs.133183
A_32_P116606	1,42	0,0138	1,29	0,0301	1,34	0,0234	LIX1	Lix1 homolog (chicken)	NM_153234	Hs.656702
A_24_P400832	3,35	0,0115	2,78	0,0445	2,49	0,0841	LOC100130950	uncharacterized LOC100130950	NR_034082	Hs.668646
A_23_P331908	2,40	0,0424	2,64	0,0291	2,66	0,0280	LOC101060179	uncharacterized LOC101060179	XM_003959932	Hs.178715
A_24_P935310	3,16	0,0115	4,02	0,0012	2,70	0,0376	LOC222159	hypothetical protein LOC222159	AK027340	Hs.561708
A_24_P195936	4,49	0,0103	4,65	0,0085	4,27	0,0171	LOC440180	hypothetical LOC440180	XR_018312	Hs.647575
A_24_P161813	2,42	0,0191	2,04	0,0616	2,46	0,0194	LOC441666	zinc finger protein 91 pseudogene	NR_024380	
A_24_P316059	2,96	0,0297	2,66	0,0651	2,78	0,0523	LOC441768	hypothetical LOC441768	XR_019199	
A_24_P910024	1,97	0,0265	1,73	0,0680	1,80	0,0570	LOC645478	hypothetical LOC645478	AK055364	Hs.657558
A_32_P104619	5,18	0,0028	4,15	0,0169	5,50	0,0014	LOC727820	hypothetical protein LOC727820	AK094156	Hs.655890
A_24_P358084	2,39	0,0131	1,99	0,0484	1,78	0,0922	LOC729602	NPIP-like protein ENSP00000283050	ENST0000053293[illegible]	Hs.681787
A_23_P46606	3,17	0,0331	3,81	0,0110	3,85	0,0103	LPGAT1	lysophosphatidylglycerol acyltransferase 1	NM_014873	Hs.497674
A_24_P114551	4,41	0,0194	3,78	0,0583	4,17	0,0330	LPP	LIM domain containing preferred translocation partner in lipoma	NM_005578	Hs.444362
A_23_P128447	2,13	0,0422	2,00	0,0749	2,17	0,0480	LRRK2	leucine-rich repeat kinase 2	NM_198578	Hs.187636
A_23_P354074	2,68	0,0363	2,57	0,0561	3,23	0,0128	LYST	lysosomal trafficking regulator	NM_000081	Hs.532411
A_24_P268196	1,88	0,0199	1,90	0,0212	2,08	0,0111	LZIC	leucine zipper and CTNNBIP1 domain containing	ENST0000037722[illegible]	Hs.327252
A_23_P114445	2,63	0,0023	1,68	0,0615	1,62	0,0778	MAGEE1	melanoma antigen family E, 1	NM_020932	Hs.8453
A_23_P57277	2,36	0,0128	2,34	0,0149	2,16	0,0261	MAP3K7CL	MAP3K7 C-terminal like	NM_020152	Hs.222802
A_24_P237265	2,63	0,0034	2,63	0,0029	2,39	0,0075	MAPK1	mitogen-activated protein kinase 1	NM_002745	Hs.431850
A_24_P354412	3,94	0,0002	3,50	0,0007	3,19	0,0021	MARCH1		AK091335	Hs.603066
A_23_P68539	2,23	0,0003	1,91	0,0012	1,57	0,0086	MAVS	virus-induced signaling adapter	ENST0000037956[illegible]	Hs.570362
A_23_P212073	3,44	0,0157	3,09	0,0363	4,01	0,0051	MBNL1	muscleblind-like (Drosophila)	NM_207296	Hs.478000
A_23_P7201	3,41	0,0184	3,80	0,0091	3,71	0,0115	MED28	mediator complex subunit 28	AF317680	Hs.434075
A_24_P926960	5,51	0,0000	5,14	0,0000	5,39	0,0000	MEGF6	multiple EGF-like-domains 6	NM_001409	Hs.593645
A_23_P365705	3,11	0,0077	3,75	0,0013	3,18	0,0068	METTL16	methyltransferase 10 domain containing	NM_024086	Hs.632237
A_23_P218523	3,26	0,0095	2,66	0,0424	2,47	0,0680	MFSD12	chromosome 19 open reading frame 28	NM_021731	Hs.656901
A_24_P153448	2,16	0,0264					MFSD12	chromosome 19 open reading frame 28	AF218008	Hs.656901
A_32_P221256	4,78	0,0327	5,89	0,0088	5,23	0,0222	MGC70870	C-terminal binding protein 2 pseudogene	NR_003682	Hs.572501
A_23_P300847	2,57	0,0147	2,21	0,0449	2,10	0,0615	MIR1247	microRNA 1247	ENST0000055588[illegible]	Hs.525597
A_23_P148439	1,96	0,0047	1,64	0,0195	1,48	0,0399	MKRN4P		THC2487640	
A_23_P127727	1,51	0,0440	1,46	0,0656	1,45	0,0699	MPEG1	macrophage expressed 1	AK097881	Hs.709439
A_32_P187176	4,00	0,0260	3,74	0,0472	4,28	0,0202	MRRFP1	mitochondrial ribosome recycling factor	NM_138777	Hs.368011
A_23_P116371	5,29	0,0000	3,04	0,0080	5,13	0,0000	MS4A1	membrane-spanning 4-domains, subfamily A, member 1"	NM_152866	Hs.712553
A_23_P61426	3,34	0,0210	3,93	0,0068	3,40	0,0221	MSRA	methionine sulfoxide reductase A	NM_012331	Hs.490981
A_24_P74828	2,48	0,0089	2,35	0,0149	2,14	0,0287	MT1JP	metallothionein 1J (pseudogene)	AF348994	Hs.513626
A_24_P239176	2,89	0,0617	2,81	0,0879			MUC4	mucin 4, cell surface associated	NM_018406	Hs.369646
A_24_P239177	2,66	0,0231	2,31	0,0636			MUC4	mucin 4, cell surface associated	NM_018406	Hs.369646
A_24_P239183	4,05	0,0033	3,22	0,0207	3,18	0,0230	MUC4	mucin 4, cell surface associated"	ENST0000038192[illegible]	Hs.369646
A_23_P76151	3,72	0,0009	3,03	0,0056	3,36	0,0021	NAB2	NGFI-A binding protein 2 (EGR1 binding protein 2)	NM_005967	Hs.159223
A_23_P319153	2,04	0,0304	1,93	0,0511	1,97	0,0457	NADSYN1	NAD synthetase 1	AL512694	Hs.556986
A_23_P427039	4,11	0,0132	4,34	0,0095	3,40	0,0521	NANP	N-acetylneuraminic acid phosphatase	NM_152667	Hs.666255
A_24_P101072	2,61	0,0227	2,87	0,0136	2,30	0,0586	NBPF10		ENST00000369326	
A_32_P171181	4,61	0,0992					NBPF10	neuroblastoma breakpoint family, member 10	NM_001039703	Hs.515947
A_32_P83465	2,61	0,0260	2,80	0,0201	2,24	0,0795	NBPF10	neuroblastoma breakpoint family, member 10	NM_001039703	Hs.515947
A_24_P135061	3,00	0,0123	3,00	0,0136	2,55	0,0422	NBPF11	hypothetical protein LOC200030	NM_183372	Hs.636561
A_24_P625683	3,13	0,0244	3,16	0,0274	2,72	0,0698	NBPF17P		XR_018208	
A_24_P345081	2,90	0,0076	3,01	0,0057	2,49	0,0259	NBPF2P	neuroblastoma breakpoint family, member 3	NM_032264	Hs.325422
A_24_P396889	4,02	0,0596	3,97	0,0787	3,90	0,0897	NDUFA10		AK025576	Hs.658209

A_23_P152873	2,21	0,0540					NEK8	NIMA (never in mitosis gene a)- related kinase 8	NM_178170	Hs.448468
A_24_P99639	2,31	0,0224	2,06	0,0520	2,32	0,0255	NEK8	NIMA-related kinase 8	NM_178170	Hs.448468
A_24_P356830	2,67	0,0040	2,60	0,0046	1,99	0,0376	NFIC		AK129956	Hs.170131
A_23_P383422	7,33	0,0000	7,22	0,0000	6,55	0,0000	NFKBID	nuclear factor of kappa light polypeptide gene enhancer in B-cells inhibitor, d	NM_139239	Hs.466531
A_23_P359131	6,00	0,0039	5,87	0,0043	4,16	0,0575	NIPA1	non imprinted in Prader-Willi/Angelman syndrome 1	NM_144599	Hs.511797
A_23_P41021	2,53	0,0006	1,59	0,0321	1,59	0,0336	NISCH	nischarin	NM_007184	Hs.435290
A_23_P338519	3,37	0,0115	3,31	0,0148	3,40	0,0122	NKIRAS1	NFKB inhibitor interacting Ras-like 1	NM_020345	Hs.173202
A_32_P5040	4,51	0,0596	5,79	0,0155	4,31	0,0965	NOTCH2	Notch homolog 2 (Drosophila) N-terminal like	NM_203458	Hs.709526
A_32_P75695	4,56	0,0438	5,37	0,0191			NOTCH2	Notch homolog 2 (Drosophila) N-terminal like	AK022008	Hs.655156
A_24_P366777	4,30	0,0030	4,58	0,0013	3,29	0,0248	NOTCH2NL	Notch homolog 2 (Drosophila) N-terminal like	ENST0000057979:	Hs.709526
A_32_P89087	2,34	0,0011	1,44	0,0521	1,52	0,0385	NPIPB3		AL134462	Hs.632992
A_24_P725891	2,25	0,0600					NPIPB4	hypothetical LOC100190986	BC051311	Hs.648439
A_32_P114268	2,21	0,0420	2,08	0,0714	2,25	0,0467	NPIPB4			
A_32_P174151	0,99	0,0837					NPIPB4	similar to Uncharacterized protein KIAA0220	NR_002603	Hs.552700
A_23_P339954	1,57	0,0032	1,00	0,0753	1,11	0,0436	NPIPB9	nuclear pore complex interacting protein family, member B9	NM_001287251	Hs.710214
A_23_P62752	2,47	0,0310	2,15	0,0807	2,17	0,0802	NPPB	natriuretic peptide B	NM_002521	Hs.219140
A_23_P306867	4,44	0,0134	3,95	0,0330	4,00	0,0315	NR4A3	nuclear receptor subfamily 4, group A, member 3	NM_173199	Hs.279522
A_23_P144208	1,99	0,0966	2,11	0,0965	2,20	0,0811	OR5H8			
A_23_P52868	4,61	0,0014	3,28	0,0245	3,84	0,0074	OR8G2	olfactory receptor, family 8, subfamily G, member 2"		Hs.381319
A_23_P65386	3,18	0,0003	2,50	0,0035	1,80	0,0457	OTUB2	OTU deubiquitinase, ubiquitin aldehyde binding 2	NM_023112	Hs.278815
A_23_P132486	3,61	0,0179	3,04	0,0591	2,93	0,0751	OXSM	3-oxoacyl-ACP synthase, mitochondrial	NM_017897	Hs.55781
A_24_P69784	4,14	0,0735	4,75	0,0452	4,20	0,0921	PACS1	phosphofurin acidic cluster sorting protein 1	NM_018026	Hs.644326
A_24_P920398	3,75	0,0555			5,01	0,0104	PARP14	poly (ADP-ribose) polymerase family, member 14"	NM_017554	Hs.518203
A_32_P56759	3,92	0,0069	4,81	0,0009	4,29	0,0033	PARP14	poly (ADP-ribose) polymerase family, member 14"	NM_017554	Hs.518203
A_23_P59691	1,30	0,0064	1,04	0,0347	1,00	0,0449	PAX4	paired box 4	NM_006193	Hs.129706
A_23_P71624	3,46	0,0585	3,38	0,0810	4,23	0,0220	PAX5	paired box 5	NM_016734	Hs.654464
A_24_P555510	2,76	0,0048	2,22	0,0271	2,75	0,0051	PCM1	pericentriolar material 1	NM_006197	Hs.491148
A_24_P41918	6,10	0,0005	5,57	0,0010	5,84	0,0006	PCNXL4	hypothetical protein LOC100130173	BC030714	Hs.509499
A_23_P149153	3,64	0,0109	3,66	0,0113	2,90	0,0556	PDE4DIP	phosphodiesterase 4D interacting protein	NM_022359	Hs.654651
A_23_P123478	3,76	0,0923	4,26	0,0645	4,38	0,0570	PDE7A	phosphodiesterase 7A	NM_002603	Hs.584788
A_24_P360529	5,11	0,0464	5,21	0,0511	4,93	0,0724	PDE7A	phosphodiesterase 7A	AF332652	Hs.584788
A_24_P340112	3,50	0,0035	3,26	0,0058	3,04	0,0114	PDK3		THC2683124	
A_24_P941666	1,31	0,0976					PDK3		BC038512	Hs.296031
A_32_P215143	3,29	0,0216	2,70	0,0792			PDXDC2P		THC2598362	
A_32_P216872	3,12	0,0748			3,16	0,0943	PDXDC2P	pyridoxal-dependent decarboxylase domain containing 2, pseudogene	BX647358	Hs.513695
A_23_P89589	5,13	0,0023	4,06	0,0157	4,70	0,0048	PER1	period circadian clock 1	NM_002616	Hs.445534
A_24_P921232	5,62	0,0155	5,41	0,0228	4,88	0,0451	PEX14	peroxisomal biogenesis factor 14	BC017848	Hs.149983
A_23_P259833	5,13	0,0000	5,14	0,0000	5,17	0,0000	PIK3CD	phosphoinositide-3-kinase, catalytic, delta polypeptide"	NM_005026	Hs.518451
A_23_P121596	3,24	0,0432	3,45	0,0361	3,59	0,0287	PPBP	pro-platelet basic protein (chemokine (C-X-C motif) ligand 7)	NM_002704	Hs.2164
A_23_P15621	1,70	0,0039	1,24	0,0420	1,15	0,0685	PRAC1	prostate cancer susceptibility candidate	NM_032391	Hs.116467
A_23_P137248	2,12	0,0016			1,22	0,0921	PRKY	protein kinase, Y-linked, pseudogene	NR_028062	Hs.632287
A_24_P186030			4,18	0,0445			PRKY	protein kinase, Y-linked"	NM_002760	Hs.632287
A_23_P80156	4,92	0,0000	5,16	0,0000	4,82	0,0000	PRMT2	protein arginine methyltransferase 2	NM_206962	Hs.154163
A_23_P76851	2,02	0,0181	1,94	0,0274	1,74	0,0540	PRMT5	protein arginine methyltransferase 5	NM_001039619	Hs.367854
A_24_P344307	2,43	0,0420	2,85	0,0184	2,38	0,0592	PSME3	proteasome (prosome, macropain) activator subunit 3 (PA28 gamma; Ki)"	NM_176863	Hs.152978
A_23_P412029	3,49	0,0000	3,96	0,0000	3,93	0,0000	PUS10	pseudouridylate synthase 10	NM_144709	Hs.368348
A_23_P78158	3,93	0,0584	3,77	0,0902	4,14	0,0561	RABEP1	rabaptin, RAB GTPase binding effector protein 1	NM_004703	Hs.592121
A_24_P399174	4,22	0,0000	4,11	0,0000	4,12	0,0000	RABEP1	rabaptin, RAB GTPase binding effector protein 1	NM_004703	Hs.592121
A_24_P945147	5,03	0,0058	4,10	0,0285	4,67	0,0114	RABEP1	rabaptin, RAB GTPase binding effector protein 1	NM_004703	Hs.592121
A_23_P97517	4,41	0,0108	3,56	0,0504	4,03	0,0231	RALGPS2	Ral GEF with PH domain and SH3 binding motif 2	NM_018037	Hs.709811
A_23_P409966	3,48	0,0030	3,17	0,0062	3,25	0,0052	RAPH1	Ras association (RalGDS/AF-6) and pleckstrin homology domains 1	NM_025252	Hs.471162
A_23_P331186	3,32	0,0929	3,15	0,0009	3,27	0,0006	RASGEF1B	RasGEF domain family, member 1B	NM_152545	Hs.591696
A_24_P930432	3,52	0,0004					RASGEF1B	RasGEF domain family, member 1B	BX648337	Hs.591696
A_24_P364516	2,36	0,0224	2,16	0,0447	2,25	0,0359	RCBTB1	regulator of chromosome condensation (RCC1) and BTB (POZ) domain cont	NM_018191	Hs.508021
A_23_P56938	5,54	0,0200	4,68	0,0645	5,56	0,0227	REL	v-rel avian reticuloendotheliosis viral oncogene homolog	NM_002908	Hs.631886
A_24_P372625	2,36	0,0233	2,45	0,0212	2,00	0,0752	RNF141	ring finger protein 141	NM_016422	Hs.44685
A_23_P23542	4,47	0,0049	3,43	0,0363	3,60	0,0277	RPAP2	RNA polymerase II associated protein 2	NM_024813	Hs.444421
A_23_P394562	4,03	0,0255	4,13	0,0262	3,79	0,0458	RPGR		ENST00000339363	
A_23_P135079	2,44	0,0001	1,70	0,0027	1,68	0,0034	SARDH	sarcosine dehydrogenase	AF095737	Hs.198003

A_24_P115507	1,75	0,0763					SARDH	sarcosine dehydrogenase	NM_007101	Hs.198003
A_23_P98446	3,46	0,0016	2,91	0,0074	3,27	0,0026	SC5D	sterol-C5-desaturase (ERG3 delta-5-desaturase homolog, S. cerevisiae)-like	NM_001024956	Hs.287749
A_24_P162319	2,08	0,0200	2,28	0,0119	2,08	0,0230	SCML1	sex comb on midleg-like 1 (Drosophila)	NM_001037540	Hs.109655
A_24_P393181	1,97	0,0117	1,70	0,0359	1,81	0,0237	SCN11A	sodium channel, voltage-gated, type XI, alpha subunit"	AF150882	Hs.591657
A_23_P76006	2,54	0,0336	2,67	0,0296	2,52	0,0439	SERPINH1	serpin peptidase inhibitor, clade H (heat shock protein 47), member 1, (collag	NM_001235	Hs.596449
A_23_P361448	3,01	0,0009	2,59	0,0033	2,96	0,0009	SESN3	sestrin 3	NM_144665	Hs.659934
A_24_P918706	1,62	0,0825					SESN3		THC2534144	
A_23_P13946	6,01	0,0035	6,64	0,0011	5,83	0,0045	SH2B3	SH2B adaptor protein 3	NM_005475	Hs.506784
A_23_P100730	3,62	0,0142	2,82	0,0747	2,82	0,0782	SKAP1	src kinase associated phosphoprotein 1	NM_003726	Hs.316931
A_23_P205910	3,68	0,0459	3,91	0,0390	3,32	0,0993	SLC24A1	solute carrier family 24 (sodium/potassium/calcium exchanger), member 1"	NM_004727	Hs.173092
A_24_P401921	2,42	0,0229	2,16	0,0525	1,93	0,0993	SLC24A1	solute carrier family 24 (sodium/potassium/calcium exchanger), member 1"	AB014602	Hs.173092
A_23_P201248	5,23	0,0043	5,28	0,0035	5,16	0,0048	SLC26A9	solute carrier family 26 (anion exchanger), member 9	NM_052934	Hs.164073
A_23_P139669	6,23	0,0388					SLC2A3	solute carrier family 2 (facilitated glucose transporter), member 3	NM_006931	Hs.419240
A_24_P81900	5,32	0,0204	4,52	0,0629	6,01	0,0094	SLC2A3	solute carrier family 2 (facilitated glucose transporter), member 3	NM_006931	Hs.419240
A_23_P56494	3,51	0,0189	3,52	0,0210	3,61	0,0180	SLC38A11	solute carrier family 38, member 11	NM_173512	Hs.658702
A_23_P62070	2,05	0,0034	1,28	0,0887	1,31	0,0823	SLC6A19	solute carrier family 6 (neutral amino acid transporter), member 19"	AK096054	Hs.481478
A_23_P48936	2,30	0,0278	2,45	0,0220	2,19	0,0460	SMAD3	SMAD family member 3	NM_005902	Hs.716490
A_24_P942604	3,51	0,0599	3,91	0,0408	4,18	0,0271	SMC1A	structural maintenance of chromosomes 1A	NM_006306	Hs.211602
A_32_P218806	3,50	0,0788	4,43	0,0269	4,10	0,0449	SNAPC3		AK024516	Hs.657178
A_23_P21776	2,13	0,0792	2,15	0,0973	2,40	0,0568	SNAPC5	small nuclear RNA activating complex, polypeptide 5, 19kDa	ENST0000031663	Hs.30174
A_24_P219757	5,04	0,0007	5,39	0,0002	4,35	0,0028	SNRPA		THC2673303	
A_24_P150665	4,36	0,0248	3,67	0,0796	5,25	0,0074	SNX29	RUN domain containing 2A	NM_032167	Hs.458401
A_32_P107002					6,20	0,0202	SNX29	sorting nexin 29	NM_032167	Hs.658149
A_32_P66020	4,06	0,0448			6,03	0,0027	SNX29	sorting nexin 29	NM_032167	Hs.658149
A_24_P497464	3,42	0,0003	3,10	0,0007	2,48	0,0076	SOX9-AS1	SOX9 antisense RNA 1	NR_103738	Hs.657374
A_23_P209712	1,61	0,0440					SP100	SP100 nuclear antigen	CR749288	Hs.369056
A_24_P916816	3,95	0,0000	3,64	0,0000	3,99	0,0000	SP100	SP100 nuclear antigen	L79989	
A_24_P256526	4,70	0,0128	4,54	0,0184	4,82	0,0120	SP2	Sp2 transcription factor	BC005914	Hs.514276
A_32_P50655	1,65	0,0335	1,71	0,0324	1,52	0,0680	SPPL2A		THC2637707	Hs.595314
A_24_P136838	3,72	0,0181	3,73	0,0203	4,11	0,0096	SPPL3	signal peptide peptidase 3	NP500659	Hs.683964
A_23_P107048	1,56	0,0023	1,23	0,0160	1,05	0,0479	SUMO2		BC002811	Hs.679409
A_24_P1910	4,11	0,0634	3,93	0,0996	4,24	0,0704	SUPT4H1	suppressor of Ty 4 homolog 1 (S. cerevisiae)	NM_003168	Hs.439481
A_23_P42451	2,38	0,0436			2,62	0,0299	SYNE1	spectrin repeat containing, nuclear envelope 1"	AB051543	Hs.12967
A_24_P410453	2,69	0,0134	2,63	0,0173	2,46	0,0280	SYNE1	spectrin repeat containing, nuclear envelope 1"	NM_033071	Hs.12967
A_24_P109069	2,24	0,0666					SYT15	synaptotagmin XV	NM_181519	Hs.696346
A_32_P67904	2,15	0,0408	2,19	0,0449	2,36	0,0289	SYT15	synaptotagmin XV	AK131036	Hs.696346
A_23_P60047	2,71	0,0760	3,53	0,0210	2,72	0,0997	TACC1	transforming, acidic coiled-coil containing protein 1	NM_006283	Hs.279245
A_24_P191750	4,49	0,0040	3,69	0,0197	5,06	0,0012	TAPT1	transmembrane anterior posterior transformation 1	NM_153365	Hs.479223
A_23_P20139	2,05	0,0103	2,12	0,0085	1,87	0,0222	TAS2R3	taste receptor, type 2, member 3	NM_016943	Hs.676011
A_23_P62684	2,92	0,0076	2,36	0,0375	2,39	0,0361	TCEANC2		AF217973	
A_23_P205348	3,48	0,0599					TCL6	T-cell leukemia/lymphoma 6 (non-protein coding)	NR_028288	Hs.510368
A_23_P363203	2,58	0,0155	2,22	0,0471	2,40	0,0295	TCL6	T-cell leukemia/lymphoma 6 (non-protein coding)	NR_028288	Hs.510368
A_23_P80974	0,91	0,0825	1,13	0,0314	0,98	0,0769	TDO2	tryptophan 2,3-dioxygenase	NM_005651	Hs.183671
A_23_P166964	2,34	0,0976	2,97	0,0354	2,78	0,0546	TFDP2	transcription factor Dp-2 (E2F dimerization partner 2)	NM_006286	Hs.379018
A_24_P414169	2,35	0,0362	2,52	0,0281			TFDP2		BC040991	Hs.379018
A_23_P76267	3,74	0,0144	3,33	0,0351	3,29	0,0392	TMEM119	transmembrane protein 119	NM_181724	Hs.449718
A_24_P931889	2,54	0,0115	1,92	0,0757	1,84	0,0968	TMEM131		CD691495	Hs.469376
A_23_P39550	2,90	0,0239	2,67	0,0472	2,96	0,0253	TMEM163	transmembrane protein 163	NM_030923	Hs.369471
A_23_P94552	6,76	0,0017	5,59	0,0089	6,78	0,0015	TMEM2	transmembrane protein 2	NM_013390	Hs.494146
A_24_P261125	6,09	0,0000	5,80	0,0000	5,70	0,0000	TMEM2	transmembrane protein 2	NM_013390	Hs.494146
A_24_P510138	1,82	0,0240	2,00	0,0148	2,00	0,0151	TMEM232		AK093222	Hs.670269
A_24_P256583	2,26	0,0155	1,80	0,0726	2,04	0,0356	TMEM65	transmembrane protein 65	NM_194291	Hs.187646
A_23_P14174	2,88	0,0039	2,14	0,0369	2,54	0,0114	TNFSF13B	tumor necrosis factor (ligand) superfamily, member 13b	NM_006573	Hs.525157
A_23_P404134	3,08	0,0244	3,35	0,0161	3,73	0,0069	TOX4	TOX high mobility group box family member 4	ENST0000044879	Hs.555910
A_23_P209426	2,98	0,0103	3,47	0,0027	3,40	0,0037	TRAK2	trafficking protein, kinesin binding 2	NM_015049	Hs.152774
A_32_P194915	6,02	0,0000	5,82	0,0000	4,97	0,0000	TRAPPC5		THC2648197	
A_23_P99360	3,56	0,0063	3,33	0,0112	2,66	0,0523	TRIM13	tripartite motif containing 13	NM_213590	Hs.436922
A_23_P203498	3,62	0,0503			4,50	0,0160	TRIM22	tripartite motif-containing 22	NM_006074	Hs.501778
A_24_P172481	3,85	0,0047	3,03	0,0301	4,22	0,0019	TRIM22	tripartite motif containing 22	NM_006074	Hs.501778

A_23_P124190	4,86	0,0074	4,47	0,0151	4,49	0,0151	TRIM34	tripartite motif containing 34	NM_130390	Hs.125300
A_24_P153853	2,95	0,0036	3,02	0,0024	3,11	0,0020	TRIM37	tripartite motif containing 37	NM_001005207	Hs.579079
A_23_P29985	2,96	0,0092	2,93	0,0105	2,10	0,0921	TRMT44	tRNA methyltransferase 44 homolog (S. cerevisiae)	AK022953	Hs.566191
A_24_P461547	2,78	0,0467	3,18	0,0253	2,81	0,0553	TSC22D2			
A_24_P221869	4,24	0,0031	4,30	0,0023	4,35	0,0022	TSPY10	similar to testis specific protein, Y-linked 1	AK093413	Hs.556121
A_24_P179277	3,67	0,0132	3,46	0,0226	2,88	0,0709	TSPYL1	TSPY-like 1	AK096882	Hs.458358
A_23_P92517	2,81	0,0024	1,95	0,0408	1,85	0,0569	TTC29	tetratricopeptide repeat domain 29	NM_031956	Hs.378893
A_23_P401098	3,33	0,0107	3,06	0,0220	3,05	0,0229	TTC39C	tetratricopeptide repeat domain 39C	NM_153211	Hs.653395
A_32_P123629	2,33	0,0634	2,59	0,0447	2,51	0,0559	TTC39C	tetratricopeptide repeat domain 39C	NM_001292030	Hs.653395
A_23_P40156	2,01	0,0757	2,41	0,0359	2,20	0,0635	TTPAL	tocopherol (alpha) transfer protein-like	NM_024331	Hs.283869
A_23_P143926	1,12	0,0206	1,26	0,0097	0,91	0,0813	ULK4	unc-51-like kinase 4 (C. elegans)	BC014794	Hs.656192
A_32_P209582	1,62	0,0240	1,63	0,0277	1,59	0,0323	UNQ6228	hypothetical LOC100131541	THC2663167	
A_23_P207736	3,15	0,0871	3,40	0,0769			USP36		AK023077	Hs.464243
A_24_P367864	5,13	0,0213			5,40	0,0176	USP36	ubiquitin specific peptidase 36	BC038983	Hs.464243
A_23_P146468	3,45	0,0159	3,33	0,0228	3,34	0,0227	VAV2	vav 2 guanine nucleotide exchange factor	NM_003371	Hs.369921
A_24_P64895	4,90	0,0004	5,17	0,0002	5,25	0,0001	WDFY2		THC2781306	
A_24_P102920	3,52	0,0010	2,83	0,0075	2,90	0,0063	WDR33	WD repeat domain 33	NM_001006622	Hs.620490
A_23_P211302	3,77	0,0284	4,94	0,0039	3,97	0,0248	WDR4	WD repeat domain 4	NM_033661	Hs.248815
A_24_P943429	3,29	0,0058	2,94	0,0148	2,79	0,0224	WNK1			
A_23_P4283	3,30	0,0185	3,08	0,0332	3,42	0,0167	XAF1	XIAP associated factor 1	NM_017523	Hs.441975
A_23_P4286	4,35	0,0017	3,03	0,0314	3,30	0,0181	XAF1	XIAP associated factor 1	NM_017523	Hs.441975
A_24_P538708	3,25	0,0543	3,31	0,0609	3,25	0,0699	ZDHHC21		AK124263	Hs.649522
A_23_P100959	2,07	0,0048	1,59	0,0347	1,46	0,0601	ZFP3	zinc finger protein 3 homolog (mouse)	NM_153018	Hs.48832
A_23_P94063	1,46	0,0046	0,98	0,0749	0,94	0,0992	ZKSCAN1	zinc finger with KRAB and SCAN domains 1	AY260738	Hs.615360
A_24_P11100	4,27	0,0181	3,97	0,0334	3,88	0,0394	ZMAT1	zinc finger, matrin-type 1	NM_001282400	Hs.496512
A_23_P104146	3,41	0,0073	4,61	0,0003	3,91	0,0021	ZMYM4	zinc finger, MYM-type 4	NM_005095	Hs.269211
A_23_P131834	1,33	0,0778	1,64	0,0305	1,64	0,0309	ZMYND8		THC2485189	
A_23_P215806	2,56	0,0923	2,70	0,0918	3,00	0,0536	ZNF117	zinc finger protein 117	NM_024498	Hs.250693
A_23_P325661	3,72	0,0000	3,80	0,0000	3,74	0,0000	ZNF134	zinc finger protein 134	NM_003435	Hs.469694
A_24_P16950	2,16	0,0853					ZNF160	zinc finger protein 160	NM_198893	Hs.655967
A_24_P401241	2,43	0,0250	2,00	0,0901	2,80	0,0109	ZNF160	zinc finger protein 160	NM_198893	Hs.655967
A_23_P108129	2,08	0,0016	1,38	0,0394	1,93	0,0030	ZNF223	zinc finger protein 223	NM_013361	Hs.279840
A_32_P144908	2,62	0,0238	2,72	0,0221	2,71	0,0231	ZNF254	zinc finger protein 254	NM_203282	Hs.434406
A_24_P247512	3,04	0,0009	1,67	0,0857	1,83	0,0553	ZNF283	zinc finger protein 283	AK098175	Hs.652513
A_24_P409670	4,22	0,0000	2,67	0,0016	3,04	0,0004	ZNF283	zinc finger protein 283	AK098175	Hs.652513
A_24_P204043	5,90	0,0016	5,49	0,0027	4,88	0,0087	ZNF318	zinc finger protein 318	NM_014345	Hs.509718
A_23_P38813	2,61	0,0270	2,97	0,0133	3,27	0,0062	ZNF320	zinc finger protein 320	NM_207333	Hs.446907
A_23_P433690	9,01	0,0012					ZNF331	zinc finger protein 331	NM_018555	Hs.185674
A_23_P50376	6,45	0,0000	3,54	0,0088	5,06	0,0002	ZNF331	zinc finger protein 331	NM_018555	Hs.185674
A_23_P258124	3,74	0,0109	3,60	0,0160	3,44	0,0225	ZNF346	zinc finger protein 346	NM_012279	Hs.484259
A_23_P208259	2,36	0,0011	2,23	0,0016	2,41	0,0007	ZNF347	zinc finger protein 347	NM_032584	Hs.467239
A_23_P95736					3,71	0,0752	ZNF429	zinc finger protein 429	NM_001001415	Hs.709598
A_32_P106944	2,82	0,0614	3,03	0,0517	2,77	0,0868	ZNF429	zinc finger protein 429	NM_001001415	Hs.709598
A_23_P5234	2,22	0,0551	2,23	0,0666	2,48	0,0372	ZNF43	zinc finger protein 43	NM_003423	Hs.534365
A_23_P56304	2,76	0,0339	3,07	0,0210	3,16	0,0170	ZNF430	zinc finger protein 430	NM_025189	Hs.466289
A_23_P39441	2,71	0,0373	2,81	0,0361	2,77	0,0412	ZNF431	zinc finger protein 431	NM_133473	Hs.687547
A_24_P470079	3,67	0,0144	3,46	0,0245	3,41	0,0274	ZNF440		AK021474	Hs.418192
A_24_P109854	3,18	0,0006	2,24	0,0137	2,73	0,0024	ZNF468	zinc finger protein 468	NM_199132	Hs.467223
A_24_P16249	2,37	0,0189	1,96	0,0679	2,54	0,0131	ZNF479	zinc finger protein 479	NM_033273	Hs.616660
A_23_P354942	2,54	0,0059	2,44	0,0085	2,56	0,0056	ZNF493	zinc finger protein 493	NM_001076678	Hs.656558
A_24_P332862	1,95	0,0366			1,96	0,0453	ZNF493	zinc finger protein 493	NM_001076678	Hs.656558
A_24_P74329					3,59	0,0576	ZNF493	zinc finger protein 493	NM_001076678	Hs.656558
A_23_P35576	2,88	0,0355	3,02	0,0312	3,02	0,0324	ZNF518A	zinc finger protein 518A	NM_014803	Hs.600823
A_23_P108342	2,88	0,0178	2,76	0,0271	2,56	0,0449	ZNF571	zinc finger protein 571	NM_016536	Hs.590944
A_23_P90223	2,60	0,0172	2,85	0,0096	3,04	0,0057	ZNF585B	zinc finger protein 585B	NM_152279	Hs.631552
A_23_P4850	3,01	0,0019	1,75	0,0918	1,92	0,0586	ZNF625	zinc finger protein 625	NM_145233	Hs.512823
A_32_P182473	2,93	0,0171					ZNF625	zinc finger protein 625	NM_145233	Hs.512823
A_24_P344537	3,54	0,0000	1,94	0,0167	1,98	0,0148	ZNF625-ZNF20	zinc finger protein 625	NM_145233	Hs.512823
A_23_P379142					3,24	0,0914	ZNF626	zinc finger protein 626	NM_145297	Hs.657001

A_32_P102778	2,55	0,0335	2,98	0,0139	2,71	0,0275	ZNF626	zinc finger protein 626	NM_001076675	Hs.657001
A_32_P14669	2,24	0,0476	2,31	0,0487	2,55	0,0273	ZNF66	zinc finger protein 66	AK131420	Hs.658748
A_24_P341292	2,84	0,0065	2,65	0,0122	3,01	0,0041	ZNF665	zinc finger protein 665	NM_024733	Hs.590980
A_23_P101623	4,01	0,0000	1,98	0,0156	2,45	0,0025	ZNF667	zinc finger protein 667	NM_022103	Hs.712574
A_23_P339687	2,62	0,0218	2,74	0,0187	2,85	0,0141	ZNF675	zinc finger protein 675	NM_138330	Hs.264345
A_24_P118862	2,52	0,0657	2,76	0,0512	2,80	0,0485	ZNF678	zinc finger protein 678	NM_178549	Hs.656372
A_23_P337923	2,35	0,0692	2,43	0,0753			ZNF680	zinc finger protein 680	NM_178558	Hs.520886
A_32_P189845	2,51	0,0200	2,40	0,0312	2,61	0,0182	ZNF680	zinc finger protein 680	NM_178558	Hs.520886
A_23_P375517	2,49	0,0628	2,73	0,0480	2,97	0,0291	ZNF681	zinc finger protein 681	NM_138286	Hs.399952
A_24_P91405	3,50	0,0004	2,37	0,0151	2,37	0,0160	ZNF709	zinc finger protein 709	NM_152601	Hs.631623
A_23_P50834	2,26	0,0341	2,00	0,0817	2,38	0,0309	ZNF714	zinc finger protein 714	NM_182515	Hs.466291
A_24_P19268	2,67	0,0197	2,58	0,0291	2,64	0,0253	ZNF714	zinc finger protein 714	NM_182515	Hs.466291
A_24_P204639	2,69	0,0333	2,82	0,0293	2,91	0,0242	ZNF716		ENST0000033142[illegible]	Hs.533121
A_24_P919733	2,59	0,0039	2,18	0,0156	2,49	0,0053	ZNF721	hypothetical protein LOC152719	AK021514	Hs.428360
A_24_P929640	4,73	0,0076	4,26	0,0183	3,31	0,0873	ZNF724P		THC2504595	
A_24_P15965	2,70	0,0181	2,47	0,0365	2,88	0,0133	ZNF726	zinc finger protein 254	NM_203282	Hs.434406
A_32_P212939	3,74	0,0489	4,93	0,0091	4,38	0,0230	ZNF726		XR_015409	Hs.655305
A_32_P46191	2,79	0,0085	2,41	0,0269	2,59	0,0166	ZNF727	zinc finger protein 727	NM_001159522	Hs.640774
A_24_P161696	2,41	0,0193			2,11	0,0513	ZNF729	similar to hCG1773661	ENST0000035882[illegible]	Hs.531629
A_24_P126491			2,48	0,0904			ZNF729	zinc finger protein 729	NM_001242680	Hs.653115
A_24_P161696			2,21	0,0381			ZNF729	similar to hCG1773661	ENST0000035882[illegible]	Hs.531629
A_24_P281801	2,38	0,0221	2,17	0,0449	2,43	0,0222	ZNF732	zinc finger protein 732	NM_001137608	Hs.698668
A_23_P28042	2,35	0,0139	2,00	0,0449	2,29	0,0192	ZNF737	zinc finger protein 737	NM_001159293	
A_32_P68148	2,49	0,0296	2,49	0,0357	2,86	0,0141	ZNF738	zinc finger protein 738	NR_027130	
A_24_P58857	2,74	0,0561			3,15	0,0315	ZNF813	zinc finger protein 813	NM_001004301	Hs.710780
A_32_P3998	2,19	0,0445	1,98	0,0937	2,52	0,0229	ZNF813	zinc finger protein 813	NM_001004301	Hs.710780
A_32_P54531	2,75	0,0102	2,47	0,0249	3,07	0,0044	ZNF826P	zinc finger protein 826	NM_001039884	Hs.631635
A_23_P67702	2,39	0,0163	2,15	0,0362	2,47	0,0146	ZNF85	zinc finger protein 85	NM_003429	Hs.37138
A_24_P153207	4,64	0,0000	2,49	0,0018	2,92	0,0003	ZNF850	zinc finger protein 850 pseudogene	CR627133	Hs.406307
A_23_P124585	2,87	0,0046	2,23	0,0314	2,71	0,0076	ZNF93	zinc finger protein 93	AK096342	Hs.301059
A_23_P135826	3,80	0,0015	2,99	0,0122	3,70	0,0017	ZNF93	zinc finger protein 93	AK096342	Hs.301059
A_23_P158885	2,24	0,0722			2,99	0,0166	ZNF93	zinc finger protein 93	AK096342	Hs.301059
A_24_P160927	2,30	0,0214	2,22	0,0311	2,52	0,0131	ZNF962P		ENST00000305570	
A_24_P194971	2,65	0,0192	2,76	0,0160	3,07	0,0068	ZNF98	zinc finger protein 492	ENST0000035509[illegible]	Hs.232108
A_23_P396570	2,85	0,0871	3,24	0,0586			ZNF99	zinc finger protein 99	BC021822	Hs.568380

PC-UP SIGNATURE

ProbeName	log2FC BPC / BC	adj.P-Val BPC / BC	log2FC TPC / BC	adj.P-Val TPC / BC	log2FC BMPC / BC	adj.P-Val BMPC / BC	Gene Symbol	Gene description	Refseq & others	UNIGENE_ID
A_23_P207650	2,61	0,0802	3,69	0,0122	4,28	0,0033	ACADVL	acyl-CoA dehydrogenase, very long chain	NM_000018	Hs.437178
A_23_P14389	2,30	0,0103	2,20	0,0158	2,27	0,0126	ACIN1	apoptotic chromatin condensation inducer 1	NM_014977	Hs.124490
A_23_P120594	3,39	0,0047	3,50	0,0032	2,91	0,0167	ACSS1	acyl-CoA synthetase short-chain family member 1	NM_032501	Hs.529353
A_23_P44037	5,06	0,0450					ACTG1	actin, gamma 1"	NM_001614	Hs.514581
A_32_P156963	6,79	0,0013	4,16	0,0591	5,03	0,0182	ACTG1	actin gamma 1	NM_001614	Hs.514581
A_32_P175198	2,58	0,0193					ACTG1	actin, gamma 1"	NM_001614	Hs.514581
A_24_P850172	2,89	0,0031	2,18	0,0288	2,36	0,0171	ACTG1P16		XM_926717	
A_23_P148194	4,03	0,0320	3,51	0,0818	4,43	0,0212	ADI1	acireductone dioxygenase 1	NM_018269	Hs.502773
A_23_P46627	4,28	0,0380	4,34	0,0430	4,62	0,0293	ADIPOR1	adiponectin receptor 1	NM_015999	Hs.5298
A_24_P549518	3,17	0,0476	2,94	0,0872	3,35	0,0436	AHCYP3		XR_018505	
A_23_P88963	5,01	0,0020	4,40	0,0060	3,83	0,0197	ALDOA	aldolase A, fructose-bisphosphate	NM_000034	Hs.513490
A_24_P740942	5,93	0,0003	4,79	0,0021	5,52	0,0005	ALDOAP2	aldolase A, fructose-bisphosphate pseudogene 2	M21191	Hs.652473
A_24_P381962	3,69	0,0034	4,46	0,0004	4,12	0,0010	AP1G1	adaptor-related protein complex 1, gamma 1 subunit	NM_001030007	Hs.461253
A_23_P380857	6,66	0,0000	6,89	0,0000	6,35	0,0000	APOL4	apolipoprotein L, 4	NM_030643	Hs.115099
A_23_P70509	2,30	0,0457	2,67	0,0220	2,29	0,0584	ATAT1	alpha tubulin acetyltransferase 1	NM_024909	Hs.654798
A_24_P276932	7,26	0,0042	5,33	0,0424	5,61	0,0314	ATP6V1C2	ATPase, H+ transporting, lysosomal 42kDa, V1 subunit C2	NM_001039362	Hs.580464
A_23_P154643	2,62	0,0674	2,63	0,0835	2,69	0,0769	BMP7	bone morphogenetic protein 7	NM_001719	Hs.473163
A_24_P76868	5,95	0,0000	7,06	0,0000	7,08	0,0000	BMS1P20		X01147	Hs.654512
A_23_P39465	5,17	0,0250	4,58	0,0609	4,67	0,0568	BST2	bone marrow stromal cell antigen 2	NM_004335	Hs.118110
A_23_P67288	3,18	0,0016	2,91	0,0033	2,93	0,0033	CALR	calreticulin	NM_004343	Hs.515162

A_23_P17269	4,74	0,0005	7,81	0,0000	5,18	0,0001	CCDC88A	coiled-coil domain containing 88A	NM_018084	Hs.292925
A_23_P379945	2,71	0,0229	3,23	0,0068	3,32	0,0056	CCDC88C	coiled-coil domain containing 88C	NM_001080414	Hs.525536
A_23_P132454	3,03	0,0686	4,03	0,0153	3,51	0,0400	CCNL1	cyclin L1	NM_020307	Hs.4859
A_24_P337058			2,96	0,0923			CCNL1	cyclin L1	NM_020307	Hs.4859
A_23_P48088	6,62	0,0002	6,34	0,0002	6,27	0,0002	CD27	CD27 molecule	NM_001242	Hs.355307
A_32_P309929	4,97	0,0229	3,95	0,0983	5,27	0,0182	CDK9	cyclin-dependent kinase 9	ENST00000373264	Hs.150423
A_23_P204696	2,78	0,0959	3,60	0,0305	3,72	0,0248	CDKN1B	cyclin-dependent kinase inhibitor 1B (p27, Kip1)	NM_004064	Hs.238990
A_24_P24332	4,76	0,0013	5,95	0,0001	5,03	0,0006	CDV3P1			
A_23_P110504	4,60	0,0000	5,04	0,0000	5,05	0,0000	CLPTM1L	CLPTM1-like	NM_030782	Hs.444673
A_23_P81690	7,20	0,0007	6,47	0,0017	5,09	0,0162	COX7A2	cytochrome c oxidase subunit VIIa polypeptide 2 (liver)	NM_001865	Hs.70312
A_24_P177634	6,84	0,0013	6,37	0,0022	4,81	0,0255	COX7A2P2			
A_23_P41286	3,13	0,0461	3,00	0,0708	3,84	0,0152	CTBP1	C-terminal binding protein 1	NM_001012614	Hs.208597
A_23_P29495	3,14	0,0002	3,48	0,0000	3,20	0,0001	CTNNB1	catenin (cadherin-associated protein), beta 1, 88kDa	NM_001904	Hs.476018
A_23_P14774	3,37	0,0330	3,77	0,0191	4,62	0,0035	CTSH	cathepsin H	NM_004390	Hs.148641
A_23_P106056	7,64	0,0049	5,41	0,0588	5,63	0,0480	DAD1	defender against cell death 1	NM_001344	Hs.82890
A_24_P47870	7,34	0,0071	5,18	0,0769	5,22	0,0770	DAD1	defender against cell death 1	NM_001344	Hs.82890
A_24_P56462	8,05	0,0124					DAD1	defender against cell death 1	NM_001344	Hs.82890
A_23_P149099	4,46	0,0001	3,85	0,0003	3,36	0,0015	DDOST	dolichyl-diphosphooligosaccharide--protein glycosyltransferase subunit (non-	NM_005216	Hs.523145
A_23_P36962	4,89	0,0459	4,66	0,0738	5,39	0,0323	DNAJC3	DnaJ (Hsp40) homolog, subfamily C, member 3"	NM_006260	Hs.656476
A_23_P60376	6,45	0,0006	4,79	0,0095	4,16	0,0277	EDF1	endothelial differentiation-related factor 1	NM_153200	Hs.174050
A_24_P821168	4,25	0,0103	4,59	0,0058	4,29	0,0109	EEF1DP1	eukaryotic translation elongation factor 1 delta (guanine nucleotide exchange	NM_032378	Hs.333388
A_24_P307002	3,25	0,0316	3,66	0,0171	3,30	0,0358	EEF1DP5	eukaryotic translation elongation factor 1 delta (guanine nucleotide exchange	NM_032378	Hs.333388
A_24_P477475	1,99	0,0098	1,79	0,0235	1,67	0,0373	EEF1GP8		XR_018231	
A_24_P87763	4,11	0,0054	4,45	0,0024	4,39	0,0030	EEF2	eukaryotic translation elongation factor 2	NM_001961	Hs.515070
A_24_P169593	3,37	0,0026	2,58	0,0225	2,68	0,0178	EIF2S2P3			
A_24_P206427	4,40	0,0056	4,33	0,0063	3,39	0,0408	EIF3E	eukaryotic translation initiation factor 3, subunit E"	NM_001568	Hs.405590
A_23_P41645	5,74	0,0001	5,24	0,0002	5,47	0,0001	ELL2	elongation factor, RNA polymerase II, 2"	NM_012081	Hs.192221
A_23_P139929	4,72	0,0053	3,55	0,0442	4,31	0,0119	ERP29	endoplasmic reticulum protein 29	NM_006817	Hs.75841
A_24_P204135	2,27	0,0359	2,86	0,0084	2,56	0,0202	FAHD2P1		XR_016023	
A_23_P137751	4,71	0,0022	5,40	0,0004	4,78	0,0016	FAM46C	family with sequence similarity 46, member C	NM_017709	Hs.356216
A_24_P67268	2,32	0,0376	2,51	0,0283	2,25	0,0558	FDPSP4		XR_018717	
A_23_P161596	3,82	0,0505	4,31	0,0302	3,51	0,0991	FKBP2	FK506 binding protein 2, 13kDa"	NM_004470	Hs.227729
A_24_P223124	3,53	0,0768	4,39	0,0290	4,36	0,0311	FNDC3B	fibronectin type III domain containing 3B	NM_022763	Hs.159430
A_23_P50498	5,44	0,0039	5,53	0,0030	5,00	0,0080	FTL	ferritin, light polypeptide"	NM_000146	Hs.433670
A_23_P50504	2,03	0,0144	2,37	0,0041	2,12	0,0119	FTL	ferritin, light polypeptide	NM_000146	Hs.433670
A_32_P155247	3,50	0,0280	4,00	0,0135	2,94	0,0935	FTL	ferritin, light polypeptide	NM_000146	Hs.433670
A_24_P307306	7,08	0,0010	4,38	0,0473	4,39	0,0485	GAPDHP33		ENST00000443168	Hs.641178
A_24_P273666	2,16	0,0098	1,59	0,0769	1,59	0,0798	GNAS	GNAS complex locus	NM_001077489	Hs.125898
A_23_P320478	4,81	0,0405	4,40	0,0805	6,01	0,0111	GOLGA2P10	hypothetical protein FLJ22795	BC065260	Hs.546614
A_23_P3193	3,19	0,0428	3,20	0,0521	3,91	0,0143	GOLGA5	golgin A5	NM_005113	Hs.104320
A_23_P156017	4,97	0,0127	4,89	0,0156	4,13	0,0486	GOLPH3	golgi phosphoprotein 3 (coat-protein)	NM_022130	Hs.408909
A_24_P495367	2,62	0,0034	3,00	0,0006	2,36	0,0080	GRXCR1	glutaredoxin, cysteine rich 1	NM_001080476	Hs.162559
A_24_P928604	5,15	0,0049	4,36	0,0193	4,52	0,0151	hCG_1641703	hCG1641703	ENST00000401937	
A_32_P55934	1,45	0,0084	1,07	0,0686	1,03	0,0837	hCG_21078	hCG21078	CD385156	Hs.708099
A_23_P2582	3,58	0,0019	3,78	0,0009	2,97	0,0099	HDAC7	histone deacetylase 7	NM_015401	Hs.200063
A_23_P428184	3,73	0,0269	4,74	0,0049	3,26	0,0735	HIST1H2AD	histone cluster 1, H2ad	NM_021065	Hs.679229
A_24_P86389	5,05	0,0190	4,97	0,0245	4,29	0,0615	HIST1H2AM	histone cluster 1, H2am	NM_003514	Hs.134999
A_23_P93180	3,20	0,0297	4,50	0,0021	3,24	0,0338	HIST1H2BC	histone cluster 1, H2bc	NM_003526	Hs.658713
A_24_P146211	3,22	0,0402	4,29	0,0060	3,88	0,0146	HIST1H2BD	histone cluster 1, H2bd	NM_021063	Hs.591797
A_32_P152348	3,16	0,0043	4,06	0,0002	3,72	0,0008	HIST1H2BD	histone cluster 1, H2bd	BQ683489	Hs.591797
A_23_P40470	2,92	0,0774	3,97	0,0157	3,38	0,0464	HIST1H2BE	histone cluster 1, H2be"	NM_003523	Hs.534369
A_23_P167997	2,99	0,0457	4,11	0,0056	3,48	0,0222	HIST1H2BG	histone cluster 1, H2bg	NM_003518	Hs.591809
A_23_P111041	3,72	0,0392	4,20	0,0222	4,03	0,0296	HIST1H2BI	histone cluster 1, H2bi	NM_003525	Hs.553506
A_24_P55148	3,85	0,0152	5,03	0,0014	3,95	0,0146	HIST1H2BJ	histone cluster 1, H2bj	NM_021058	Hs.656567
A_24_P3783	3,99	0,0166	4,48	0,0075	4,29	0,0110	HIST1H2BM	histone cluster 1, H2bm	NM_003521	Hs.182432
A_23_P402081	2,40	0,0815	3,18	0,0210	2,57	0,0782	HIST1H2BN	histone cluster 1, H2bn"	NM_003520	Hs.673851
A_24_P544661	3,44	0,0035	4,35	0,0002	3,67	0,0017	HIST2H2BC		BC068044	Hs.595570
A_24_P156911	3,03	0,0218	4,12	0,0938	3,08	0,0230	HIST2H2BE	histone cluster 2, H2be	NM_003528	Hs.2178
A_24_P156911			3,70	0,0050			HIST2H2BE	histone cluster 2, H2be	NM_003528	Hs.2178

A_23_P332992	2,49	0,0273	3,50	0,0018	3,09	0,0067	HIST3H2BB	histone cluster 3, H2bb	NM_175055	Hs.376691
A_24_P524452	2,42	0,0785	3,52	0,0093	2,63	0,0690	HIST3H2BB	histone cluster 3, H2bb	DB010344	Hs.376691
A_23_P408353	3,89	0,0063	3,67	0,0105	3,09	0,0375	HLA-A	major histocompatibility complex, class I, A	NM_002116	Hs.181244
A_24_P912382	3,14	0,0063	2,89	0,0131	2,58	0,0297	HLA-A		ENST0000037680	Hs.181244
A_24_P418044	2,24	0,0077	2,07	0,0149	1,81	0,0381	HLA-J	major histocompatibility complex, class I, J (pseudogene)	NR_024240	Hs.181244
A_23_P396867	5,05	0,0690	6,16	0,0286			HM13	histocompatibility (minor) 13	NM_178582	Hs.373741
A_23_P40347	5,71	0,0000	6,56	0,0000	5,03	0,0002	HM13	histocompatibility (minor) 13	NM_178580	Hs.373741
A_24_P320254			2,01	0,0220	1,75	0,0531	HNRNPH1	heterogeneous nuclear ribonucleoprotein H1 (H)	NM_005520	Hs.604001
A_32_P34149	1,59	0,0475	2,27	0,0041	1,96	0,0151	HNRNPH1	heterogeneous nuclear ribonucleoprotein H1 (H)	NM_005520	Hs.604001
A_23_P2601	4,98	0,0221	4,30	0,0615			HSP90B1	heat shock protein 90kDa beta (Grp94), member 1	NM_003299	Hs.192374
A_24_P150361	3,97	0,0020	3,28	0,0101	3,72	0,0033	HSP90B1	heat shock protein 90kDa beta (Grp94), member 1"	NM_003299	Hs.192374
A_24_P18190	5,38	0,0005	6,39	0,0000	5,15	0,0007	HSPA5	heat shock 70kDa protein 5 (glucose-regulated protein, 78kDa)"	NM_005347	Hs.716396
A_23_P87545	2,44	0,0355	2,63	0,0272	2,68	0,0237	IFITM3	interferon induced transmembrane protein 3	NM_021034	Hs.374650
A_23_P65442	3,38	0,0348	3,95	0,0147	4,51	0,0050	IRF9	interferon regulatory factor 9	NM_006084	Hs.1706
A_24_P379820	6,69	0,0001	4,95	0,0024	6,73	0,0001	ITM2C	integral membrane protein 2C	NM_030926	Hs.111577
A_23_P146431	5,80	0,0000	6,02	0,0000	6,12	0,0000	KIAA0368	KIAA0368	BC021127	Hs.368255
A_23_P149249	2,69	0,0273	2,33	0,0763	2,73	0,0304	KRTCAP2	keratinocyte associated protein 2	NM_173852	Hs.516671
A_24_P40055	2,93	0,0337	3,05	0,0314	3,76	0,0066	LENG8	leukocyte receptor cluster (LRC) member 8	NM_052925	Hs.714385
A_24_P273143	7,00	0,0030	6,16	0,0085	5,21	0,0302	LINC00152	long intergenic non-protein coding RNA 152	NR_024204	Hs.652166
A_23_P9591	6,65	0,0001	5,76	0,0003	5,54	0,0005	LMAN2	lectin, mannose-binding 2"	NM_006816	Hs.75864
A_24_P9285	6,19	0,0010	5,28	0,0041	5,24	0,0049	LMAN2	lectin, mannose-binding 2	NM_006816	Hs.75864
A_24_P350008	3,19	0,0257	2,78	0,0683	2,88	0,0584	LOC100129553	hypothetical LOC100129553	ENST00000442083	
A_24_P221615	3,44	0,0012	5,69	0,0000	5,35	0,0000	LOC100129631	hypothetical protein LOC100129631		
A_32_P793319	2,68	0,0059	3,58	0,0002	3,44	0,0004	LOC100131744	hypothetical protein LOC100131744	AK098749	Hs.652768
A_24_P203418	3,47	0,0001	5,02	0,0000	5,10	0,0000	LOC100131845	similar to hCG1742309	ENST0000061737	Hs.547094
A_23_P326987	3,26	0,0703	3,99	0,0285	4,26	0,0186	LOC100190939	hypothetical LOC100190939	AF318337	
A_24_P401090	4,47	0,0005	5,49	0,0000	4,40	0,0005	LOC400750	hypothetical gene supported by AF216292; NM_005347	ENST00000442006	
A_23_P63953	2,19	0,0066	1,75	0,0362	1,84	0,0269	LOC646119	similar to hCG2040247	XM_929084	
A_24_P187197	4,13	0,0174	4,11	0,0210	5,09	0,0035	LOC652005	similar to melanoma-associated chondroitin sulfate proteoglycan 4	BX648930	Hs.639860
A_24_P627503	1,85	0,0113	3,57	0,0000	2,96	0,0001	LOC652494	similar to Ig heavy chain V-III region VH26 precursor	ENST0000060366	Hs.510635
A_24_P889462	2,39	0,0001	2,76	0,0000	2,95	0,0000	LOC652494	similar to Ig heavy chain V-III region VH26 precursor	L38427	Hs.510635
A_24_P256037	3,16	0,0060	2,45	0,0406	2,62	0,0268	LOC654170	similar to hCG1643342	ENST00000479473	
A_24_P861442	5,35	0,0059	4,49	0,0237	4,75	0,0163	LOC728428	hypothetical LOC728428	XR_015830	Hs.647679
A_24_P409661	6,77	0,0005	4,96	0,0088	5,71	0,0025	LOC729768	hypothetical LOC729768	ENST00000448345	
A_23_P132793	5,43	0,0000	4,74	0,0001	3,64	0,0032	MANF	mesencephalic astrocyte-derived neurotrophic factor	NM_006010	Hs.436446
A_24_P336754	3,69	0,0348	4,97	0,0041	4,79	0,0063	MCL1	myeloid cell leukemia sequence 1 (BCL2-related)	NM_021960	Hs.713518
A_24_P336759			4,13	0,0305	3,42	0,0926	MCL1	myeloid cell leukemia 1	NM_021960	Hs.713518
A_23_P415021	4,48	0,0719					METTL7A	methyltransferase like 7A	NM_014033	Hs.716437
A_24_P941773	4,77	0,0117	3,91	0,0484	3,64	0,0752	METTL7A	methyltransferase like 7A	NM_014033	Hs.716437
A_24_P6921	7,19	0,0026	6,23	0,0086	5,33	0,0282	MIR4435-1HG	hypothetical LOC541471	ENST0000030860	Hs.560805
A_23_P84596	7,03	0,0000	7,64	0,0000	6,79	0,0000	MZB1	marginal zone B and B1 cell-specific protein	NM_016459	Hs.409563
A_23_P152804	6,19	0,0045	4,15	0,0733	4,05	0,0866	NME1	NME/NM23 nucleoside diphosphate kinase 1	NM_198175	Hs.463456
A_23_P398005	5,08	0,0716					NME2P1	non-metastatic cells 2, protein (NM23B) expressed in, pseudogene 1"	NR_001577	
A_32_P39324	5,49	0,0035	4,29	0,0245	3,91	0,0449	NME2P1		CB993898	Hs.577804
A_24_P16340	3,25	0,0050	2,70	0,0222	2,36	0,0523	NP1207855	similar to acidic ribosomal phosphoprotein P0	ENST0000044811	Hs.647767
A_24_P335263	4,48	0,0034	5,37	0,0004	3,64	0,0186	NUDT4	nudix (nucleoside diphosphate linked moiety X)-type motif 4	NM_199040	Hs.506325
A_24_P50753	4,40	0,0037	5,33	0,0004	3,30	0,0343	NUDT4	nudix (nucleoside diphosphate linked moiety X)-type motif 4	NM_199040	Hs.506325
A_23_P129829	3,54	0,0043	3,24	0,0089	3,84	0,0019	ORMDL3	ORMDL sphingolipid biosynthesis regulator 3	NM_139280	Hs.514151
A_23_P107412	7,06	0,0000	7,32	0,0000	6,39	0,0000	P4HB	prolyl 4-hydroxylase, beta polypeptide	NM_000918	Hs.464336
A_24_P213684	7,00	0,0007	5,67	0,0048	6,66	0,0010	P4HB	prolyl 4-hydroxylase, beta polypeptide"	AB062434	Hs.464336
A_23_P51718	2,59	0,0638	3,13	0,0274	2,66	0,0735	PABPC4	poly(A) binding protein, cytoplasmic 4 (inducible form)"	NM_003819	Hs.169900
A_23_P210001	2,41	0,0627	3,55	0,0052	3,20	0,0136	PAX8	paired box 8	NM_003466	Hs.469728
A_23_P42802	6,48	0,0065	5,40	0,0280	5,97	0,0139	PDIA4	protein disulfide isomerase family A, member 4	NM_004911	Hs.93659
A_23_P167040	5,03	0,0235	4,34	0,0669	4,82	0,0376	PDIA5	protein disulfide isomerase family A, member 5	NM_006810	Hs.477352
A_23_P13604	6,48	0,0016	5,35	0,0086	5,27	0,0101	PEBP1	phosphatidylethanolamine binding protein 1	NM_002567	Hs.713526
A_23_P345118	6,01	0,0001	7,31	0,0000	6,72	0,0000	PIM1	Pim-1 proto-oncogene, serine/threonine kinase	NM_002648	Hs.81170
A_24_P379104	4,37	0,0000	4,88	0,0000	4,94	0,0000	PIM2	Pim-2 proto-oncogene, serine/threonine kinase	NM_006875	Hs.716596
A_23_P312920	4,80	0,0244	3,94	0,0891	4,90	0,0256	POU2AF1	POU class 2 associating factor 1	NM_006235	Hs.654525
A_24_P304723	7,32	0,0000	6,59	0,0000	6,05	0,0002	PPIB	peptidylprolyl isomerase B (cyclophilin B)	NM_000942	Hs.434937

A_23_P91114	5,80	0,0026	6,56	0,0005	3,59	0,0820	PREB	prolactin regulatory element binding	NM_013388	Hs.279784
A_32_P364462	1,68	0,0907	2,08	0,0369	2,02	0,0457	RASIP1	Ras interacting protein 1	NM_017805	Hs.233955
A_32_P131377	5,53	0,0580	5,34	0,0855	5,23	0,0987	REEP5	receptor accessory protein 5	NM_005669	Hs.429608
A_23_P97141	3,52	0,0650	7,63	0,0001	4,93	0,0093	RGS1	regulator of G-protein signaling 1	NM_002922	Hs.75256
A_24_P375237	3,60	0,0031	3,22	0,0080	2,61	0,0375	RPL12	ribosomal protein L12	NM_000976	Hs.408054
A_24_P332471	2,72	0,0001	2,67	0,0001	2,23	0,0012	RPL12P18	similar to ribosomal protein L12	ENST0000043717	Hs.646786
A_24_P40757	4,25	0,0080	3,92	0,0160	2,95	0,0935	RPL12P8		XM_928198	
A_24_P142223	4,48	0,0046	4,42	0,0049	4,23	0,0076	RPL13	ribosomal protein L13	NM_033251	Hs.410817
A_24_P142228	4,47	0,0105	4,37	0,0140	4,21	0,0189	RPL13	ribosomal protein L13	NM_033251	Hs.410817
A_23_P90143	1,01	0,0638					RPL13A	ribosomal protein L13a	NM_012423	Hs.523185
A_24_P272061	1,97	0,0567	2,04	0,0595			RPL13A	ribosomal protein L13a	NM_012423	Hs.523185
A_32_P92295	3,46	0,0367	3,07	0,0854	3,22	0,0693	RPL13A	ribosomal protein L13a	NM_012423	Hs.523185
A_24_P289884	2,97	0,0192	3,34	0,0089	3,19	0,0134	RPL13P			
A_24_P238426	4,88	0,0055	4,77	0,0066	4,52	0,0109	RPL13P6			
A_24_P238427	3,24	0,0047	3,04	0,0081	2,94	0,0108	RPL13P6			
A_32_P193288	5,20	0,0014	4,28	0,0078	4,36	0,0068	RPL18A	ribosomal protein L18a	NM_000980	Hs.337766
A_24_P178654	4,69	0,0003	3,95	0,0015	3,84	0,0022	RPL18AP15	similar to mCG14783	ENST00000430258	
A_23_P90383	3,86	0,0070	3,17	0,0315	3,40	0,0202	RPL18AP3	ribosomal protein L18a	NM_000980	Hs.337766
A_24_P366768	5,35	0,0003	4,78	0,0009	4,49	0,0019	RPL18AP8		XR_018308	
A_23_P256933	2,15	0,0539					RPL24	ribosomal protein L24	NM_000986	Hs.477028
A_32_P100974	4,58	0,0108	4,10	0,0272	3,85	0,0408	RPL24	ribosomal protein L24	NM_000986	Hs.477028
A_23_P203577	3,31	0,0004	2,81	0,0020	2,71	0,0031	RPL27A	ribosomal protein L27a	NM_000990	Hs.523463
A_23_P68942	3,85	0,0004	3,69	0,0005	4,05	0,0002	RPL3	ribosomal protein L3	NM_000967	Hs.119598
A_23_P112429	2,31	0,0240	4,07	0,0017	4,53	0,0006	RPL35	ribosomal protein L35	NM_007209	Hs.182825
A_24_P403403	2,27	0,0186					RPL35	ribosomal protein L35	NM_007209	Hs.182825
A_32_P72940	5,31	0,0001					RPL35	ribosomal protein L35	NM_007209	Hs.182825
A_24_P367063	2,10	0,0018	2,04	0,0021	2,37	0,0004	RPL3P12			
A_24_P280933	2,43	0,0005	2,45	0,0003	2,80	0,0001	RPL3P3		XR_018187	
A_23_P31654	4,31	0,0025	3,98	0,0046	3,69	0,0093	RPL8	ribosomal protein L8	NM_000973	Hs.178551
A_23_P204472	4,48	0,0000	3,46	0,0009	3,38	0,0010	RPLP0	ribosomal protein, large, P0	NM_053275	Hs.546285
A_32_P116881	4,36	0,0002	3,49	0,0021	3,03	0,0082	RPLP0	ribosomal protein, large, P0"	NM_053275	Hs.546285
A_24_P358010	3,63	0,0005	3,29	0,0010	3,03	0,0026	RPLP1P6		BG213931	Hs.637971
A_23_P209152	2,88	0,0258					RPS15	ribosomal protein S15	NM_001018	Hs.406683
A_24_P225010	4,53	0,0047	3,47	0,0353	3,70	0,0237	RPS15	ribosomal protein S15	NM_001018	Hs.406683
A_23_P208850	3,15	0,0060	2,54	0,0314	2,33	0,0541	RPS16	ribosomal protein S16	NM_001020	Hs.397609
A_23_P142475	6,52	0,0117	6,16	0,0196	5,40	0,0465	RPS28	ribosomal protein S28	NM_001031	Hs.322473
A_23_P157196	1,97	0,0142	1,62	0,0564	1,64	0,0521	RPS2P32	ribosomal protein S2 pseudogene 32	NR_026676	Hs.367935
A_24_P92744	2,11	0,0304	1,94	0,0586	1,93	0,0636	RPS2P52			
A_24_P50707	1,59	0,0369	1,61	0,0419	1,41	0,0921	RPS4XP19		XR_019448	
A_24_P477051	1,91	0,0138	1,56	0,0567	1,56	0,0598	RPS4XP21	similar to 40S ribosomal protein S4, X isoform	ENST0000060381	Hs.646361
A_23_P46182	3,94	0,0008	2,95	0,0107	2,75	0,0192	RPS8	ribosomal protein S8	NM_001012	Hs.512675
A_32_P179295	4,48	0,0626					RPS9	ribosomal protein S9	BC020462	Hs.467284
A_32_P22078	4,45	0,0148	4,06	0,0310	3,68	0,0583	RPS9	ribosomal protein S9	NM_001013	Hs.546288
A_32_P223456	5,02	0,0357	4,19	0,0167	4,49	0,0826	RPS9	ribosomal protein S9	BC020462	Hs.467284
A_23_P120566	5,04	0,0037			5,66	0,0011	RRBP1	ribosome binding protein 1	NM_001042576	Hs.472213
A_23_P6344	4,33	0,0115	3,76	0,0334	3,68	0,0400	SDF2L1	stromal cell-derived factor 2-like 1	NM_022044	Hs.303116
A_23_P208009	8,26	0,0000	8,13	0,0000	7,73	0,0000	SEC11C	SEC11 homolog C (S. cerevisiae)	NM_033280	Hs.45107
A_23_P206960	3,44	0,0955			4,24	0,0425	SEC14L1	SEC14-like 1 (S. cerevisiae)	NM_003003	Hs.464184
A_24_P237766	6,07	0,0029	6,38	0,0014	5,43	0,0074	SEC14L1	SEC14-like 1 (S. cerevisiae)	AK130317	Hs.464184
A_23_P412392	4,41	0,0099	4,04	0,0210	4,68	0,0066	SEC22B	SEC22 vesicle trafficking protein homolog B (S. cerevisiae) (gene/pseudoger	NM_004892	Hs.632438
A_23_P212458	5,37	0,0026	5,32	0,0024	4,10	0,0231	SEC61A1	Sec61 alpha 1 subunit (S. cerevisiae)	NM_013336	Hs.518236
A_24_P353638	4,66	0,0074	5,06	0,0034	5,48	0,0017	SLAMF7	SLAM family member 7	NM_021181	Hs.517265
A_23_P55998	4,19	0,0423	5,20	0,0122	4,90	0,0194	SLC1A5	solute carrier family 1 (neutral amino acid transporter), member 5	NM_005628	Hs.631582
A_23_P70571	7,19	0,0000	7,27	0,0000	7,12	0,0000	SLC39A7	solute carrier family 39 (zinc transporter), member 7	NM_006979	Hs.631995
A_23_P18317	4,04	0,0000	4,72	0,0000	4,72	0,0000	SLC41A3	solute carrier family 41, member 3	NM_017836	Hs.573007
A_23_P216630	5,92	0,0001	4,34	0,0033	2,82	0,0811	SLC44A1	solute carrier family 44 (choline transporter), member 1	NM_080546	Hs.573495
A_24_P335620	3,92	0,0367	6,52	0,0005	5,53	0,0032	SLC7A5	solute carrier family 7 (amino acid transporter light chain, L system), member	NM_003486	Hs.513797
A_32_P3600	4,96	0,0134	4,60	0,0258	4,88	0,0173	SMARCC2		THC2587763	
A_23_P42829	7,91	0,0019	6,28	0,0136	6,97	0,0057	SND1	staphylococcal nuclease and tudor domain containing 1	NM_014390	Hs.122523

A_23_P118791	3,81	0,0103	7,26	0,0000	7,34	0,0000	SNORD118	transmembrane protein 107	NM_032354	Hs.513933
A_23_P24594	3,39	0,0010	3,02	0,0025	2,20	0,0354	SNORD14E	heat shock 70kDa protein 8	NM_006597	Hs.702021
A_24_P287129	3,43	0,0006	3,16	0,0011	2,28	0,0220	SNORD14E	heat shock 70kDa protein 8	NM_006597	Hs.702021
A_32_P13728	3,85	0,0008	3,42	0,0024	2,78	0,0162	SNORD14E	heat shock 70kDa protein 8	NM_006597	Hs.702021
A_32_P134634	4,08	0,0055	5,12	0,0005	5,53	0,0002	SNORD3D		THC2530077	Hs.658815
A_23_P29630	6,71	0,0008	5,74	0,0033	7,35	0,0002	SPCS1	signal peptidase complex subunit 1 homolog (S. cerevisiae)	NM_014041	Hs.11125
A_23_P86653	6,40	0,0097	5,15	0,0468	5,16	0,0468	SRGN	serglycin	NM_002727	Hs.1908
A_23_P80773	6,55	0,0001	6,89	0,0000	5,19	0,0015	SRPRB	signal recognition particle receptor, B subunit	NM_021203	Hs.12152
A_23_P126474	7,26	0,0030	6,68	0,0056	5,55	0,0253	SSR2	signal sequence receptor, beta (translocon-associated protein beta)"	NM_003145	Hs.74564
A_23_P155229	6,34	0,0070	5,27	0,0293	4,56	0,0714	SSR3	signal sequence receptor, gamma (translocon-associated protein gamma)	NM_007107	Hs.518346
A_24_P319942	6,14	0,0028	6,21	0,0021	4,65	0,0257	SSR3	signal sequence receptor, gamma (translocon-associated protein gamma)	NM_007107	Hs.518346
A_23_P259172	5,77	0,0001	7,38	0,0000	6,66	0,0000	SSR4	signal sequence receptor, delta	NM_006280	Hs.409223
A_32_P2333	5,84	0,0007	3,87	0,0260	4,76	0,0051	SUB1P3	SUB1 homolog (S. cerevisiae)	NM_006713	Hs.229641
A_32_P11477	6,46	0,0131	5,11	0,0651	5,10	0,0683	SYVN1	Finkel-Biskis-Reilly murine sarcoma virus (FBR-MuSV) ubiquitously expresse	NM_001997	Hs.387208
A_23_P204702	3,94	0,0672	4,35	0,0499	4,01	0,0813	TMBIM6	transmembrane BAX inhibitor motif containing 6	NM_003217	Hs.708025
A_23_P70127	2,92	0,0371	2,75	0,0636	3,02	0,0383	TMED9	transmembrane emp24 protein transport domain containing 9	NM_017510	Hs.279929
A_23_P111288	3,99	0,0026	2,95	0,0285	3,48	0,0081	TMEM14C	transmembrane protein 14C	NM_016462	Hs.519557
A_23_P115313	2,92	0,0458	3,28	0,0280	3,37	0,0231	TOR3A	torsin family 3, member A	NM_022371	Hs.584957
A_23_P168882	4,62	0,0058	3,49	0,0457	4,48	0,0077	TP53INP1	tumor protein p53 inducible nuclear protein 1	NM_033285	Hs.682015
A_24_P255252	1,36	0,0086	1,72	0,0008	1,35	0,0100	TPT1P5			
A_23_P123503	5,23	0,0619	6,19	0,0294	5,89	0,0410	TRIB1	tribbles homolog 1 (Drosophila)	NM_025195	Hs.444947
A_24_P252497	5,85	0,0036	7,50	0,0002	6,30	0,0016	TRIB1	tribbles pseudokinase 1	NM_025195	Hs.444947
A_23_P154065	2,69	0,0579	3,28	0,0220	2,95	0,0437	TUBA4A	tubulin, alpha 4a	NM_006000	Hs.75318
A_23_P154070	2,45	0,0785					TUBA4A	tubulin, alpha 4a"	NM_006000	Hs.75318
A_23_P84448			3,51	0,0521			TUBA4A	tubulin, alpha 4a	NM_006000	Hs.75318
A_23_P14939	8,02	0,0013	8,36	0,0006	6,81	0,0057	TXNDC11	thioredoxin domain containing 11	NM_015914	Hs.313847
A_24_P107897	6,56	0,0000	7,49	0,0000	6,80	0,0000	TXNDC11	thioredoxin domain containing 11	ENST0000035695	Hs.313847
A_23_P168229	7,27	0,0003	6,40	0,0010	6,71	0,0006	TXNDC5	thioredoxin domain containing 5 (endoplasmic reticulum)	NM_030810	Hs.150837
A_23_P18304	4,04	0,0656	4,61	0,0406	5,07	0,0220	UBA5	ubiquitin-like modifier activating enzyme 5	NM_198329	Hs.170737
A_23_P56188	2,57	0,0163					UBA52	ubiquitin A-52 residue ribosomal protein fusion product 1	NM_001033930	Hs.5308
A_23_P56195	1,41	0,0232	1,98	0,0013	1,62	0,0096	UBA52	ubiquitin A-52 residue ribosomal protein fusion product 1	NM_001033930	Hs.5308
A_24_P173325	1,84	0,0420					UBA52	ubiquitin A-52 residue ribosomal protein fusion product 1	NM_001033930	Hs.5308
A_24_P194714	4,14	0,0044	5,19	0,0003	4,46	0,0021	UBALD2	UBA-like domain containing 2	NM_182565	Hs.709534
A_23_P27215	2,45	0,0005	3,00	0,0000	2,58	0,0002	UBB	ubiquitin B	NM_018955	Hs.356190
A_24_P113109	1,33	0,0192	2,03	0,0003	1,75	0,0021	UBB	ubiquitin B	NM_018955	Hs.356190
A_23_P329740	3,41	0,0747	5,56	0,0028	3,54	0,0821	UBC	ubiquitin C	NM_021009	Hs.520348
A_24_P266880	2,46	0,0087	3,24	0,0003	2,75	0,0021	UBC	ubiquitin C	NM_021009	Hs.520348
A_24_P681301	1,58	0,0229	2,04	0,0032	1,70	0,0162	UBC	ubiquitin C	NM_021009	Hs.520348
A_24_P889720	1,81	0,0048	2,49	0,0001	2,12	0,0009	UBC	ubiquitin C	NM_021009	Hs.520348
A_23_P103905	5,82	0,0012	4,66	0,0088	4,85	0,0063	UFC1	ubiquitin-fold modifier conjugating enzyme 1	NM_016406	Hs.301412
A_23_P26084	5,58	0,0000	6,11	0,0000	6,33	0,0000	VIMP	selenoprotein S	NM_018445	Hs.32148
A_24_P912889	5,85	0,0671					VIMP	selenoprotein S	NM_203472	Hs.32148
A_32_P9753	4,42	0,0437	4,70	0,0365	5,32	0,0166	VMP1	transmembrane protein 49	BC024020	Hs.708260
A_23_P168211	4,82	0,0037	4,34	0,0087	4,77	0,0039	VOPP1	vesicular, overexpressed in cancer, prosurvival protein 1	NM_030796	Hs.488307
A_23_P371155	6,08	0,0042	6,09	0,0037	4,94	0,0225	WTAP	Wilms tumor 1 associated protein	NM_004906	Hs.446091
A_23_P120845	4,15	0,0011	5,22	0,0000	4,10	0,0011	XBP1	X-box binding protein 1	NM_005080	Hs.437638
A_24_P100228	4,14	0,0339	5,00	0,0109	4,70	0,0182	XBP1	X-box binding protein 1	NM_001079539	Hs.437638
A_23_P254888	2,38	0,0316	2,82	0,0117	2,12	0,0771	ZYX	zyxin	NM_003461	Hs.490415
