# Supplementary material for: The transcriptional profiling of human in vivo-generated plasma cells identifies selective imbalances in monoclonal gammopathies
Source: PLoS One. 2017 Aug 17;12(8):e0183264. doi: 10.1371/journal.pone.0183264 (PMC5560601; doi:10.1371/journal.pone.0183264)
Supplement: S2 Table — The table shows the significant GO terms (P < 0.05, DAVID) with associated P-values, fold enrichment, genes and the manual categories used in the figures. (PDF) [file pone.0183264.s004.pdf]

Supplementary Table 2. Gene Ontology analysis. The table shows the significant GO terms (*P* < 0.05, DAVID) with associated P-values, fold enrichment, genes and the manual categories used in the figures.

PC-down signature

Category	GO term	*P* -value	Benjamini	Fold Enrichment	Genes
	GO:0006357~regulation of transcription from RNA polymerase II promoter	1,6E-02	7,0E-01	1,7	ATF7IP, CIITA, GLIS3, IL6, SP100, ELF4, HDAC10, SMAD3, PAX5, PAX4, NR4A3, HLTF, ZNF254, SKAP1, FOXP1, HDAC4, TRAK2, CSRNP3, SP2, ATXN7, SUPT4H1, NFIC
	GO:0010628~positive regulation of gene expression	4,8E-02	9,2E-01	1,7	ATF7IP, CIITA, GLIS3, IL6, SP100, ELF4, SMAD3, NR4A3, SKAP1, HDAC4, MAPK1, TNFSF13B, REL, CSRNP3, ATXN7, SUPT4H1, NFIC
	GO:0045893~positive regulation of transcription, DNA-dependent	4,0E-02	9,2E-01	1,8	ATF7IP, CIITA, GLIS3, IL6, SP100, ELF4, SMAD3, NR4A3, SKAP1, HDAC4, REL, CSRNP3, ATXN7, SUPT4H1, NFIC
	GO:0051254~positive regulation of RNA metabolic process	4,3E-02	9,1E-01	1,8	ATF7IP, CIITA, GLIS3, IL6, SP100, ELF4, SMAD3, NR4A3, SKAP1, HDAC4, REL, CSRNP3, ATXN7, SUPT4H1, NFIC
	GO:0010605~negative regulation of macromolecule metabolic process	1,8E-02	7,1E-01	1,7	ATF7IP, CIITA, GLIS3, IL6, SP100, HDAC10, SMAD3, ZNF675, PAX5, PAX4, ZNF254, FOXP1, HDAC4, NAB2, PER1, PEX14, PSME3, INPP5D, SUPT4H1, NFIC, CLN8, BUB3
	GO:0010558~negative regulation of macromolecule biosynthetic process	7,4E-03	5,7E-01	2,0	ATF7IP, CIITA, GLIS3, IL6, SP100, HDAC10, SMAD3, ZNF675, PAX5, PAX4, ZNF254, FOXP1, HDAC4, NAB2, PEX14, PER1, INPP5D, SUPT4H1, NFIC
	GO:0031327~negative regulation of cellular biosynthetic process	9,5E-03	6,1E-01	1,9	ATF7IP, CIITA, GLIS3, IL6, SP100, HDAC10, SMAD3, ZNF675, PAX5, PAX4, ZNF254, FOXP1, HDAC4, NAB2, PEX14, PER1, INPP5D, SUPT4H1, NFIC
	GO:0009890~negative regulation of biosynthetic process	1,2E-02	6,4E-01	1,9	ATF7IP, CIITA, GLIS3, IL6, SP100, HDAC10, SMAD3, ZNF675, PAX5, PAX4, ZNF254, FOXP1, HDAC4, NAB2, PEX14, PER1, INPP5D, SUPT4H1, NFIC
	GO:0000122~negative regulation of transcription from RNA polymerase II promoter	2,5E-03	4,3E-01	2,8	ATF7IP, CIITA, GLIS3, SP100, HDAC10, PAX5, SMAD3, PAX4, ZNF254, FOXP1, HDAC4, SUPT4H1, NFIC
Transcription	GO:0016481~negative regulation of transcription	6,8E-03	5,6E-01	2,1	ATF7IP, CIITA, GLIS3, SP100, HDAC10, SMAD3, ZNF675, PAX5, PAX4, ZNF254, FOXP1, HDAC4, NAB2, PEX14, PER1, SUPT4H1, NFIC
	GO:0010629~negative regulation of gene expression	1,5E-02	7,1E-01	1,9	ATF7IP, CIITA, GLIS3, SP100, HDAC10, SMAD3, ZNF675, PAX5, PAX4, ZNF254, FOXP1, HDAC4, NAB2, PEX14, PER1, SUPT4H1, NFIC
	GO:0045934~negative regulation of nucleobase, nucleoside, nucleotide and nucleic acid metabolic process	1,8E-02	7,2E-01	1,9	ATF7IP, CIITA, GLIS3, SP100, HDAC10, SMAD3, ZNF675, PAX5, PAX4, ZNF254, FOXP1, HDAC4, NAB2, PEX14, PER1, SUPT4H1, NFIC
	GO:0051172~negative regulation of nitrogen compound metabolic process	2,0E-02	7,3E-01	1,9	ATF7IP, CIITA, GLIS3, SP100, HDAC10, SMAD3, ZNF675, PAX5, PAX4, ZNF254, FOXP1, HDAC4, NAB2, PEX14, PER1, SUPT4H1, NFIC
	GO:0045892~negative regulation of transcription, DNA-dependent	3,9E-03	4,3E-01	2,4	ATF7IP, CIITA, GLIS3, SP100, HDAC10, SMAD3, ZNF675, PAX5, PAX4, ZNF254, FOXP1, HDAC4, PEX14, SUPT4H1, NFIC
	GO:0051253~negative regulation of RNA metabolic process	4,6E-03	4,5E-01	2,4	ATF7IP, CIITA, GLIS3, SP100, HDAC10, SMAD3, ZNF675, PAX5, PAX4, ZNF254, FOXP1, HDAC4, PEX14, SUPT4H1, NFIC
	GO:0051252~regulation of RNA metabolic process	1,3E-11	1,0E-08	2,3	ZNF85, ELF4, ZNF675, PAX5, ZKSCAN1, PAX4, ZNF347, ZNF254, SKAP1, RNF141, ZNF729, TRAK2, ZNF738, ZNF727, ZNF726, ZNF680, ZNF737, ZNF732, ZNF724P, ZNF493, ZNF43, ATF7IP, CIITA, ZNF93, L3MBTL4, ZNF283, SP100, ZNF479, ZNF440, HDAC10, ZNF813, MBNL1, HLTF, ZNF134, ZNF714, ZNF716, ZNF431, ZNF98, ZNF99, GLIS3, ZNF430, ZNF468, ZNF331, TSC22D2, REL, ZNF709, ZNF429, ZNF223, TFDP2, PER1, PEX14, SUPT4H1, IL6, ZNF66, ZNF667, ZNF160, SMAD3, NR4A3, ZNF320, ZNF626, TRIM22, FOXP1, ZNF585B, NOTCH2, HDAC4, CSRNP3, DLX6, SP2, ATXN7, ZNF117, NFIC, ZNF571
	GO:0006355~regulation of transcription, DNA-dependent	1,3E-11	7,1E-09	2,3	ZNF85, ELF4, ZNF675, PAX5, ZKSCAN1, PAX4, ZNF347, ZNF254, SKAP1, RNF141, ZNF729, TRAK2, ZNF738, ZNF727, ZNF726, ZNF680, ZNF737, ZNF732, ZNF724P, ZNF493, ZNF43, ATF7IP, CIITA, ZNF93, ZNF283, L3MBTL4, SP100, ZNF479, ZNF440, HDAC10, ZNF813, HLTF, ZNF134, ZNF714, ZNF716, ZNF431, ZNF98, ZNF99, GLIS3, ZNF430, ZNF468, ZNF331, TSC22D2, REL, ZNF709, ZNF429, ZNF223, TFDP2, PER1, PEX14, SUPT4H1, IL6, ZNF66, ZNF667, ZNF160, SMAD3, NR4A3, ZNF320, ZNF626, TRIM22, FOXP1, ZNF585B, NOTCH2, HDAC4, CSRNP3, DLX6, SP2, ATXN7, ZNF117, NFIC, ZNF571
	GO:0045449~regulation of transcription	9,9E-13	1,6E-09	2,0	ZNF724P, ZNF493, ZNF43, ATF7IP, CIITA, ZNF93, SNAPC5, L3MBTL4, ZNF283, SP100, ZNF440, ZNF479, SNAPC3, HDAC10, ZNF813, HLTF, MAPK1, CCR6, ZNF134, ZNF714, NAB2, PARP14, ZNF716, ZNF431, ZNF98, ZNF99, GLIS3, ZNF430, ZNF468, SCML1, ZNF331, TSC22D2, REL, ZNF709, ZNF429, ZNF223, TFDP2, PER1, PEX14, SUPT4H1, IL6, ZNF625, L3MBTL3, ZNF66, ZNF160, ZNF667, SMAD3, AFF3, ZFP3, NR4A3, ZNF320, ZNF626, TRIM22, FOXP1, ABCG1, ZNF585B, ATF7IP2, ZNF665, NOTCH2, HDAC4, CSRNP3, DLX6, SP2, ATXN7, ZNF318, ZNF117, NFIC, ZNF571
	GO:0006350~transcription	6,4E-10	2,5E-07	2,0	ZNF85, ELF4, ZNF675, PAX5, ZKSCAN1, PAX4, ZNF347, ZNF518A, ZNF254, ZNF678, RCBTB1, MED28, ZNF738, ZNF727, ZNF680, PRMT5, ZNF681, ZNF732, ZNF721, ZNF493, ZNF43, ATF7IP, CIITA, SNAPC5, ZNF93, ZNF283, ZNF440, ZNF479, SNAPC3, HDAC10, ZNF813, HLTF, CCR6, ZNF134, ZNF714, NAB2, PARP14, ZNF431, ZNF98, ZNF99, GLIS3, ZNF430, ZNF468, SCML1, ZNF331, REL, ZNF709, ZNF429, ZNF223, TFDP2, PER1, SUPT4H1, ZNF625, ZNF66, ZNF667, ZNF160, SMAD3, AFF3, ZFP3, NR4A3, ZNF320, ZNF626, FOXP1, ZNF585B, ATF7IP2, ZNF665, NOTCH2, HDAC4, CSRNP3, SP2, ATXN7, ZNF318, ZNF117, NFIC, ZNF571
	GO:0006959~humoral immune response	2,5E-03	3,9E-01	5,1	CD83, CR1, IL6, CCR6, C6, C5, PAX5
	GO:0002684~positive regulation of immune system process	3,1E-03	4,2E-01	2,9	CD83, MAPK1, CR1, IL6, TNFSF13B, C6, FCER2, IL4R, C5, INPP5D, SKAP1, FOXP1
	GO:0034341~response to interferon-gamma	1,2E-02	6,5E-01	17,1	CIITA, KYNU, SP100
	GO:0002460~adaptive immune response based on somatic recombination of immune receptors built from Ig superfamily domains	4,6E-02	9,1E-01	3,7	CR1, C6, C5, INPP5D, GNL1
Immune	GO:0002250~adaptive immune response	4,6E-02	9,1E-01	3,7	CR1, C6, C5, INPP5D, GNL1
response	GO:0002443~leukocyte mediated immunity	3,9E-03	4,6E-01	4,6	CR1, IL6, LYST, C6, C5, INPP5D, GNL1
	GO:0002252~immune effector process	2,4E-03	4,7E-01	3,8	CR1, IL6, TNFSF13B, LYST, C6, C5, INPP5D, GNL1, FOXP1
	GO:0002449~lymphocyte mediated immunity	7,5E-03	5,4E-01	4,9	CR1, LYST, C6, C5, INPP5D, GNL1
	GO:0006955~immune response	7,2E-05	2,2E-02	2,3	HLA-DQB1, MAVS, KYNU, C6, C5, PAX5, HLA-DMB, SKAP1, CD96, IL4R, CNR2, MS4A1, INPP5D, GNL1, CIITA, IL6, CR1, SP100, SMAD3, TRIM22, FOXP1, CD83, CCR6, TNFSF13B, PPBP, SP2, LYST, CD300LF
	GO:0050778~positive regulation of immune response	4,2E-02	9,2E-01	2,8	MAPK1, CR1, TNFSF13B, C6, FCER2, C5, SKAP1
	GO:0045087~innate immune response	1,1E-02	6,3E-01	3,3	MAVS, CIITA, CR1, KYNU, SP100, LYST, C6, C5
Golgi transport	GO:0006891~intra-Golgi vesicle-mediated transport	4,3E-02	9,1E-01	9,0	PACS1, COG5, GOSR1
& Trp metabolism	GO:0019442~tryptophan catabolic process to acetyl-CoA	3,5E-02	8,9E-01	57,1	TDO2, KYNU
	GO:0019748~secondary metabolic process	4,9E-02	9,2E-01	3,6	TDO2, KYNU, CYP1B1, ADH5, NADSYN1

PC-up signature

Category	GO term	*P* -value	Benjamini	Fold Enrichment	Genes
	GO:0006916~anti-apoptosis	1,7E-05	1,8E-03	5,9	LOC400750, HSP90B1, SYVN1, MCL1, TXNDC5, DAD1, UBC, UBB, PIM2, HSPA5, UBA52, CD27
	GO:0012501~programmed cell death	3,4E-03	1,2E-01	2,5	MCL1, TMBIM6, PIM2, CTNNB1, CLPTM1L, CDKN1B, DAD1, UBC, UBB, ACIN1, SRGN, CD27, UBA52, TP53INP1
	GO:0008219~cell death	1,3E-02	2,9E-01	2,1	MCL1, TMBIM6, PIM2, CTNNB1, CLPTM1L, CDKN1B, DAD1, UBC, UBB, ACIN1, SRGN, CD27, UBA52, TP53INP1
	GO:0016265~death	1,4E-02	2,9E-01	2,1	MCL1, TMBIM6, PIM2, CTNNB1, CLPTM1L, CDKN1B, DAD1, UBC, UBB, ACIN1, SRGN, CD27, UBA52, TP53INP1
Apoptosis	GO:0006915~apoptosis	8,1E-03	2,1E-01	2,4	MCL1, TMBIM6, PIM2, CTNNB1, CLPTM1L, DAD1, UBC, UBB, ACIN1, SRGN, CD27, UBA52, TP53INP1
(negative)	GO:0043066~negative regulation of apoptosis	1,8E-05	1,7E-03	4,3	SYVN1, MCL1, TMBIM6, PIM1, PIM2, LOC400750, HSP90B1, NME1, TXNDC5, UBC, DAD1, UBB, HSPA5, CD27, UBA52
	GO:0043069~negative regulation of programmed cell death	2,1E-05	1,9E-03	4,3	SYVN1, MCL1, TMBIM6, PIM1, PIM2, LOC400750, HSP90B1, NME1, TXNDC5, UBC, DAD1, UBB, HSPA5, CD27, UBA52
	GO:0060548~negative regulation of cell death	2,2E-05	1,8E-03	4,3	SYVN1, MCL1, TMBIM6, PIM1, PIM2, LOC400750, HSP90B1, NME1, TXNDC5, UBC, DAD1, UBB, HSPA5, CD27, UBA52
	GO:0042981~regulation of apoptosis	3,1E-04	1,9E-02	2,6	SYVN1, MCL1, TMBIM6, PIM1, PIM2, SLAMF7, CALR, LOC400750, HSP90B1, CDKN1B, NME1, TXNDC5, UBC, DAD1, UBB, HSPA5, BMP7, UBA52, CD27, TP53INP1
	GO:0043067~regulation of programmed cell death	3,5E-04	1,9E-02	2,6	SYVN1, MCL1, TMBIM6, PIM1, PIM2, SLAMF7, CALR, LOC400750, HSP90B1, CDKN1B, NME1, TXNDC5, UBC, DAD1, UBB, HSPA5, BMP7, UBA52, CD27, TP53INP1
	GO:0010941~regulation of cell death	3,6E-04	1,9E-02	2,6	SYVN1, MCL1, TMBIM6, PIM1, PIM2, SLAMF7, CALR, LOC400750, HSP90B1, CDKN1B, NME1, TXNDC5, UBC, DAD1, UBB, HSPA5, BMP7, UBA52, CD27, TP53INP1
	GO:0065003~macromolecular complex assembly	3,1E-04	1,8E-02	2,8	HIST1H2BC, HIST1H2BD, HIST1H2BE, CCDC88C, HIST1H2BG, HIST1H2AD, CDK9, RPL24, CALR, CTNNB1, HIST1H2BM, HIST1H2BN, HIST2H2BE, HIST1H2BI, RPS15, HIST1H2BJ, TUBA4A, PEBP1, HIST1H2AM, HIST3H2B
	GO:0043933~macromolecular complex subunit organization	6,3E-04	3,1E-02	2,6	HIST1H2BC, HIST1H2BD, HIST1H2BE, CCDC88C, HIST1H2BG, HIST1H2AD, CDK9, RPL24, CALR, CTNNB1, HIST1H2BM, HIST1H2BN, HIST2H2BE, HIST1H2BI, RPS15, HIST1H2BJ, TUBA4A, PEBP1, HIST1H2AM, HIST3H2B
	GO:0006323~DNA packaging	1,1E-06	2,8E-04	9,4	HIST1H2BC, HIST1H2BD, HIST1H2BE, HIST1H2AD, HIST1H2BG, HIST1H2BM, HIST1H2BN, HIST2H2BE, HIST1H2BI, HIST1H2BJ, ACIN1, HIST3H2BB, HIST1H2AM
	GO:0051276~chromosome organization	4,4E-03	1,4E-01	2,7	HIST1H2BC, HIST1H2BD, HIST1H2BE, HIST1H2AD, HIST1H2BG, HIST1H2BM, HIST1H2BN, HIST2H2BE, HIST1H2BI, SMARCC2, HIST1H2BJ, ACIN1, HIST1H2AM, HIST3H2BB, HDAC7
	GO:0006333~chromatin assembly or disassembly	2,1E-06	2,5E-04	8,7	HIST1H2BC, HIST1H2BD, HIST1H2BE, HIST1H2AD, HIST1H2BG, HIST1H2BM, HIST1H2BN, HIST2H2BE, HIST1H2BI, SMARCC2, HIST1H2BJ, HIST3H2BB, HIST1H2AM
Chromatin	GO:0006325~chromatin organization	2,2E-03	8,9E-02	3,2	HIST1H2BC, HIST1H2BD, HIST1H2BE, HIST1H2AD, HIST1H2BG, HIST1H2BM, HIST1H2BN, HIST2H2BE, HIST1H2BI, SMARCC2, HIST1H2BJ, HIST3H2BB, HIST1H2AM, HDAC7
assembly	GO:0034622~cellular macromolecular complex assembly	3,0E-05	2,2E-03	4,5	HIST1H2BC, HIST1H2BD, HIST1H2BE, HIST1H2BG, HIST1H2AD, RPL24, CALR, HIST1H2BM, HIST1H2BN, HIST2H2BE, HIST1H2BI, RPS15, HIST1H2BJ, TUBA4A, HIST1H2AM, HIST3H2BB
	GO:0034621~cellular macromolecular complex subunit organization	9,1E-05	6,4E-03	4,0	HIST1H2BC, HIST1H2BD, HIST1H2BE, HIST1H2BG, HIST1H2AD, RPL24, CALR, HIST1H2BM, HIST1H2BN, HIST2H2BE, HIST1H2BI, RPS15, HIST1H2BJ, TUBA4A, HIST1H2AM, HIST3H2BB
	GO:0006334~nucleosome assembly	8,5E-07	3,0E-04	11,8	HIST1H2BC, HIST1H2BM, HIST1H2BD, HIST1H2BN, HIST1H2BE, HIST2H2BE, HIST1H2BG, HIST1H2BI, HIST1H2AD, HIST1H2BJ, HIST1H2AM, HIST3H2BB
	GO:0031497~chromatin assembly	1,1E-06	2,4E-04	11,4	HIST1H2BC, HIST1H2BM, HIST1H2BD, HIST1H2BN, HIST1H2BE, HIST2H2BE, HIST1H2BG, HIST1H2BI, HIST1H2AD, HIST1H2BJ, HIST1H2AM, HIST3H2BB
	GO:0065004~protein-DNA complex assembly	1,6E-06	2,8E-04	10,9	HIST1H2BC, HIST1H2BM, HIST1H2BD, HIST1H2BN, HIST1H2BE, HIST2H2BE, HIST1H2BG, HIST1H2BI, HIST1H2AD, HIST1H2BJ, HIST1H2AM, HIST3H2BB
	GO:0034728~nucleosome organization	1,9E-06	2,8E-04	10,6	HIST1H2BC, HIST1H2BM, HIST1H2BD, HIST1H2BN, HIST1H2BE, HIST2H2BE, HIST1H2BG, HIST1H2BI, HIST1H2AD, HIST1H2BJ, HIST1H2AM, HIST3H2BB
	GO:0034613~cellular protein localization	3,3E-02	4,5E-01	2,4	AP1G1, ERP29, CALR, SSR4, SEC61A1, SSR2, SRGN, SSR3, CTNNB1
	GO:0070727~cellular macromolecule localization	3,4E-02	4,5E-01	2,4	AP1G1, ERP29, CALR, SSR4, SEC61A1, SSR2, SRGN, SSR3, CTNNB1
	GO:0008104~protein localization	1,3E-02	2,9E-01	2,0	AP1G1, RRBP1, ERP29, PDIA4, LMAN2, CALR, CTNNB1, PREB, HSP90B1, SEC22B, GNAS, SSR4, SEC61A1, SRGN, SSR2, SSR3
Protein	GO:0015031~protein transport	2,0E-02	3,6E-01	2,0	AP1G1, RRBP1, ERP29, PDIA4, LMAN2, CALR, PREB, HSP90B1, SEC22B, GNAS, SSR4, SEC61A1, SSR2, SSR3
transport	GO:0045184~establishment of protein localization	2,1E-02	3,7E-01	2,0	AP1G1, RRBP1, ERP29, PDIA4, LMAN2, CALR, PREB, HSP90B1, SEC22B, GNAS, SSR4, SEC61A1, SSR2, SSR3
	GO:0009306~protein secretion	3,2E-02	4,5E-01	10,6	ERP29, GNAS, PDIA4
	GO:0046907~intracellular transport	2,4E-03	8,9E-02	2,5	NUDT4, AP1G1, ERP29, CALR, PREB, TXNDC5, RPS15, SEC22B, GNAS, GOLGA5, SSR4, SEC61A1, SSR2, SSR3, FTL
	GO:0048193~Golgi vesicle transport	6,6E-03	1,9E-01	5,0	PREB, AP1G1, TXNDC5, SEC22B, GOLGA5, FTL
	GO:0006414~translational elongation	8,3E-19	8,7E-16	20,7	RPL13, RPL35, RPL27A, RPS9, EEF2, RPL24, RPS8, RPS28, RPL18A, RPS16, RPL13A, RPLP0, RPS15, RPL8, UBC, RPL3, UBB, RPL12, UBA52
Ribosome	GO:0042257~ribosomal subunit assembly	2,7E-02	4,2E-01	73,3	RPS15, RPL24
& translation	GO:0042254~ribosome biogenesis	2,4E-02	4,0E-01	4,5	RPS28, RPS16, RPLP0, RPS15, RPL24
	GO:0042274~ribosomal small subunit biogenesis	4,2E-03	1,4E-01	30,0	RPS28, RPS16, RPS15
	GO:0006412~translation	1,7E-12	8,9E-10	7,3	RRBP1, RPL13, PABPC4, RPL35, RPL27A, RPS9, EEF2, RPL24, RPS8, RPS28, RPL18A, RPS16, RPL13A, RPLP0, EIF3E, RPS15, RPL8, UBC, RPL3, UBB, RPL12, UBA52

Homeostasis	GO:0042592~homeostatic process	3,8E-02	4,9E-01	1,9	ALDOA, P4HB, MCL1, GRXCR1, PDIA5, PDIA4, CALR, CTNNB1, TXNDC11, HSP90B1, TXNDC5, ACIN1, FTL
	GO:0019725~cellular homeostasis	9,5E-03	2,4E-01	2,6	ALDOA, P4HB, TXNDC11, HSP90B1, MCL1, TXNDC5, GRXCR1, PDIA5, PDIA4, CALR, FTL
	GO:0045454~cell redox homeostasis	2,5E-04	1,7E-02	10,5	P4HB, TXNDC11, TXNDC5, GRXCR1, PDIA5, PDIA4
	GO:0051208~sequestering of calcium ion	2,7E-02	4,2E-01	73,3	HSP90B1, CALR
Protein folding & catabolism	GO:0032269~negative regulation of cellular protein metabolic process	2,3E-02	3,9E-01	3,7	EIF3E, UBC, PEBP1, UBB, CALR, UBA52
	GO:0051248~negative regulation of protein metabolic process	2,7E-02	4,2E-01	3,5	EIF3E, UBC, PEBP1, UBB, CALR, UBA52
	GO:0030433~ER-associated protein catabolic process	2,0E-06	2,7E-04	27,5	HSP90B1, SYVN1, KIAA0368, UBC, UBB, UBA52
	GO:0043161~proteasomal ubiquitin-dependent protein catabolic process	2,3E-03	8,9E-02	6,5	HSP90B1, SYVN1, KIAA0368, UBC, UBB, UBA52
	GO:0010498~proteasomal protein catabolic process	2,3E-03	8,9E-02	6,5	HSP90B1, SYVN1, KIAA0368, UBC, UBB, UBA52
	GO:0044092~negative regulation of molecular function	3,2E-02	4,5E-01	2,6	LOC400750, CDKN1B, RGS1, PIM1, UBC, UBB, HSPA5, UBA52, TRIB1
	GO:0043086~negative regulation of catalytic activity	3,9E-02	4,9E-01	2,8	LOC400750, CDKN1B, RGS1, UBC, UBB, HSPA5, UBA52, TRIB1
	GO:0006457~protein folding	1,1E-03	4,9E-02	5,0	TOR3A, HSP90B1, PPIB, GRXCR1, ERP29, PDIA5, CALR, FKBP2
Cell communication	GO:0010647~positive regulation of cell communication	2,9E-03	1,0E-01	3,3	BST2, GOLPH3, UBC, PEBP1, UBB, PIM2, BMP7, UBA52, CD27, CTNNB1
	GO:0042062~long-term strengthening of neuromuscular junction	7,9E-04	3,7E-02	66,0	UBC, UBB, UBA52
	GO:0008582~regulation of synaptic growth at neuromuscular junction	1,6E-03	7,0E-02	47,1	UBC, UBB, UBA52
	GO:0050806~positive regulation of synaptic transmission	3,8E-02	4,8E-01	9,7	UBC, UBB, UBA52
	GO:0051971~positive regulation of transmission of nerve impulse	4,4E-02	5,2E-01	8,9	UBC, UBB, UBA52
	GO:0031646~positive regulation of neurological system process	4,8E-02	5,5E-01	8,5	UBC, UBB, UBA52
Differentiation	GO:0051963~regulation of synaptogenesis	1,1E-02	2,7E-01	18,3	UBC, UBB, UBA52
	GO:0050807~regulation of synapse organization	1,5E-02	3,1E-01	15,7	UBC, UBB, UBA52
	GO:0050803~regulation of synapse structure and activity	2,0E-02	3,6E-01	13,7	UBC, UBB, UBA52
	GO:0048742~regulation of skeletal muscle fiber development	2,1E-02	3,7E-01	13,2	UBC, UBB, UBA52
	GO:0048641~regulation of skeletal muscle tissue development	2,8E-02	4,2E-01	11,4	UBC, UBB, UBA52
	GO:0051153~regulation of striated muscle cell differentiation	3,4E-02	4,6E-01	10,3	UBC, UBB, UBA52
	GO:0007411~axon guidance	1,6E-02	3,2E-01	5,1	UBC, RPL24, UBB, BMP7, UBA52
	GO:0051147~regulation of muscle cell differentiation	4,8E-02	5,5E-01	8,5	UBC, UBB, UBA52
	GO:0045669~positive regulation of osteoblast differentiation	2,1E-02	3,7E-01	13,2	GNAS, BMP7, CTNNB1
	GO:0045667~regulation of osteoblast differentiation	6,8E-03	1,9E-01	10,2	GNAS, BMP7, HDAC7, CTNNB1
	GO:0030278~regulation of ossification	5,4E-03	1,6E-01	7,1	GNAS, BMP7, HDAC7, SRGN, CTNNB1
	GO:0045597~positive regulation of cell differentiation	1,8E-02	3,4E-01	3,4	NME1, GNAS, ACIN1, BMP7, CD27, FNDC3B, CTNNB1
	GO:0051094~positive regulation of developmental process	1,3E-02	2,9E-01	3,2	NME1, GNAS, ACIN1, BMP7, HDAC7, CD27, FNDC3B, CTNNB1
	GO:0002761~regulation of myeloid leukocyte differentiation	5,9E-03	1,7E-01	10,7	NME1, GNAS, ACIN1, CTNNB1
	GO:0045637~regulation of myeloid cell differentiation	2,5E-02	4,1E-01	6,3	NME1, GNAS, ACIN1, CTNNB1

Overlap PC-up & ASC signatures

Category	GO term	*P*-value	Benjamini	Fold Enrichment	Genes
Apoptosis (negative & positive)	GO:0006916~anti-apoptosis	1,3E-03	3,9E-01	9,9	HSP90B1, TXNDC5, UBC, UBB, HSPA5
	GO:0043066~negative regulation of apoptosis	9,3E-03	2,9E-01	5,8	HSP90B1, TXNDC5, UBC, UBB, HSPA5
	GO:0043069~negative regulation of programmed cell death	9,8E-03	2,6E-01	5,7	HSP90B1, TXNDC5, UBC, UBB, HSPA5
	GO:0060548~negative regulation of cell death	9,8E-03	2,5E-01	5,7	HSP90B1, TXNDC5, UBC, UBB, HSPA5
	GO:0042981~regulation of apoptosis	2,3E-03	2,5E-01	4,1	HSP90B1, TXNDC5, UBC, UBB, HSPA5, SLAMF7, CALR, TP53INP1
	GO:0043067~regulation of programmed cell death	2,5E-03	2,0E-01	4,0	HSP90B1, TXNDC5, UBC, UBB, HSPA5, SLAMF7, CALR, TP53INP1
	GO:0010941~regulation of cell death	2,5E-03	1,7E-01	4,0	HSP90B1, TXNDC5, UBC, UBB, HSPA5, SLAMF7, CALR, TP53INP1
	GO:0006917~induction of apoptosis	3,9E-02	4,1E-01	5,1	UBC, UBB, SLAMF7, TP53INP1
	GO:0012502~induction of programmed cell death	4,0E-02	4,0E-01	5,1	UBC, UBB, SLAMF7, TP53INP1
Protein transport	GO:0006605~protein targeting	1,4E-02	2,8E-01	7,6	CALR, SEC61A1, SSR2, SSR3
	GO:0006886~intracellular protein transport	1,1E-02	2,6E-01	5,5	CALR, SSR4, SEC61A1, SSR2, SSR3
	GO:0034613~cellular protein localization	1,5E-02	2,9E-01	5,0	CALR, SSR4, SEC61A1, SSR2, SSR3
	GO:0070727~cellular macromolecule localization	1,6E-02	2,8E-01	5,0	CALR, SSR4, SEC61A1, SSR2, SSR3
	GO:0015031~protein transport	8,1E-03	3,1E-01	3,8	HSP90B1, SEC22B, CALR, SSR4, SEC61A1, SSR2, SSR3
	GO:0045184~establishment of protein localization	8,4E-03	2,9E-01	3,7	HSP90B1, SEC22B, CALR, SSR4, SEC61A1, SSR2, SSR3
	GO:0008104~protein localization	1,6E-02	2,7E-01	3,3	HSP90B1, SEC22B, CALR, SSR4, SEC61A1, SSR2, SSR3
	GO:0006613~cotranslational protein targeting to membrane	2,8E-02	3,4E-01	68,3	SSR2, SSR3
	GO:0046907~intracellular transport	3,9E-03	2,2E-01	4,4	TXNDC5, SEC22B, CALR, SSR4, SEC61A1, SSR2, SSR3
	GO:0006465~signal peptide processing	1,9E-02	2,9E-01	102,5	SEC11C, SPCS1
Protein catabolism	GO:0051604~protein maturation	3,4E-02	3,9E-01	10,1	SEC11C, SPCS1, CALR
	GO:0006508~proteolysis	3,5E-02	3,9E-01	2,7	HSP90B1, SEC11C, UBC, UFC1, SPCS1, UBA5, UBB
	GO:0030433~ER-associated protein catabolic process	1,4E-03	2,4E-01	51,2	HSP90B1, UBC, UBB
	GO:0010498~proteasomal protein catabolic process	2,4E-02	3,3E-01	12,1	HSP90B1, UBC, UBB
	GO:0043161~proteasomal ubiquitin-dependent protein catabolic process	2,4E-02	3,3E-01	12,1	HSP90B1, UBC, UBB
	GO:0043632~modification-dependent macromolecule catabolic process	4,5E-02	4,1E-01	3,6	HSP90B1, UBC, UFC1, UBA5, UBB
	GO:0019941~modification-dependent protein catabolic process	4,5E-02	4,1E-01	3,6	HSP90B1, UBC, UFC1, UBA5, UBB
	GO:0043086~negative regulation of catalytic activity	2,7E-02	3,5E-01	5,9	UBC, UBB, HSPA5, TRIB1
	GO:0044092~negative regulation of molecular function	4,4E-02	4,1E-01	4,9	UBC, UBB, HSPA5, TRIB1
	GO:0042062~long-term strengthening of neuromuscular junction	1,2E-02	2,5E-01	164,0	UBC, UBB
	GO:0008582~regulation of synaptic growth at neuromuscular junction	1,6E-02	2,7E-01	117,1	UBC, UBB
	GO:0051963~regulation of synaptogenesis	4,2E-02	4,1E-01	45,5	UBC, UBB
	GO:0050807~regulation of synapse organization	4,9E-02	4,3E-01	39,0	UBC, UBB
Ion homeostasis	GO:0051208~sequestering of calcium ion	7,1E-03	3,1E-01	273,3	HSP90B1, CALR
	GO:0051238~sequestering of metal ion	1,6E-02	2,7E-01	117,1	HSP90B1, CALR
	GO:0019725~cellular homeostasis	2,3E-02	3,3E-01	4,4	P4HB, TXNDC11, HSP90B1, TXNDC5, CALR
	GO:0045454~cell redox homeostasis	9,7E-03	2,8E-01	19,5	P4HB, TXNDC11, TXNDC5

Overlap Top2000 PC-upregulated genes & ASC signature

Category	GO term	*P*-value	Benjamini	Fold Enrichment	Genes
	GO:0006916~anti-apoptosis	2,9E-03	8,1E-02	6,1	ATF5, HSP90B1, TXNDC5, UBC, UBB, HSPA5
	GO:0043066~negative regulation of apoptosis	2,6E-02	3,0E-01	3,5	ATF5, HSP90B1, TXNDC5, UBC, UBB, HSPA5
	GO:0043069~negative regulation of programmed cell death	2,7E-02	3,0E-01	3,5	ATF5, HSP90B1, TXNDC5, UBC, UBB, HSPA5
	GO:0060548~negative regulation of cell death	2,8E-02	2,9E-01	3,5	ATF5, HSP90B1, TXNDC5, UBC, UBB, HSPA5
	GO:0043065~positive regulation of apoptosis	4,1E-03	9,4E-02	3,9	MAGED1, ERN1, UBC, RPS27L, UBB, SLAMF7, RPS6, TP53INP1
	GO:0043068~positive regulation of programmed cell death	4,2E-03	9,3E-02	3,8	MAGED1, ERN1, UBC, RPS27L, UBB, SLAMF7, RPS6, TP53INP1

Apoptosis	GO:0010942~positive regulation of cell death	4,3E-03	9,2E-02	3,8	MAGED1, ERN1, UBC, RPS27L, UBB, SLAMF7, RPS6, TP53INP1
	GO:0006917~induction of apoptosis	4,0E-03	1,0E-01	4,6	MAGED1, ERN1, UBC, RPS27L, UBB, SLAMF7, TP53INP1
	GO:0012502~induction of programmed cell death	4,0E-03	9,7E-02	4,5	MAGED1, ERN1, UBC, RPS27L, UBB, SLAMF7, TP53INP1
	GO:0042981~regulation of apoptosis	8,2E-05	3,9E-03	3,6	RPS27L, SLAMF7, CALR, RPS6, MAGED1, ATF5, HSP90B1, VCP, TXNDC5, ERN1, UBC, UBB, HSPA5, TP53INP1
	GO:0043067~regulation of programmed cell death	9,0E-05	4,0E-03	3,6	RPS27L, SLAMF7, CALR, RPS6, MAGED1, ATF5, HSP90B1, VCP, TXNDC5, ERN1, UBC, UBB, HSPA5, TP53INP1
	GO:0010941~regulation of cell death	9,4E-05	3,8E-03	3,6	RPS27L, SLAMF7, CALR, RPS6, MAGED1, ATF5, HSP90B1, VCP, TXNDC5, ERN1, UBC, UBB, HSPA5, TP53INP1
	GO:0008104~protein localization	2,3E-06	4,2E-04	4,0	CALR, SEC63, SSR1, OS9, TRAM2, HSP90B1, VCP, TMED2, SRPR, USO1, SEC22B, SSR4, KDELR1, SEC61A1, SSR2, COPE, SSR3
	GO:0015031~protein transport	1,9E-06	1,0E-03	4,4	CALR, SEC63, SSR1, TRAM2, HSP90B1, VCP, TMED2, SRPR, USO1, SEC22B, SSR4, KDELR1, SEC61A1, SSR2, COPE, SSR3
	GO:0045184~establishment of protein localization	2,1E-06	5,7E-04	4,3	CALR, SEC63, SSR1, TRAM2, HSP90B1, VCP, TMED2, SRPR, USO1, SEC22B, SSR4, KDELR1, SEC61A1, SSR2, COPE, SSR3
	GO:0046907~intracellular transport	9,9E-06	8,7E-04	4,4	CALR, SEC63, SSR1, VCP, SRPR, TXNDC5, USO1, SEC22B, SSR4, KDELR1, SEC61A1, COPE, SSR2, SSR3
	GO:0006465~signal peptide processing	3,7E-02	3,5E-01	52,0	SEC11C, SPCS1
	GO:0006605~protein targeting	5,2E-04	2,0E-02	6,8	SRPR, CALR, SEC61A1, SSR2, SEC63, SSR1, SSR3
	GO:0045047~protein targeting to ER	4,2E-02	3,7E-01	46,2	SRPR, SEC61A1
Protein transport	GO:0006612~protein targeting to membrane	1,7E-05	1,3E-03	31,5	SRPR, SSR2, SEC63, SSR1, SSR3
	GO:0006613~cotranslational protein targeting to membrane	2,2E-05	1,3E-03	69,4	SRPR, SSR2, SSR1, SSR3
	GO:0006886~intracellular protein transport	9,3E-06	9,8E-04	6,1	VCP, SRPR, USO1, CALR, SSR4, KDELR1, SEC61A1, SSR2, SEC63, SSR1, SSR3
	GO:0034613~cellular protein localization	2,1E-05	1,4E-03	5,6	VCP, SRPR, USO1, CALR, SSR4, KDELR1, SEC61A1, SSR2, SEC63, SSR1, SSR3
	GO:0070727~cellular macromolecule localization	2,2E-05	1,2E-03	5,5	VCP, SRPR, USO1, CALR, SSR4, KDELR1, SEC61A1, SSR2, SEC63, SSR1, SSR3
	GO:0048193~Golgi vesicle transport	2,4E-02	3,0E-01	6,4	VCP, TXNDC5, SEC22B, COPE
	GO:0051235~maintenance of location	3,4E-03	9,2E-02	13,0	HSP90B1, CALR, KDELR1, OS9
	GO:0006621~protein retention in ER lumen	3,3E-02	3,2E-01	59,5	KDELR1, OS9
	GO:0006457~protein folding	1,5E-03	4,5E-02	7,1	HSP90B1, DNAJB11, CALR, FKBP11, SEC63, FKBP2
	GO:0030433~ER-associated protein catabolic process	4,5E-06	6,0E-04	43,4	HSP90B1, VCP, UBC, UBB, OS9
	GO:0010498~proteasomal protein catabolic process	1,4E-03	4,5E-02	10,2	HSP90B1, VCP, UBC, UBB, OS9
	GO:0043161~proteasomal ubiquitin-dependent protein catabolic process	1,4E-03	4,5E-02	10,2	HSP90B1, VCP, UBC, UBB, OS9
	GO:0006511~ubiquitin-dependent protein catabolic process	2,8E-02	3,0E-01	4,3	HSP90B1, VCP, UBC, UBB, OS9
Protein catabolism	GO:0044265~cellular macromolecule catabolic process	2,0E-02	2,8E-01	2,6	HSP90B1, VCP, UBE2J1, UBC, UFC1, UBA5, UBB, ISG20, OS9
	GO:0009057~macromolecule catabolic process	3,0E-02	3,1E-01	2,4	HSP90B1, VCP, UBE2J1, UBC, UFC1, UBA5, UBB, ISG20, OS9
	GO:0019941~modification-dependent protein catabolic process	1,8E-02	2,8E-01	2,9	HSP90B1, VCP, UBE2J1, UBC, UFC1, UBA5, UBB, OS9
	GO:0043632~modification-dependent macromolecule catabolic process	1,8E-02	2,8E-01	2,9	HSP90B1, VCP, UBE2J1, UBC, UFC1, UBA5, UBB, OS9
	GO:0051603~proteolysis involved in cellular protein catabolic process	2,3E-02	3,0E-01	2,8	HSP90B1, VCP, UBE2J1, UBC, UFC1, UBA5, UBB, OS9
	GO:0044257~cellular protein catabolic process	2,3E-02	3,0E-01	2,8	HSP90B1, VCP, UBE2J1, UBC, UFC1, UBA5, UBB, OS9
	GO:0030163~protein catabolic process	2,7E-02	3,1E-01	2,7	HSP90B1, VCP, UBE2J1, UBC, UFC1, UBA5, UBB, OS9
	GO:0006986~response to unfolded protein	4,6E-03	9,3E-02	11,7	VCP, ERN1, DNAJC3, MANF
Response to	GO:0051789~response to protein stimulus	1,4E-02	2,3E-01	7,8	VCP, ERN1, DNAJC3, MANF
unfolding & ER	GO:0006984~ER-nuclear signaling pathway	1,2E-02	2,2E-01	17,8	VCP, ERN1, HSPA5
	GO:0034976~response to endoplasmic reticulum stress	5,4E-04	1,9E-02	24,5	VCP, ERN1, HSPA5, OS9
	GO:0016567~protein ubiquitination	1,9E-02	2,7E-01	7,0	VCP, UBC, UBB, OS9
	GO:0032446~protein modification by small protein conjugation	2,5E-02	2,9E-01	6,3	VCP, UBC, UBB, OS9
Protein modification	GO:0070647~protein modification by small protein conjugation or removal	4,0E-02	3,6E-01	5,2	VCP, UBC, UBB, OS9
(Ub, Glycosylation)	GO:0018196~peptidyl-asparagine modification	4,6E-02	4,0E-01	41,6	STT3A, DDOST
	GO:0018279~protein amino acid N-linked glycosylation via asparagine	4,6E-02	4,0E-01	41,6	STT3A, DDOST
	GO:0006487~protein amino acid N-linked glycosylation	1,8E-02	2,7E-01	14,2	STT3A, FUT8, DDOST
	GO:0042062~long-term strengthening of neuromuscular junction	2,3E-02	3,0E-01	83,2	UBC, UBB
	GO:0008582~regulation of synaptic growth at neuromuscular junction	3,3E-02	3,2E-01	59,5	UBC, UBB
Others	GO:0045454~cell redox homeostasis	3,6E-02	3,4E-01	9,9	P4HB, TXNDC11, TXNDC5
	GO:0006414~translational elongation	1,2E-02	2,1E-01	8,2	RPL15, UBC, UBB, RPS6
	GO:0051208~sequestering of calcium ion	1,4E-02	2,4E-01	138,7	HSP90B1, CALR
	GO:0051238~sequestering of metal ion	3,3E-02	3,2E-01	59,5	HSP90B1, CALR

BPC-up signature

Category	GO term	*P* -value	Benjamini	Fold Enrichment	Genes
	GO:0007049~cell cycle	9,4E-08	5,2E-05	6,3	E2F2, KIFC1, CLSPN, RAN, MSH2, GMNN, BIRC5, SMC2, SPC24, PSMA2, SPC25, TUBB, MCM8, ZWINT
	GO:0051726~regulation of cell cycle	1,3E-02	3,6E-01	5,2	E2F2, MSH2, ZWINT, GMNN, BIRC5
	GO:0000075~cell cycle checkpoint	2,7E-02	5,3E-01	11,4	MSH2, ZWINT, BIRC5
	GO:0022402~cell cycle process	2,0E-05	1,8E-03	6,1	SPC24, PSMA2, SPC25, KIFC1, TUBB, MSH2, RAN, ZWINT, BIRC5, SMC2
Cell cycle	GO:0000278~mitotic cell cycle	6,9E-06	7,7E-04	8,4	SPC24, PSMA2, SPC25, KIFC1, TUBB, RAN, ZWINT, BIRC5, SMC2
& mitosis	GO:0051301~cell division	1,6E-04	8,7E-03	8,2	SPC24, SPC25, KIFC1, RAN, ZWINT, BIRC5, SMC2
	GO:0000280~nuclear division	2,2E-06	6,2E-04	12,6	SPC24, SPC25, KIFC1, TUBB, RAN, ZWINT, BIRC5, SMC2
	GO:0007067~mitosis	2,2E-06	6,2E-04	12,6	SPC24, SPC25, KIFC1, TUBB, RAN, ZWINT, BIRC5, SMC2
	GO:0000087~M phase of mitotic cell cycle	2,5E-06	4,7E-04	12,4	SPC24, SPC25, KIFC1, TUBB, RAN, ZWINT, BIRC5, SMC2
	GO:0048285~organelle fission	2,9E-06	4,1E-04	12,1	SPC24, SPC25, KIFC1, TUBB, RAN, ZWINT, BIRC5, SMC2
	GO:0000279~M phase	3,1E-05	2,5E-03	8,4	SPC24, SPC25, KIFC1, TUBB, RAN, ZWINT, BIRC5, SMC2
	GO:0022403~cell cycle phase	1,3E-04	8,1E-03	6,7	SPC24, SPC25, KIFC1, TUBB, RAN, ZWINT, BIRC5, SMC2
	GO:0006259~DNA metabolic process	1,3E-02	3,7E-01	4,1	CLSPN, MCM8, MSH2, RAN, RRM2, PCNA
DNA	GO:0006260~DNA replication	1,6E-02	3,8E-01	7,3	CLSPN, MCM8, RRM2, PCNA
replication,	GO:0051052~regulation of DNA metabolic process	4,1E-02	6,7E-01	9,1	MSH2, GMNN, PCNA
segregation	GO:0000070~mitotic sister chromatid segregation	4,6E-03	1,9E-01	28,9	KIFC1, ZWINT, SMC2
	GO:0000819~sister chromatid segregation	4,8E-03	1,9E-01	28,1	KIFC1, ZWINT, SMC2
	GO:0007059~chromosome segregation	7,5E-05	5,2E-03	21,4	SPC25, KIFC1, ZWINT, BIRC5, SMC2
	GO:0007017~microtubule-based process	5,4E-03	1,9E-01	6,9	SPC25, KIFC1, TUBB, RAN, ZWINT
Microtubule	GO:0007052~mitotic spindle organization	4,1E-02	6,6E-01	46,2	SPC25, RAN
organization	GO:0007051~spindle organization	2,7E-04	1,3E-02	30,8	SPC25, TUBB, RAN, ZWINT
	GO:0000226~microtubule cytoskeleton organization	8,0E-03	2,6E-01	9,4	SPC25, TUBB, RAN, ZWINT
RNA catabolism	GO:0006401~RNA catabolic process	1,4E-02	3,6E-01	16,0	DCPS, PPP1R8, RNASE6
