# Supplementary material for: The transcriptional profiling of human in vivo-generated plasma cells identifies selective imbalances in monoclonal gammopathies
Source: PLoS One. 2017 Aug 17;12(8):e0183264. doi: 10.1371/journal.pone.0183264 (PMC5560601; doi:10.1371/journal.pone.0183264)
Supplement: S3 Table — See main text and legend of Fig 3 for further details. (PDF) [file pone.0183264.s005.pdf]

Supplementary Table 3. Common PC genes between human and mouse. See main text and legend of Figure 3 for further details.

Gene	*PC-up*
ATAT1	x
ATF5	
BST2	x
CALR	x
CDV3	
CLPTM1L	x
COPE	
DDOST	x
DENND5B	
DNAJB11	
DNAJC3	x
ELL2	x
ERN1	
FAM46C	x
FKBP11	
FKBP2	x
FNDC3B	x
FUT8	
HIST1H1C	
HM13	x
HSP90B1	x
HSPA13	
HSPA5	x
HYOU1	
IDH2	
ISG20	
ITM2C	x
KDELR1	
KRTCAP2	x
MAGED1	
MANF	x
MTDH	
MZB1	x
NDUFA1	
OS9	
P4HB	x
PRDM1	
REXO2	
RPL15	
RPS27L	
RPS6	
SDF2L1	x
SEC11C	x
SEC22B	x
SEC61A1	x
SEC63	
SLAMF7	x
SLC35B1	
SLC39A11	
SLC39A7	x
SLC44A1	x
SLC7A5	x
SND1	x
SNORD14E	x
SNORD68	
SPCS1	x
SRPR	
SRPRB	x
SSR1	
SSR2	x
SSR3	x
SSR4	x
STT3A	
SURF4	
TMED2	
TMED9	x
TNFRSF17	
TP53INP1	x
TRAM2	
TRIB1	x
TXNDC11	x
TXNDC5	x
UBA5	x
UBB	x
UBC	x
UBE2J1	
UFC1	x
USO1	
VCP	
VIMP	x
XBP1	x
ZBP1	
