# Supplementary material for: The transcriptional profiling of human in vivo-generated plasma cells identifies selective imbalances in monoclonal gammopathies
Source: PLoS One. 2017 Aug 17;12(8):e0183264. doi: 10.1371/journal.pone.0183264 (PMC5560601; doi:10.1371/journal.pone.0183264)
Supplement: S4 Table — Differentially expressed genes (adjusted P-value < 0.1) in the pair-wise comparisons between PC subtypes. Black, specific genes; red, common genes (see main text). (PDF) [file pone.0183264.s006.pdf]

Supplementary Table 4. PC subtypes signatures: Differentially expressed genes (adjusted P-value < 0.1) in the pair-wise comparisons between PC subtypes. Black, specific genes; red, common genes (see main text).

BPC-UP SIGNATURE

ProbeName	log2FC BPC/TPC	adj.P-Val BPC/TPC	log2FC BPC/BMPC	adj.P-Val BPC/BMPC	Gene Symbol	Gene description	Refseq & others	UNIGENE_ID
A_23_P317620	4,61	0,012	4,20	0,036	ARL4C	ADP-ribosylation factor-like 4C	NM_005737	Hs.709513
A_23_P118815	3,04	0,012	2,60	0,052	BIRC5	baculoviral IAP repeat containing 5	NM_001012271	Hs.514527
A_24_P345993	5,27	0,030	4,71	0,078	CANX	calnexin	NM_001746	Hs.699155
A_24_P58759	3,79	0,047	4,38	0,038	CCT5P1		XR_018279	
A_23_P109988	2,59	0,047	2,60	0,070	CD86	CD86 molecule	NM_006889	Hs.171182
A_23_P126212	4,53	0,016	3,87	0,064	CLSPN	claspin	NM_022111	Hs.175613
A_24_P184799	4,17	0,012	3,65	0,042	COCH	cochlin	NM_004086	Hs.21016
A_23_P83624	4,98	0,021	4,83	0,046	COTL1	coactosin-like 1 (Dictyostelium)	NM_021149	Hs.289092
A_32_P462013	3,73	0,019	3,31	0,062	CRACR2A	BCL6 co-repressor-like 1	AK021694	Hs.504534
A_24_P115762	2,96	0,098	3,17	0,099	CTSC	cathepsin C	NM_148170	Hs.128065
A_23_P52738	2,01	0,040	2,52	0,015	DCPS	decapping enzyme, scavenger	NM_014026	Hs.504249
A_23_P30377	3,19	0,039	2,99	0,079	DDX46	DEAD (Asp-Glu-Ala-Asp) box polypeptide 46	NM_014829	Hs.406549
A_24_P343095	4,33	0,006	3,87	0,016	DHFR	dihydrofolate reductase	NM_000791	Hs.648635
A_23_P500390	2,48	0,056	2,44	0,084	DONSON	downstream neighbor of SON	NM_017613	Hs.436341
A_23_P408955	4,63	0,050	4,49	0,081	E2F2	E2F transcription factor 2	NM_004091	Hs.194333
A_23_P9574	2,10	0,037	2,00	0,070	ECT2	epithelial cell transforming sequence 2 oncogene	NM_018098	Hs.518299
A_23_P215009	4,02	0,008	3,86	0,015	FAM65B	family with sequence similarity 65, member B"	AB002384	Hs.559459
A_23_P358394	0,00		4,34	0,058	FAM65B	family with sequence similarity 65, member B	NM_015864	Hs.559459
A_24_P220575	3,28	0,015	2,75	0,066	FHP1	diacylglycerol kinase, eta	BC044822	Hs.659437
A_23_P19712	1,98	0,029	2,36	0,015	GMNN	geminin, DNA replication inhibitor	NM_015895	Hs.234896
A_32_P87697			6,06	0,001	HLA-DRA	major histocompatibility complex, class II, DR alpha	NM_019111	Hs.520048
A_24_P364263	6,20	0,000	5,65	0,000	HRASLS2	HRAS-like suppressor 2	NM_017878	Hs.272805
A_23_P133956	7,24	0,000	6,51	0,000	KIFC1	kinesin family member C1	NM_002263	Hs.436912
A_23_P50638	2,10	0,015			LRG1	leucine-rich alpha-2-glycoprotein 1	NM_052972	Hs.655559
A_23_P363896			3,67	0,000	LRRC75A-AS1	LRRC75A antisense RNA 1	NR_027162	Hs.368934
A_24_P312072	4,72	0,014	4,19	0,055	LYRM4	LYR motif containing 4	NM_020408	Hs.709318
A_23_P68547	2,98	0,083	3,13	0,089	MCM8	minichromosome maintenance complex component 8	NM_182802	Hs.631506
A_23_P102471	2,30	0,031	2,15	0,061	MSH2	mutS homolog 2	NM_000251	Hs.597656
A_23_P64010	5,87	0,035	5,34	0,081	MTCH2	mitochondrial carrier homolog 2 (C. elegans)	NM_014342	Hs.269944
A_23_P313993	1,17	0,055	1,12	0,091	PAX2	paired box 2	L25597	Hs.155644
A_23_P28886	4,97	0,042	5,15	0,058	PCNA	proliferating cell nuclear antigen	NM_002592	Hs.147433
A_24_P127462	3,99	0,036	3,97	0,060	PCNAP3		XR_018314	
A_32_P147241	2,12	0,090	4,48	0,000	PKM	pyruvate kinase, muscle"	NM_182470	Hs.534770
A_23_P12272	3,77	0,007	3,54	0,015	PPP1R8	protein phosphatase 1, regulatory subunit 8	NM_138558	Hs.533474
A_23_P256432	4,13	0,097	4,76	0,075	PPP2R5A	protein phosphatase 2, regulatory subunit B', alpha isoform"	NM_006243	Hs.716433
A_23_P207307	2,14	0,095	2,84	0,042	PRR11	proline rich 11	AK001891	Hs.631750
A_23_P345591	4,28	0,078	5,21	0,046	PSMA2	proteasome (prosome, macropain) subunit, alpha type, 2	NM_002787	Hs.333786
A_24_P587882	3,94	0,012	3,87	0,015	PSMA2P2			
A_24_P47547	5,13	0,070			RAN	RAN, member RAS oncogene family	NM_006325	Hs.10842
A_32_P506600	3,40	0,068	3,55	0,076	RAN	RAN, member RAS oncogene family	NM_006325	Hs.10842
A_23_P209944	2,50	0,059	2,75	0,058	RETSAT	retinol saturase (all-trans-retinol 13,14-reductase)	NM_017750	Hs.440401
A_23_P3014	2,44	0,083	3,32	0,021	RNASE6	ribonuclease, RNase A family, k6	NM_005615	Hs.23262
A_24_P234196	7,08	0,000	6,26	0,000	RRM2	ribonucleotide reductase M2 polypeptide	NM_001034	Hs.226390
A_23_P103522	3,00	0,030	3,11	0,045	SELL	selectin L	NM_000655	Hs.82848

A_32_P96719	4,96	0,007	5,31	0,004	SHCBP1	SHC SH2-domain binding protein 1	NM_024745	Hs.123253
A_23_P308519	3,06	0,014	3,08	0,019	SLC9A3R1	solute carrier family 9 (sodium/hydrogen exchanger), member 3 reg	NM_004252	Hs.715489
A_23_P60271	3,51	0,013	3,39	0,024	SMC2	structural maintenance of chromosomes 2	NM_001042550	Hs.119023
A_23_P123874	3,23	0,095			SMU1	smu-1 suppressor of mec-8 and unc-52 homolog (C. elegans)	NM_018225	Hs.655351
A_32_P68746	3,22	0,030	3,02	0,066	SMU1	smu-1 suppressor of mec-8 and unc-52 homolog (C. elegans)	NM_018225	Hs.655351
A_24_P314571	5,85	0,002	3,71	0,070	SPC24	SPC24, NDC80 kinetochore complex component	ENST0000059254	Hs.381225
A_23_P51085	2,72	0,004	2,54	0,010	SPC25	SPC25, NDC80 kinetochore complex component	NM_020675	Hs.421956
A_24_P407645	4,16	0,030	4,83	0,016	SPN	sialophorin	NM_001030288	Hs.632188
A_23_P346093	4,41	0,042	4,50	0,060	TMC8	transmembrane channel-like 8	NM_152468	Hs.592102
A_24_P242076	3,56	0,092	3,94	0,079	TMEM50A		AK074960	
A_23_P84705	5,91	0,022			TNFRSF13B	tumor necrosis factor receptor superfamily, member 13B	NM_012452	Hs.158341
A_23_P37736			3,67	0,069	TNFRSF17	tumor necrosis factor receptor superfamily, member 17	NM_001192	Hs.2556
A_23_P132226	3,27	0,072	3,56	0,066	TPST2	tyrosylprotein sulfotransferase 2	NM_001008566	Hs.716478
A_23_P323761	4,33	0,025	4,99	0,015	TRAF3IP3	TRAF3 interacting protein 3	NM_025228	Hs.147434
A_23_P334414	3,84	0,028	3,86	0,046	TRAF3IP3	TRAF3 interacting protein 3	NM_025228	Hs.147434
A_23_P104025	1,92	0,094	2,52	0,042	TSEN15	TSEN15 tRNA splicing endonuclease subunit	NM_052965	Hs.548197
A_23_P387057	4,03	0,017	4,80	0,008	TUBB	tubulin, beta class I	NM_178014	Hs.636480
A_23_P81912	3,35	0,090			TUBB	tubulin, beta class I	NM_178014	Hs.636480
A_23_P68866	3,70	0,096	4,17	0,079	UQCR10	ubiquinol-cytochrome c reductase, complex III subunit X	NM_001003684	Hs.284292
A_23_P99405	2,66	0,042	2,89	0,046	ZMYM2	zinc finger, MYM-type 2	NM_003453	Hs.644041
A_23_P63789	4,23	0,012	5,29	0,001	ZWINT	ZW10 interacting kinetochore protein	NM_032997	Hs.591363

TPC-UP SIGNATURE

ProbeName	log2FC TPC/BPC	adj.P-ValTPC/BPC	log2FC TPC/BMPC	adj.P-Val tTPC/BMPC	Gene Symbol	Gene description	Refseq & others	UNIGENE_ID
A_23_P98686	3,800	0,081			ATHL1	ATH1, acid trehalase-like 1 (yeast)	NM_025092	Hs.353181
A_24_P288915	8,391	0,000	6,335	0,016	CCDC144A	coiled-coil domain containing 144B	ENST0000044575	Hs.649499
A_24_P49647	4,967	0,024			CCDC144A	coiled-coil domain containing 144A	AB011137	Hs.419859
A_24_P75917	7,377	0,000	5,80	0,012	CCDC144A	coiled-coil domain containing 144A	NM_014695	Hs.419859
A_32_P216369	4,309	0,073			CCDC144A	coiled-coil domain containing 144A	ENST0000036052	Hs.419859
A_23_P209055	7,046	0,000	8,64	0,000	CD22	CD22 molecule	NM_001771	Hs.579691
A_23_P351286	4,898	0,052			CD22	CD22 molecule	AK026467	
A_24_P659036	3,405	0,030	3,78	0,044	DENND6B	DENN/MADD domain containing 6B	NM_001001794	Hs.530380
A_23_P51856	4,327	0,070	5,45	0,051	DUSP10	dual specificity phosphatase 10	NM_007207	Hs.497822
A_24_P37409	3,601	0,070			DUSP2	dual specificity phosphatase 2	NM_004418	Hs.1183
A_23_P214080	5,243	0,002			EGR1	early growth response 1	NM_001964	Hs.326035
A_23_P347623	4,017	0,016			ERO1LB	ERO1-like beta (S. cerevisiae)	NM_019891	Hs.558519
A_23_P66827	4,235	0,001	3,46	0,016	FAM106A	family with sequence similarity 106, member A	NR_026809	Hs.674403
A_23_P80048	4,441	0,005			FER1L4	fer-1-like family member 4, pseudogene (functional)	NR_119376	Hs.72222
A_23_P429998	2,295	0,037			FOSB	FBJ murine osteosarcoma viral oncogene homolog B	NM_006732	Hs.590958
A_24_P350186	2,455	0,036			HID1	chromosome 17 open reading frame 28	NM_030630	Hs.11067
A_32_P87697			7,12	0,000	HLA-DRA	major histocompatibility complex, class II, DR alpha	NM_019111	Hs.520048
A_23_P403424	3,412	0,098			JMJD7-PLA2G4E	JMJD7-PLA2G4B readthrough	NM_005090	Hs.198161
A_24_P203418	1,542	0,076			LOC100131845	similar to hCG1742309	ENST0000061737	Hs.547094
A_23_P363896			2,68	0,016	LRRC75A-AS1	LRRC75A antisense RNA 1	NR_027162	Hs.368934
A_23_P118791	3,449	0,021			SNORD118	transmembrane protein 107	NM_032354	Hs.513933
A_23_P37736			4,11	0,068	TNFRSF17	tumor necrosis factor receptor superfamily, member 17	NM_001192	Hs.2556
A_23_P217688	3,109	0,055			TSC22D3	TSC22 domain family, member 3"	NM_004089	Hs.716410
A_23_P207666	4,211	0,016	3,76	0,097	USP32	ubiquitin specific peptidase 32	NM_032582	Hs.448851

A_23_P431630	3,717	0,016			USP32	ubiquitin specific peptidase 32	NM_032582	Hs.132868
A_24_P344537	1,599	0,048			ZNF625-ZNF20	zinc finger protein 625	NM_145233	Hs.512823
A_23_P101623	2,031	0,013			ZNF667	zinc finger protein 667	NM_022103	Hs.712574
A_24_P153207	2,152	0,010			ZNF850	zinc finger protein 850 pseudogene	CR627133	Hs.406307

BMPC-UP SIGNATURE

ProbeName	log2FC BMPC/BPC	adj.P-Val BMPC/BPC	log2FC BMPC/TPC	adj.P-ValBMPC/TPC	Gene Symbol	Gene description	Refseq & others	UNIGENE_ID
A_23_P98686	4,47	0,058			ATHL1	ATH1, acid trehalase-like 1 (yeast)	NM_025092	Hs.353181
A_23_P87982	3,16	0,015	2,67	0,068	ATP12A	ATPase, H+/K+ transporting, nongastric, alpha polypeptide	NM_001676	Hs.147111
A_24_P37409	3,78	0,076			DUSP2	dual specificity phosphatase 2	NM_004418	Hs.1183
A_23_P214080	5,51	0,001			EGR1	early growth response 1	NM_001964	Hs.326035
A_23_P347623	3,21	0,082			ERO1LB	ERO1-like beta (S. cerevisiae)	NM_019891	Hs.558519
A_23_P80048	5,88	0,000			FER1L4	fer-1-like family member 4, pseudogene (functional)	NR_119376	Hs.72222
A_24_P23454	5,42	0,060			FER1L4			
A_23_P429998	2,68	0,021			FOSB	FBJ murine osteosarcoma viral oncogene homolog B	NM_006732	Hs.590958
A_24_P350186	3,15	0,012			HID1	chromosome 17 open reading frame 28	NM_030630	Hs.11067
A_23_P403424	3,76	0,089			JMJD7-PLA2G4B	JMJD7-PLA2G4B readthrough	NM_005090	Hs.198161
A_24_P203418	1,63	0,079			LOC100131845	similar to hCG1742309	ENST0000061737	Hs.547094
A_23_P118791	3,53	0,032			SNORD118	transmembrane protein 107	NM_032354	Hs.513933
A_23_P84705			-5,757097209	0,074	TNFRSF13B	tumor necrosis factor receptor superfamily, member 13B	NM_012452	Hs.158341
A_23_P217688	3,35	0,060			TSC22D3	TSC22 domain family, member 3"	NM_004089	Hs.716410
A_24_P344537	1,56	0,079			ZNF625-ZNF20	zinc finger protein 625	NM_145233	Hs.512823
A_23_P101623	1,56	0,081			ZNF667	zinc finger protein 667	NM_022103	Hs.712574
A_24_P153207	1,72	0,058			ZNF850	zinc finger protein 850 pseudogene	CR627133	Hs.406307
